# Causal Links Between Renal Function and Cardiac Structure, Function, and Disease Risk

**DOI:** 10.5334/gh.1366

**Published:** 2024-11-06

**Authors:** Xiaoqin Zhou, Weiqiang Ruan, Lijun Zhao, Ke Lin, Jing Li, Huizhen Liu, Ting Wang, Guiying Zhang

**Affiliations:** 1 Department of Cardiovascular Surgery, West China Hospital, Sichuan University, Chengdu 610041, P.R. China; 2 Research Center of Clinical Epidemiology and Evidence-Based Medicine, West China Hospital, Sichuan University, Chengdu 610041, P.R. China; 3 Center of Biostatistics, Design, Measurement and Evaluation (CBDME), Department of Clinical Research Management, West China Hospital, Sichuan University, Chengdu 610041, P.R. China; 4 Department of General Practice Ward/International Medical Center Ward, General Practice Medical Center, West China Hospital, Sichuan University, Chengdu 610041, P.R. China; 5 Laboratory of diabetic kidney disease, Centre of diabetes and metabolism research, West China Hospital, Sichuan University, Chengdu 610041, P.R. China

**Keywords:** Mendelian randomization, Coronary artery disease, Stroke, Chronic kidney disease, Estimated glomerular filtration rate

## Abstract

**Background:**

Chronic kidney disease (CKD) increases the risk of adverse cardiovascular outcomes. However, the causal relationships between renal function and cardiovascular diseases (CVD) remain incompletely understood. This study aimed to determine the causal relationships between genetic susceptibility to impaired renal function and the risk of CVD endpoints, as well as cardiac structure and function detectable by cardiac magnetic resonance imaging (CMR).

**Methods:**

Bidirectional Mendelian randomization (MR) analyses were conducted using summary-level data from genome-wide association studies. The exposures were blood urea nitrogen (BUN), estimated glomerular filtration rate (eGFR), urine albumin-to-creatinine ratio (UACR), and CKD. The outcomes included atrial fibrillation, coronary artery disease (CAD), myocardial infarction, heart failure, stroke, and various CMR parameters. Sensitivity analyses, multivariable MR adjusting for cardiometabolic traits, and replication in the FinnGen cohort were performed.

**Results:**

Elevated BUN levels (OR 1.505; 95% CI 1.077 to 2.103; *P* = 0.017) were causally associated with increased CAD risk, but this relationship was attenuated after adjusting for cardiometabolic traits. Increased UACR was causally linked to higher risks of CAD (OR 1.260; 95% CI 1.042 to 1.523; *P =* 0.017), myocardial infarction (OR 1.424; 95% CI 1.137 to 1.783; *P =* 0.002), and stroke (OR 1.182; 95% CI 1.012 to 1.379; *P =* 0.035), with the association for stroke remaining significant after multivariable adjustment. Reduced eGFR was causally related to decreases in ascending aorta diameter, proximal pulmonary artery diameter, right atrial size, left ventricular stroke volume, and right ventricular volumes, even after accounting for potential confounders. CKD was causally associated with a reduced pulmonary artery-to-aorta ratio and proximal pulmonary artery diameter.

**Conclusions:**

This comprehensive MR study establishes causal roles of genetic susceptibility to impaired renal function influencing cardiovascular outcomes and cardiac structure.

**What was known:** CKD increases cardiovascular disease risk, but causal links were uncertain. There is limited research on causal effects of renal function on cardiac structure.

**This study adds:** Elevated BUN causally increases coronary artery disease risk. Albuminuria is an independent causal risk factor for stroke. Reduced eGFR/CKD causally contributes to pulmonary vascular alterations.

**Potential impact:** Supports albuminuria screening for cerebrovascular risk mitigation in CKD. Monitoring of pulmonary pressures/right heart in advanced CKD is warranted. Provides insights into kidney-heart crosstalk for future studies.

## Introduction

Chronic Kidney Disease (CKD), affecting approximately 10 to 15% of the global adult population, poses a significant public health challenge (1). CKD is characterized by long-term, progressive damage to the kidneys, resulting in a gradual loss of renal function over time. As CKD progresses, the kidneys’ ability to efficiently filter blood weakens. This deterioration in renal function leads to an accumulation of toxins and metabolic dysfunction, ultimately resulting in uremia. There is substantial evidence that CKD significantly increases the risk of adverse cardiovascular outcomes, including heart failure (HF), stroke, myocardial infarction (MI), and cardiovascular mortality (2). Critically, there exists a bidirectional causality in this relationship. Conditions that induce circulatory strain, notably hypertension, are known to exacerbate renal damage due to reduced renal perfusion and ischemia, thus reciprocally influencing the risk and progression of CKD and cardiovascular diseases (CVD) (3).

While randomized controlled trials (RCTs) are the gold standard for establishing causality, their application in the context of CKD and cardiovascular outcomes is often impeded by ethical, practical, or financial constraints. This limitation necessitates reliance on observational studies, which, despite their extensive utility, encounter challenges in definitively determining causal relationships due to inherent residual confounding. Furthermore, studies exploring the causal relationship between renal function and pathological cardiac remodeling (measured via cardiac magnetic resonance (CMR) phenotypes) are limited by sample size and financial barriers. To date, this issue has been predominantly explored through case-control studies (4).

Mendelian randomization (MR) leverages naturally occurring genetic variations, specifically single nucleotide polymorphisms (SNPs), as instrumental variables (IVs), providing a method akin to a natural experiment for strengthening causal inferences in observational data (5). This approach mirrors the methodology of RCTs by using the random segregation and independent assortment of alleles, as dictated by Mendel’s laws during meiosis, effectively creating a ‘natural randomized trial.’ SNPs, which have established associations with specific risk factors, biomarkers, or health outcomes, serve as proxies (5). They enable the simulation of treatment and control groups in traditional RCTs, thereby allowing for an unbiased estimation of the causal effects of various exposures. In the realm of cardiovascular research, MR has been increasingly utilized to unravel the intricate causal relationships between various risk factors and CVD, offering insights that surpass the limitations of conventional observational studies and addressing the challenges of RCTs, such as their often prohibitive cost and scale (6).

This study, through a bidirectional MR approach, aims to determine the causal relationships between genetic susceptibility to impaired renal function and the risk of CVD endpoints, as well as imaging parameters of cardiac structure and function detectable by CMR. This research could lay a theoretical foundation for the prevention and treatment of CKD and unveil novel perspectives.

## Methods

### Study design

**Figure 1:**
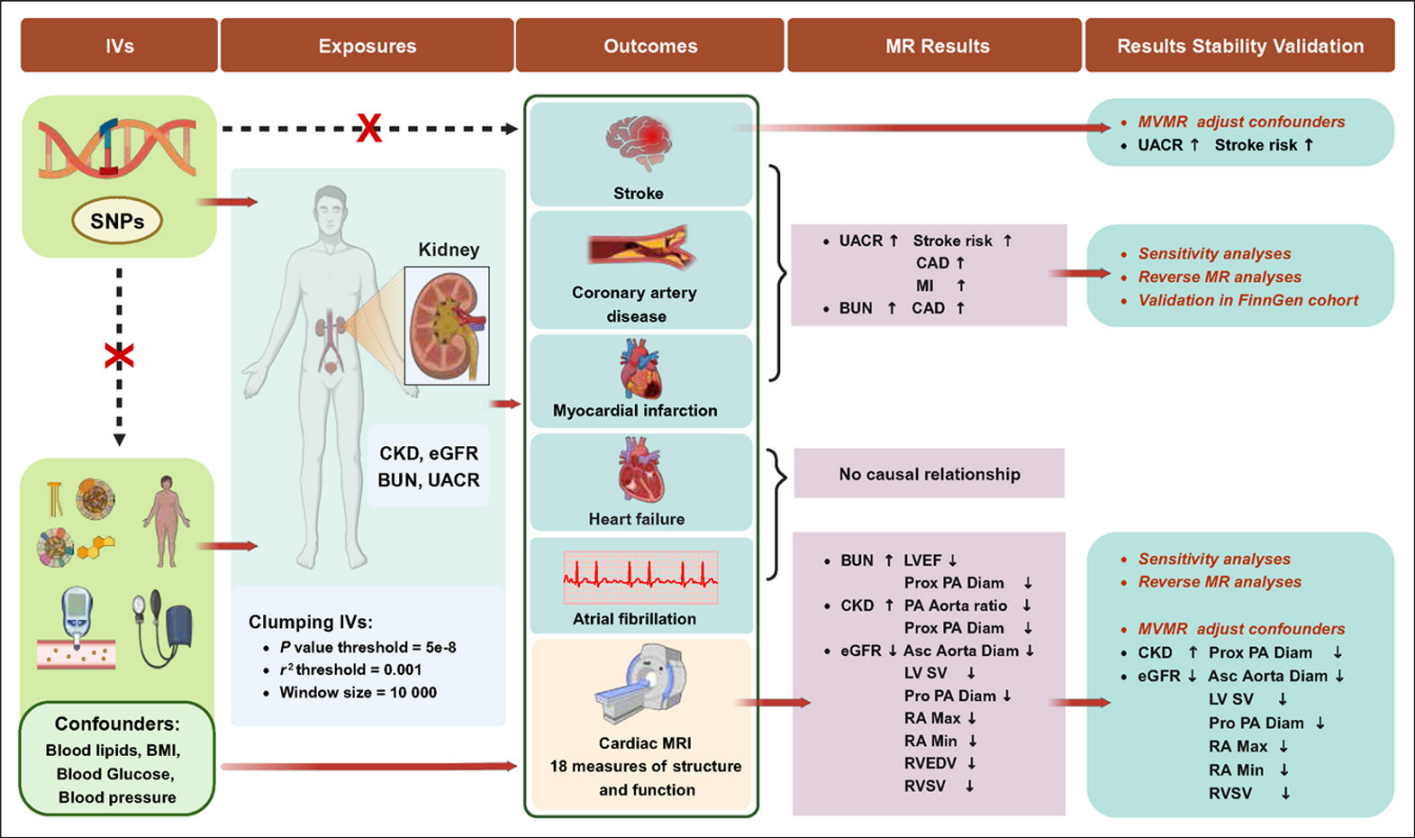
Schematic representation of MR analysis. MR, Mendelian randomization.

In our study, we conducted bidirectional MR analyses to investigate potential causal relationships between renal function and cardiovascular outcomes, as well as their influence on cardiac structure and function. To ensure the robustness of our findings, we implemented various MR methods and carried out reverse MR to evaluate possible reverse causality. To minimize the impact of confounding factors, we used multivariable MR to adjust for related cardiometabolic traits. For external validation of these cardiovascular endpoints, data from the FinnGen database were utilized. The sources of our data are detailed in Table S1. Additionally, our research follows the STROBE-MR Guidelines. Figure 1 provides a visual representation of our study’s procedural outline, which was created using BioRender.com.

### Data sources for Renal function

Our study utilized summary data from Genome-wide association studies (GWAS) for blood urea nitrogen (BUN), estimated glomerular filtration rate (eGFR), urine albumin-to-creatinine ratio (UACR), and CKD, obtained from the CKDGen Consortium. This data originated from the consortium’s cross-ethnic GWAS meta-analyses. We specifically focused on GWAS data for individuals of European ancestry. The meta-analysis for CKD GWAS encompassed 64,164 cases and 561,055 controls, all of European ancestry. CKD was defined as an eGFR below 60 ml/min/1.73 m2.

### Data sources for cardiac outcomes

The cardiovascular disease outcomes investigated in our study encompassed atrial fibrillation (AF), coronary artery disease (CAD), MI, HF, and stroke. We sourced the GWAS summary statistics for these endpoints from comprehensive meta-analyses conducted by leading genetic consortia. To externally validate these cardiovascular outcomes, we used summary data from a GWAS meta-analysis by the FinnGen consortium (R9 data release). For comprehensive details about these data sources, please refer to Table S1.

### Data sources for cardiac structure and function

We obtained data on key CMR structure and functional parameters from GWAS conducted among 36,000–45,000 European ancestry participants of the UK Biobank Imaging Study. These traits encompassed left atrial maximum volume (LA Max), left atrial total ejection fraction (LATEF), left ventricular mass (LV Mass), left ventricular end-diastolic volume (LVEDV), left ventricular end-systolic volume (LVESV), left ventricular stroke volume (LVSV), left ventricular ejection fraction (LVEF), degree of myocardial interstitial fibrosis, ascending aorta diameter (Asc Aorta Diam), pulmonary artery-to-aorta diameter ratio (PA Aorta ratio), proximal pulmonary artery diameter (Prox PA Diam), right atrial fractional area change (RA FAC), right atrial maximum area (RA Max), right atrial minimum area (RA Min), right ventricular ejection fraction (RVEF), right ventricular end-diastolic volume (RVEDV), right ventricular end-systolic volume (RVESV), right ventricular stroke volume (RVSV), and the ratio of right ventricular end-systolic to left ventricular end-systolic volume (RVESV LVESV ratio). To ensure comparability across different body sizes, all cardiac imaging measures were indexed to body surface area, except for dimensionless metrics like total ejection fraction and fractional area change.

### Data sources for Confounding Traits

To address potential confounding factors in our evaluation of causal relationships, we sourced GWAS summary statistics from the UK Biobank.

These statistics included data on low-density lipoprotein (LDL) cholesterol, high-density lipoprotein (HDL) cholesterol, triglycerides (TG), and Apolipoprotein A1 and Apolipoprotein B, as well as systolic and diastolic blood pressure and fasting glucose. For Body Mass Index (BMI) data, our source was a comprehensive meta-analysis focused on individuals of European ancestry.

### Instrumental variable selection

We carefully selected independent SNPs meeting stringent criteria to serve as IVs for MR analyses (5). IVs were required to have genome-wide significant associations with the exposure (*P* < 5e-8), reside in loci exhibiting no significant between-study heterogeneity, and be independent SNPs in linkage equilibrium (*r*^2^ < 0.001). Only SNPs with minor allele frequencies > 0.01 were included. For reverse MR examining SNPs associated with cardiac MRI structure and function, a *P-value* threshold of < 5e-7 was employed when the number of SNPs was limited (less than three for some traits). The *F-statistics* of chosen IVs were confirmed to be > 10, indicating sufficient strength as IVs (7). These rigorous criteria minimized the possibility of violating core MR assumptions. Details of the SNPs selected as IVs are provided in Table S2.

### Main MR analyses

In our study, we utilized a two-sample MR framework based on summary-level data. The primary method of analysis was inverse variance weighted (IVW) regression, operating under a random-effects model (8). This approach involved pooling Wald ratio estimates for each SNP via meta-analysis, using the inverse of their variance as weights to derive causal effect estimates.

We adjusted the significance threshold using Bonferroni correction to address multiple testing. This correction was applied only to the exposures, with the adjusted *P-value* threshold set at 0.0125 (0.05/4), considering the four exposures in our study. This approach to setting the *P-value* threshold aimed to reduce the risk of Type I errors while simultaneously mitigating Type II errors. Furthermore, if statistical significance was observed in both the primary and validation cohorts, we opted not to use the Bonferroni correction and maintained the *P-value* threshold at 0.05.

### Sensitivity analyses

To verify the validity of significant causal associations, we conducted a series of sensitivity analyses using complementary MR methods. We implemented several additional MR methods in sensitivity analyses, including MR-Egger regression, Weighted Median estimator, Simple Mode and Weighted Mode, to evaluate consistency and robustness. We evaluated the presence of horizontal pleiotropy via MR-Egger intercept tests (9). Heterogeneity was inspected using Cochran’s Q statistic, necessitating the adoption of random-effects models where significant (*P* < 0.05) (10). Global test *P-values* < 0.05 suggested the presence of outliers distorting causal estimates (11). If outliers were detected, we examined whether Outlier-Corrected Estimates significantly altered the results. We also performed reverse MR analysis, swapping the exposure and outcome, to explore any potential reverse causality.

### Multivariable MR analysis

We utilized multivariable MR to mitigate the impact of confounding factors on the causal relationships between exposure and outcomes. By adjusting for cardiovascular risk factors such as blood pressure, glucose, and lipid levels, we aimed to obtain robust outcomes and address potential confounding influences.

### Replication analysis

In our replication analysis for cardiovascular outcomes, we used data from the FinnGen cohort. Successful replication required statistical significance (*P* < 0.05) in the IVW method and consistent effect estimates across all methods.

### Analysis software

All analyses were performed in R version 4.2.3 (R Foundation for Statistical Computing, Vienna, Austria). We utilized the TwosampleMR (version 0.5.7) and MR-PRESSO (version 1.0) (11) packages for conducting main and sensitivity MR tests.

## Results

### Renal function and Cardiovascular Outcomes

Our MR analysis revealed a causal relationship between increased BUN levels and a higher risk of CAD in the primary cohort (odds ratio (OR) 1.505; 95% CI 1.077 to 2.103; *P =* 0.017). These findings were further corroborated in the FinnGen cohort for CAD (OR 1.840; 95% CI 1.148 to 2.948; *P =* 0.011). However, in the primary cohort, MR-Egger results did not reach statistical significance, and there was an inconsistency in the direction of the effect compared to the IVW method.

**Figure 2:**
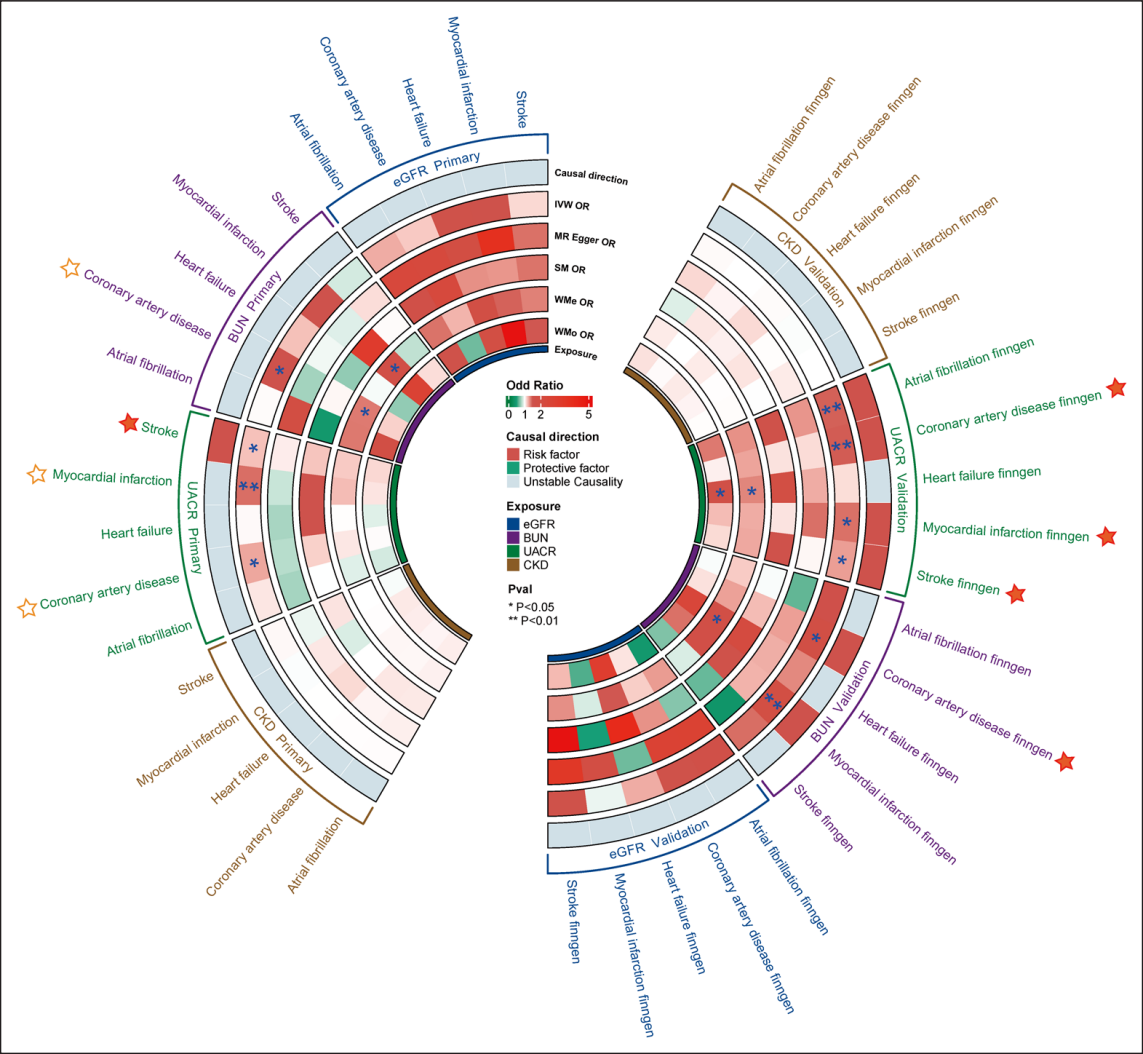
Causal effects of renal function on cardiovascular diseases. IVW, Inverse variance weighted; SM, Simple Mode; WME, Weighted Median Estimator; WMo, Weighted Mode.

**Figure 3:**
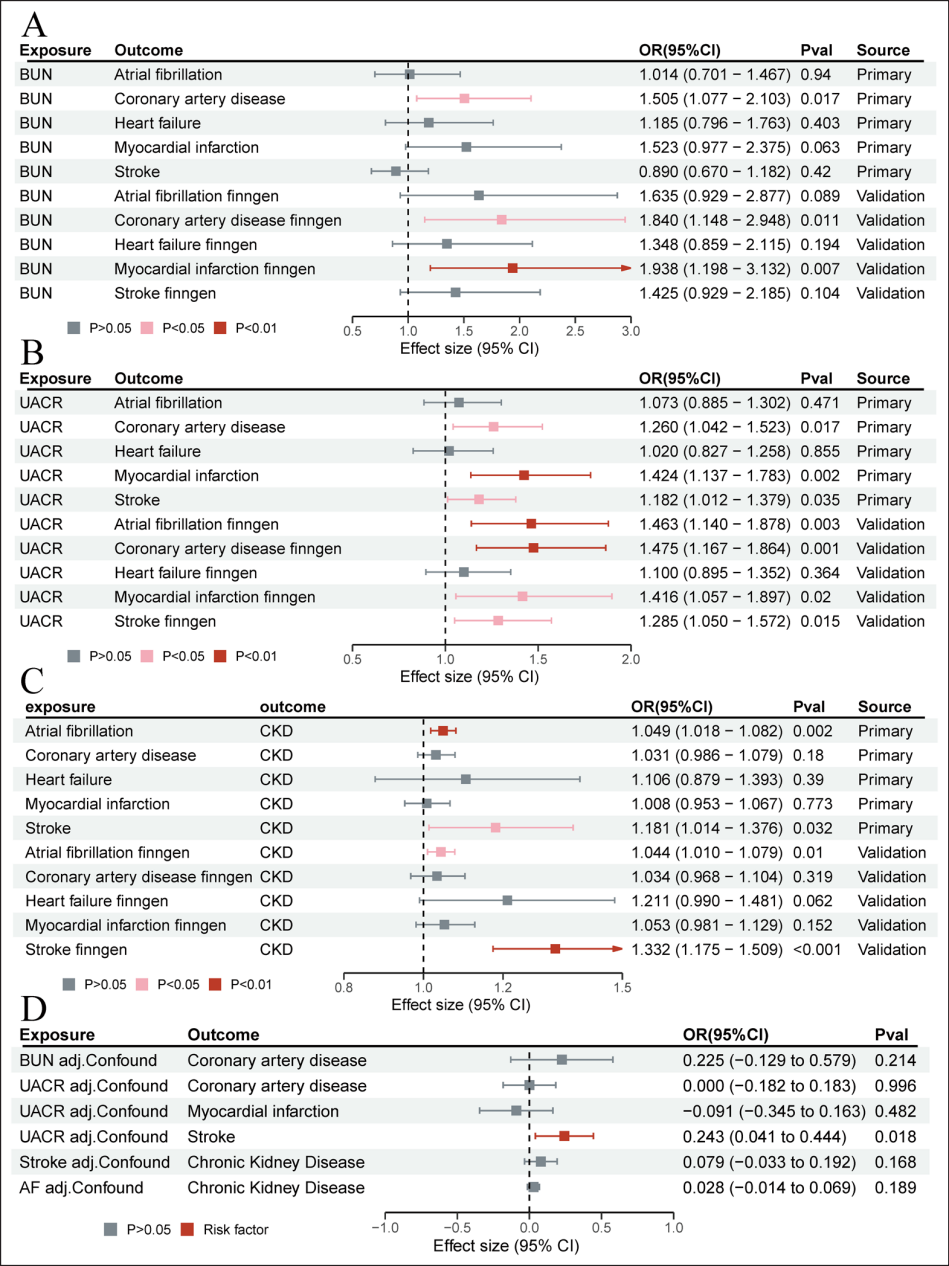
**A.** Causal effects of BUN on cardiac outcomes. **B.** Causal effects of UACR on cardiac outcomes. **C.** Causal effects of cardiac outcomes on CKD. **D.** Multivariable MR analysis of the causal effects between renal function and Cardiac Outcomes after adjusting for risk factors. AF, Atrial Fibrillation; BUN, Blood Urea Nitrogen; CKD, Chronic Kidney Disease; eGFR, Estimated Glomerular Filtration Rate; UACR, Urinary Albumin-to-Creatinine Ratio.

Moreover, an increased genetic predisposition for a higher UACR was associated with greater odds of CAD (OR 1.260; 95% CI 1.042 to 1.523; *P =* 0.017), MI (OR 1.424; 95% CI 1.137 to 1.783; *P =* 0.002), and stroke (OR 1.182; 95% CI 1.012 to 1.379; *P =* 0.035). In the five MR analysis methods, the MR-Egger method did not show statistical significance for CAD and MI results, and the direction of the OR was inconsistent with the IVW method. The relationships between UACR and CAD, MI, and stroke were validated in FinnGen (CAD: OR 1.475; 95% CI 1.167 to 1.864; *P =* 0.001; MI: OR 1.416; 95% CI 1.057 to 1.897; *P =* 0.020; Stroke: OR 1.285; 95% CI 1.050 to 1.572; *P =* 0.015) (Figures 2, 3A, 3B, and Table S3 for details).

We detected heterogeneity (*P* < 0.05), which led us to implement a random-effects model. The MR-PRESSO global test identified outliers (*P* < 0.05); however, the causal estimates retained their significance and directional consistency even after adjusting for these outliers. Furthermore, MR-Egger regression revealed no evidence of horizontal pleiotropy (*P* > 0.05) (Table S4).

No causal links were found between CKD or eGFR and the cardiovascular outcomes. Similarly, no causal associations were detected between renal function traits and the risk of AF or HF.

**Figure 4:**
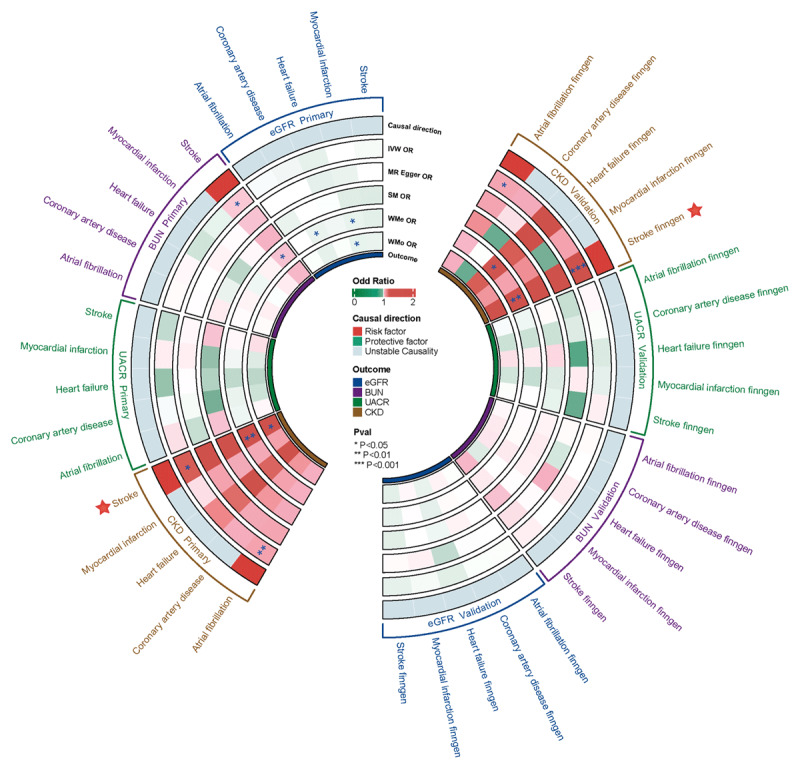
Causal effects of cardiovascular diseases on renal function using reverse MR. BUN, Blood Urea Nitrogen; CKD, Chronic Kidney Disease; eGFR, Estimated Glomerular Filtration Rate; UACR, Urinary Albumin-to-Creatinine Ratio.

In reverse-direction MR tests, the presence of stroke showed a causal relationship with increased BUN levels (OR 1.014; 95% CI 1.001 to 1.027; *P =* 0.032). However, this association was not replicated in the FinnGen cohort and became non-significant after applying the Bonferroni correction. Notably, AF (OR 1.049; 95% CI 1.018 to 1.082; *P =* 0.002) and stroke (OR 1.181; 95% CI 1.014 to 1.376; *P =* 0.032) were found to have causal impacts on elevating CKD risk, with these relationships being validated in the FinnGen cohort (details in Figure 3C, Figure 4, Figure S1, and Table S5). No reverse causal links were detected between the cardiovascular outcomes and either eGFR or UACR. Notably, the causal effects of stroke in the primary cohort and AF in the FinnGen cohort on CKD exhibited heterogeneity, but no horizontal pleiotropy was detected (Table S6).

In the multivariable MR analysis, after adjusting for cardiometabolic traits, the causal relationship between BUN levels and CAD was no longer significant. Additionally, after these adjustments, the impact of an elevated UACR on the risks of CAD and MI was also found to be non-significant. However, the causal link between increased UACR and the risk of stroke remained significant, even when considering potential cardiovascular risk factors. In a similar vein, after adjusting for cardiometabolic traits in the multivariable MR analysis, neither AF nor stroke demonstrated a significant association with an increased risk of CKD (Figure 3D and Table S7).

### Renal function and cardiac structure/function

**Figure 5:**
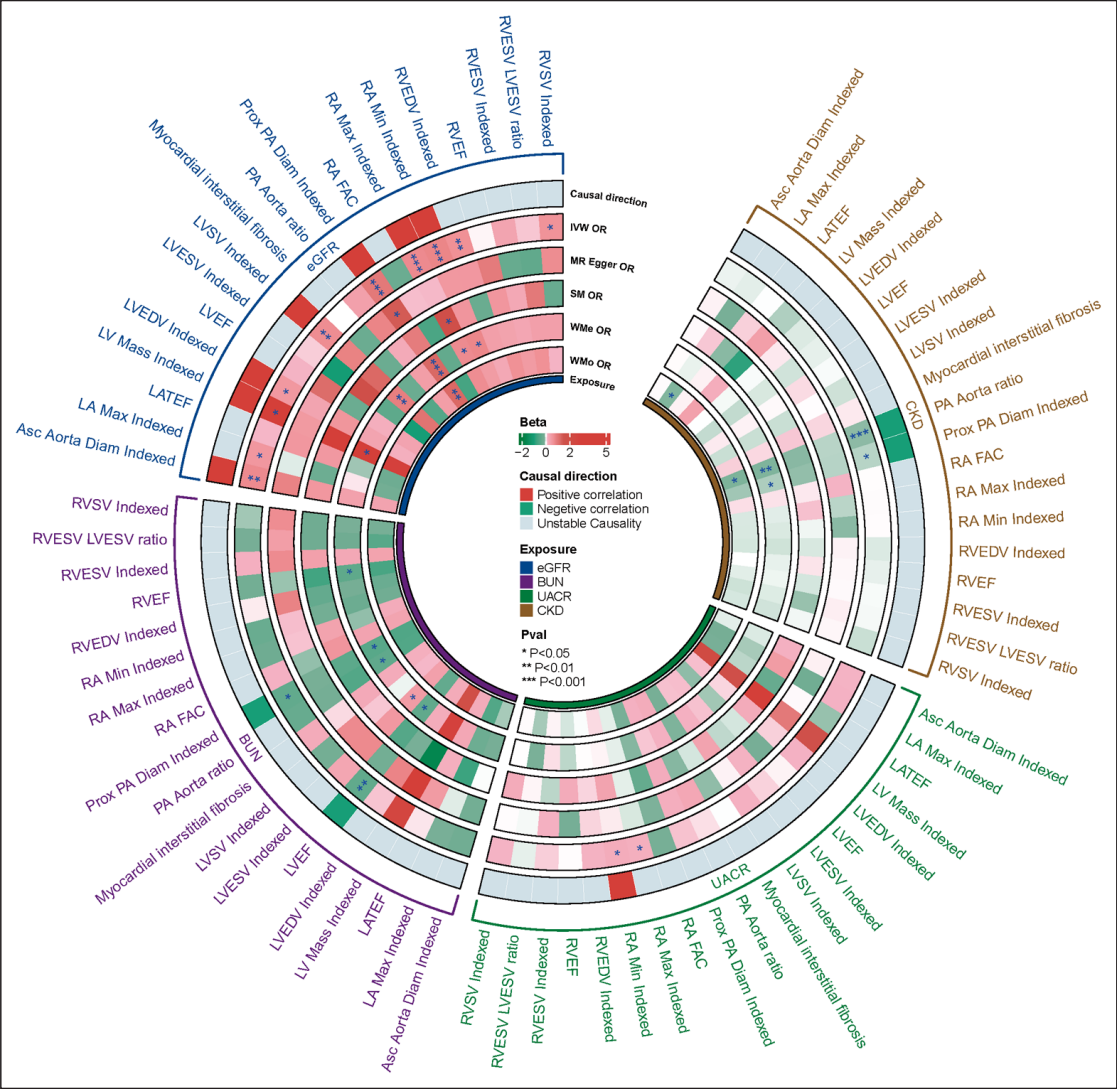
Causal effects of renal function on cardiac structure and function. Asc Aorta Diam, Ascending aorta diameter; BUN, Blood Urea Nitrogen; CKD, Chronic Kidney Disease; eGFR, Estimated Glomerular Filtration Rate; LA Max, Left atrial maximum volume; LATEF, Left atrial total ejection fraction; LVEDV, Left ventricular end diastolic volume; LVEF, Left ventricular ejection fraction; LVESV, Left ventricular end systolic volume; LV Mass, Left ventricular mass; LVSV, Left ventricular stroke volume; Prox PA Diam, Proximal pulmonary artery diameter; RA FAC, Right atrial fractional area change; RA Max, Right atrial maximum area; RA Min, Right atrial minimum area; RVEDV, Right ventricular end diastolic volume; RVEF, Right ventricular ejection fraction; RVESV, Right ventricular end systolic volume; RVSV, Right ventricular systolic volume; UACR, Urinary Albumin-to-Creatinine Ratio.

Our analysis revealed that elevated BUN levels were causally associated with a decrease in LVEF (*β* = –0.400; 95% CI –0.692 to –0.108; *P =* 0.007) and Prox PA Diam Indexed (*β* = –0.381; 95% CI –0.679 to –0.084; *P =* 0.012). Additionally, CKD showed a causal link to a reduced PA Aorta ratio (*β* = –0.086; 95% CI –0.130 to –0.042; *P =* 1.4e-4) and a decrease in Prox PA Diam Indexed (*β* = –0.053; 95% CI –0.094 to –0.012; *P =* 0.011). No causal relationships were observed between the UACR and the cardiac parameters assessed (Figure 5, Figures S2–S3, Figure S4A, and Table S8).

Crucially, a decline in the eGFR has been causally linked to reductions in a range of key cardiac parameters. These included the Asc Aorta Diam Indexed (*β* = 0.716; 95% CI 0.276 to 1.157; *P =* 0.001), Prox PA Diam Indexed (*β* = 0.961; 95% CI 0.538 to 1.385; *P =* 8.62e-6), RA Max Indexed (*β* = 0.714; 95% CI 0.312 to 1.116; *P =* 5e-4), RA Min Indexed (*β* = 0.783; 95% CI 0.351 to 1.216; *P =* 3.8e-4), LVSV Indexed (*β* = 0.676; 95% CI 0.256 to 1.096; *P =* 0.002), RVEDV Indexed (*β* = 0.625; 95% CI 0.189 to 1.061; *P =* 0.005), and RVSV Indexed (*β* = 0.593; 95% CI 0.130 to 1.055; *P =* 0.012). Our sensitivity analyses indicated the presence of heterogeneity. However, there was no evidence of horizontal pleiotropy (Table S4).

In the multivariable MR analysis, after adjusting for cardiometabolic traits, Prox PA Diam Indexed was found to have a significant causal relationship with both eGFR and CKD. Additionally, significant causal associations were also observed with eGFR for Asc Aorta Diam Indexed, LVSV Indexed, RA Max Indexed, RA Min Indexed, and RVSV Indexed (Figure S4 B and Table S7).

## Discussion

In this comprehensive MR study, we robustly establish the causal relationships within the kidney-cardiac axis, thereby integrating key findings that bridge genetic susceptibilities impacting renal function with cardiovascular disease risks.

### Renal function and Cardiac Outcomes

In our primary analysis, we found that increased levels of BUN were genetically associated with a higher risk of CAD. Similarly, elevated UACR was linked to an increased risk of CAD, MI, and stroke. These findings were consistently validated in the FinnGen cohort. However, results from the MR-Egger regression for BUN and CAD, as well as UACR and CAD/MI, lacked statistical significance, with OR directions inconsistent with those from the IVW method. This indicates that potential pleiotropy and confounding factors may have influenced our results. Consequently, interpretations of BUN’s impact on CAD and UACR’s influence on CAD/MI require cautious consideration.

The detection of causal relationships of increased BUN and UACR with greater CAD risk accords with prior observational studies demonstrating the heightened burden of atherosclerotic cardiovascular disease, including CAD, MI, and cardiovascular mortality, among individuals with CKD (12,13,14). Even after adjustment for conventional cardiovascular risk factors like hypertension and diabetes, CKD remained an independent risk factor for adverse coronary events in early reports (13,15,16).

Contrastingly, our multivariable MR analysis, after adjusting for cardiometabolic traits, revealed that the causal relationship between BUN and UACR with the risk of CAD and MI was eliminated. This suggests that the adverse effects of increased BUN and urinary albumin excretion on the likelihood of coronary disease are largely mediated through associated disturbances in blood pressure, glucose, and lipids. Therefore, focusing on lifestyle modifications and pharmaceutical treatments that target these traditional, modifiable risk factors may reduce the additional risk of CAD/MI, even in the presence of impaired renal function.

However, a key finding from our multivariable MR analysis is the sustained association between UACR and stroke, even after adjusting for cardiometabolic traits. This emphasizes UACR’s role as an independent risk factor for stroke in CKD. The association between UACR and stroke may be explained by the underlying mechanism that albuminuria is a marker of endothelial dysfunction and reflects a more generalized vascular pathology such as atherosclerosis. The above pathological changes involve the hardening and narrowing of arteries, reducing blood flow to the brain (17,18).

Emerging evidence has also unveiled albuminuria’s involvement in small-vessel cerebral disease detectable on neuroimaging (19). A higher UACR is associated with stroke, and the potential mechanisms for this association may include microvascular damage, disruption of the blood-brain barrier, and impaired cerebral perfusion (18,19). This expanded insight into albuminuria’s multifactorial impacts on cerebrovascular health could underpin its causal elevation of stroke risk independent of traditional risk factors.

The strength of albuminuria’s causal stroke risk association surviving adjustment for common risk factors highlights the need to specifically monitor UACR, even in patients without markedly reduced eGFR. Irrespective of a CKD diagnosis, UACR screening should be conducted, as the risk assessment it provides exceeds that offered by eGFR alone. Therefore, our study spotlights albuminuria as a prognostic indicator warranting close monitoring and aggressive control of accompanying vascular risk factors to mitigate cerebrovascular risk in CKD. Therapies directly targeting endothelial dysfunction and inflammatory pathways activated by albuminuria merit exploration as adjunct stroke prevention strategies.

Furthermore, our reverse-direction MR analysis initially indicated a potential causal link between stroke, AF, and an increased risk of CKD. Understanding the intricate connections between the heart, brain, and kidneys is critical. Cerebral ischemia can lead to acute kidney injury via neurohormonal pathways, which encompass sympathetic overactivity, inflammation, and oxidative stress (20). Additionally, cerebral infarcts may disrupt the central nervous system’s ability to regulate salt and water balance, negatively impacting renal function (21). These causal effects, once adjusted for cardiometabolic traits in multivariable MR analysis, are no longer significant. Therefore, meticulous control of accompanying vascular risk factors is imperative regardless of the initial organ affected to disrupt this vicious cycle and avert adverse sequelae.

### Renal function and Cardiac Remodeling

Of the cardiac parameters assessed, our multivariable analysis, even after adjusting for cardiometabolic traits, consistently showed that lower eGFR and CKD are significantly associated with a reduced index diameter of the proximal pulmonary artery. Constriction of the proximal pulmonary vasculature can reflect the presence of pulmonary arterial hypertension (PAH) and often foreshadows a dismal prognosis (22). Our findings support existing evidence that CKD is an independent risk factor for PAH, potentially through mechanisms such as endothelial dysfunction, an imbalance of vasoactive compounds, inflammation, and metabolic disorders (23,24). The pulmonary circulation is exceptionally vulnerable to these systemic effects accompanying CKD. Hence, our study suggests that declining renal function may play a causal role in predisposing individuals to pulmonary arterial hypertension and deleterious right chamber alterations.

Additionally, we found a significant correlation between reduced eGFR and decreased right heart dimensions, further highlighting eGFR’s role in cardiopulmonary damage. Our study suggests that impaired renal function may play a causal role in predisposing individuals to pulmonary arterial hypertension and deleterious right ventricular alterations. The secondary pulmonary hypertension pathophysiology in CKD patients is still complex and not completely clear. This structural remodeling of pulmonary vasculature offers clues to the underpinnings of elevated pulmonary pressures attending renal dysfunction. Future studies should focus on the genetic and epigenetic interplay between renal and cardiopulmonary pathways to identify potential therapeutic targets.

Furthermore, our study confirms a causal relationship between increased BUN levels and reduced LVEF. BUN, a key biomarker for renal excretory function, has been associated with higher rates of cardiovascular complications and mortality across multiple cohorts (25,26,27). The potential mechanisms for this relationship may involve myocardial energetic deficiencies as a result of the accumulation of nitrogenous wastes. Ammonia, a key nitrogenous waste, can cross cell membranes and disrupt tricarboxylic acid cycle enzymes at high concentrations, leading to ATP depletion (28). Given that cardiomyocytes heavily depend on mitochondrial oxidative metabolism for their high energy needs, a decrease in ATP can significantly impair contractile function (29). Despite these theoretical mechanisms and previous observational studies indicating a link, our multivariable MR analysis revealed that after adjusting for factors like lipid levels, blood pressure, and blood sugar, there is no causal association between elevated BUN and decreased LVEF.

### Limitation

Our study has several limitations. First, our participants were exclusively of European descent, limiting the applicability of our findings to other ethnicities. This underscores the need for further research to validate our results in diverse ethnic populations. Additionally, while genetic variants were selected through stringent criteria, the potential for pleiotropy and residual confounding cannot be completely excluded.

## Conclusion

Overall, this analysis deepens our understanding of the complex interplay between kidney disease and cardiovascular health, suggesting differential impacts on cardiac outcomes and structure from distinct facets of impaired renal function. It highlights avenues for reducing CAD/MI risk via modifying traditional risk factors even after kidney injury, while underscoring the need for tightened monitoring and management of cerebrovascular risk with albuminuria. Our comprehensive MR study, applying summary-level data from European ancestry cohorts, establishes causal roles of genetic susceptibility to impaired renal function in influencing cardiovascular outcomes and inducing cardiac remodeling.

## Data Accessibility Statement

Publicly available GWAS summary data were utilized for the analyses. These are available to download at cited sources.

## Additional Files

The additional files for this article can be found as follows:

10.5334/gh.1366.s1 Figure S1. Causal effects of cardiovascular diseases on renal function.

10.5334/gh.1366.s2 Figure S2. Causal effects of renal function on cardiac structure and function. **A**. Causal effects of eGFR on cardiac structure/function. **B**. Causal effects of BUN on cardiac structure/function.

10.5334/gh.1366.s3 Figure S3. Causal effects of renal function on cardiac structure and function. **A**. Causal effects of CKD on cardiac structure/function. **B**. Causal effects of UACR on cardiac structure/function.

10.5334/gh.1366.s4 Figure S4. **A**. Causal effects of renal function on cardiac structure and function. **B**. Multivariable MR analysis on renal function and cardiac structure/function after adjusting for risk factors.
BUN, Blood Urea Nitrogen; CKD, Chronic Kidney Disease; eGFR, Estimated Glomerular Filtration Rate; UACR, Urinary Albumin-to-Creatinine Ratio.

10.5334/gh.1366.s5 Table S1. Summary of GWAS data sources used in the study.

10.5334/gh.1366.s6 Table S2. Instrumental variables of renal function used for MR analysis.

10.5334/gh.1366.s7 Table S3. Effect estimates of the renal function on cardiovascular diseases.

10.5334/gh.1366.s8 Table S4. Test results for pleiotropy and heterogeneity – causal effects of the renal function on cardiovascular diseases, structure and function.

10.5334/gh.1366.s9 Table S5. Effect estimates of the cardiovascular diseases, structure and function on renal function using reverse MR.

10.5334/gh.1366.s10 Table S6. Test results for pleiotropy and heterogeneity – causal effects of the cardiovascular diseases on renal function.

10.5334/gh.1366.s11 Table S7. Multivariable MR analysis of the causal effects after adjusting for risk factors.

10.5334/gh.1366.s12 Table S8. Effect estimates of the renal function on cardiovascular magnetic resonance imaging parameters of cardiac structure and function.

## Figures and Tables

**Table:** 

AF	Atrial Fibrillation
Asc Aorta Diam	Ascending Aorta Diameter
BMI	Body Mass Index
BUN	Blood Urea Nitrogen
CAD	Coronary Artery Disease
CKD	Chronic Kidney Disease
CMR	Cardiac Magnetic Resonance
CVD	Cardiovascular Disease
DBP	Diastolic Blood Pressure
eGFR	Estimated Glomerular Filtration Rate
GWAS	Genome-Wide Association Studies
HDL	High Density Lipoprotein
HF	Heart Failure
IVs	Instrumental Variables
IVW	Inverse Variance Weighted
LA Max	Left Atrial Maximum Volume
LATEF	Left Atrial Total Ejection Fraction
LDL	Low Density Lipoprotein
LV Mass	Left Ventricular Mass
LVEDV	Left Ventricular End-Diastolic Volume
LVESV	Left Ventricular End-Systolic Volume
LVEF	Left Ventricular Ejection Fraction
LVSV	Left Ventricular Stroke Volume
MI	Myocardial Infarction
MR	Mendelian Randomization
OR	Odds Ratio
PA Aorta ratio	Pulmonary Artery-to-Aorta Diameter Ratio
Prox PA Diam	Proximal Pulmonary Artery Diameter
RA FAC	Right Atrial Fractional Area Change
RA Max	Right Atrial Maximum Area
RA Min	Right Atrial Minimum Area
RCT	Randomized Controlled Trials
RVEDV	Right Ventricular End-Diastolic Volume
RVESV	Right Ventricular End-Systolic Volume
RVESV LVESV ratio	Ratio of Right Ventricular End-Systolic to Left Ventricular End-Systolic Volume
RVEF	Right Ventricular Ejection Fraction
RVSV	Right Ventricular Stroke Volume
SBP	Systolic Blood Pressure
SM	Simple Mode
SNPs	Single Nucleotide Polymorphisms
TG	Triglycerides
UACR	Urinary Albumin-to-Creatinine Ratio
