# Supplementary figures and images for: Causal Links Between Renal Function and Cardiac Structure, Function, and Disease Risk
Source: Glob Heart. 2024 Nov 6;19(1):83. doi: 10.5334/gh.1366 (PMC11546326; doi:10.5334/gh.1366)

A

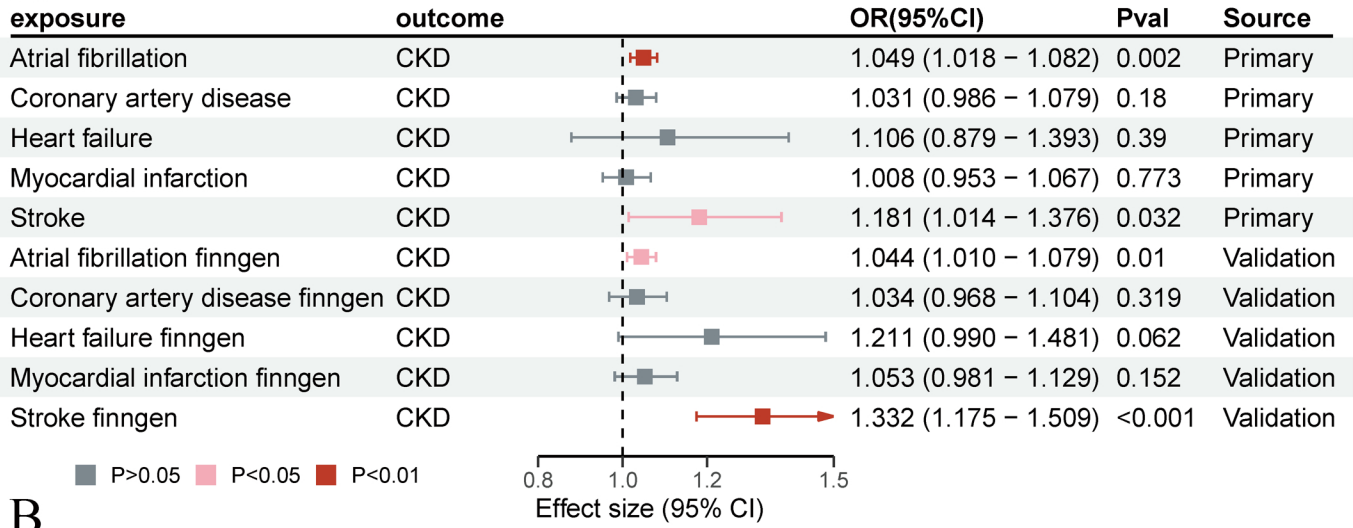

B

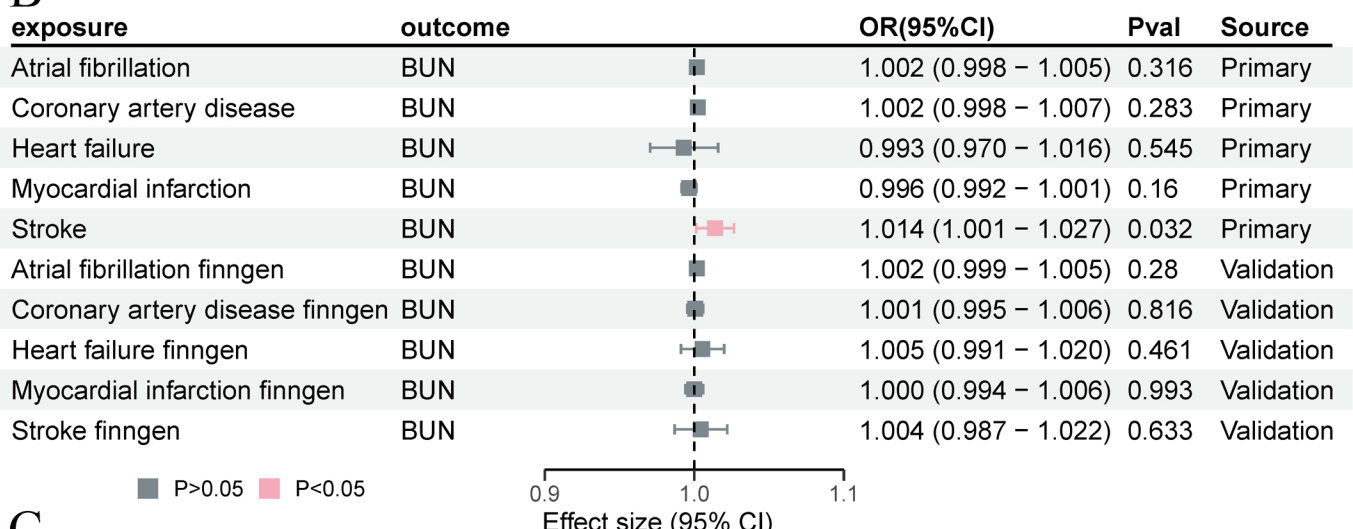

C

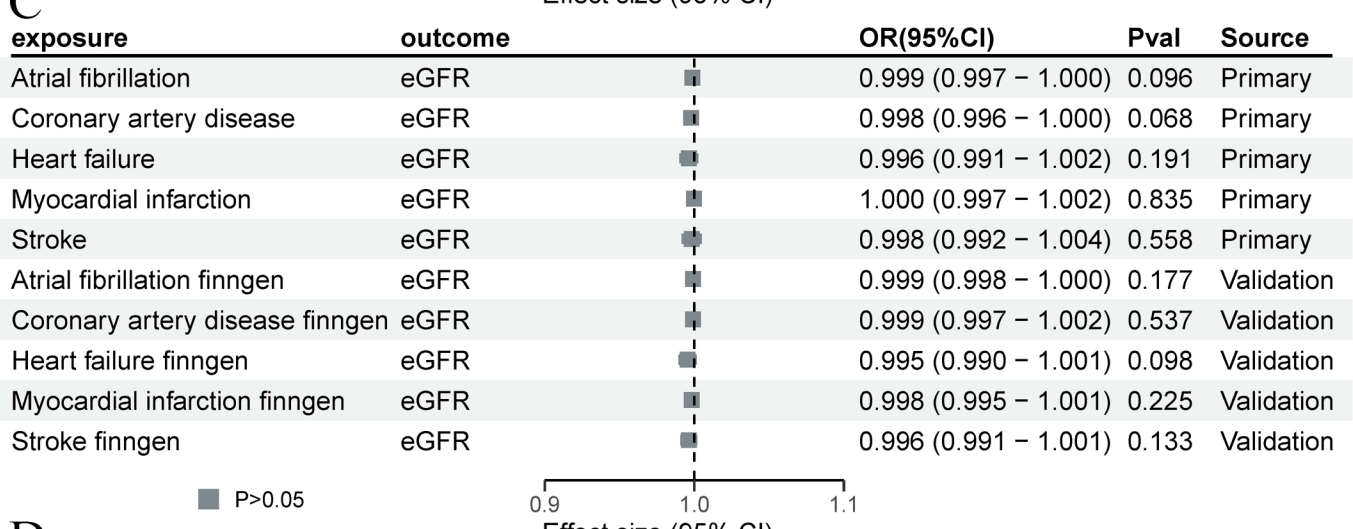

D

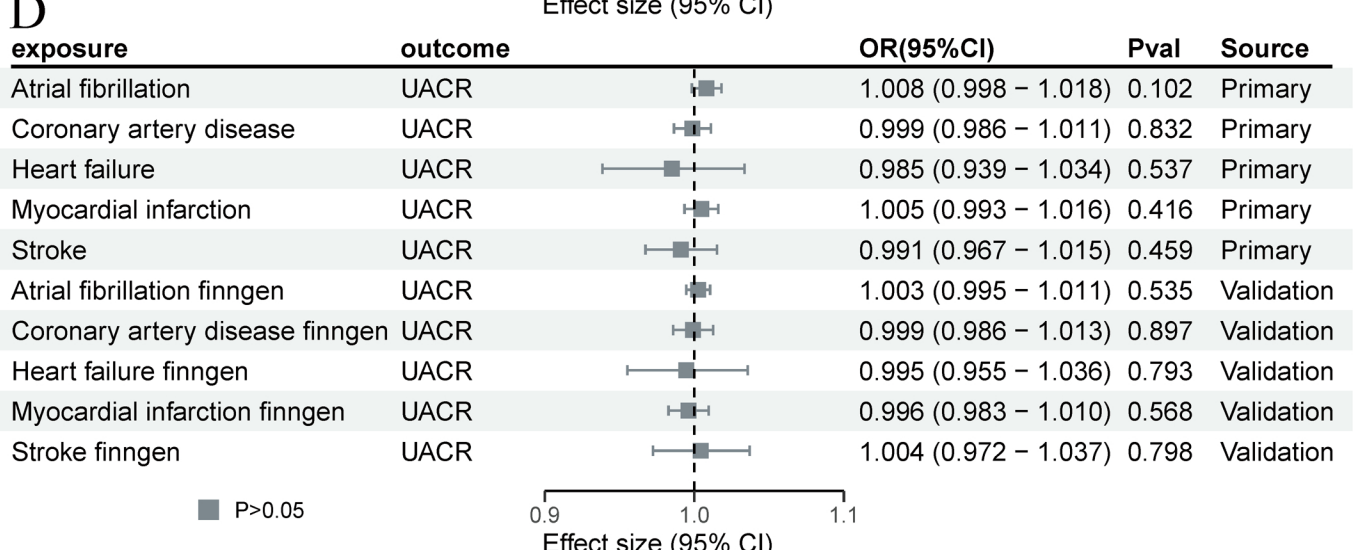

Supplement: Figure S1. — Causal effects of cardiovascular diseases on renal function. [file gh-19-1-1366-s1.pdf]

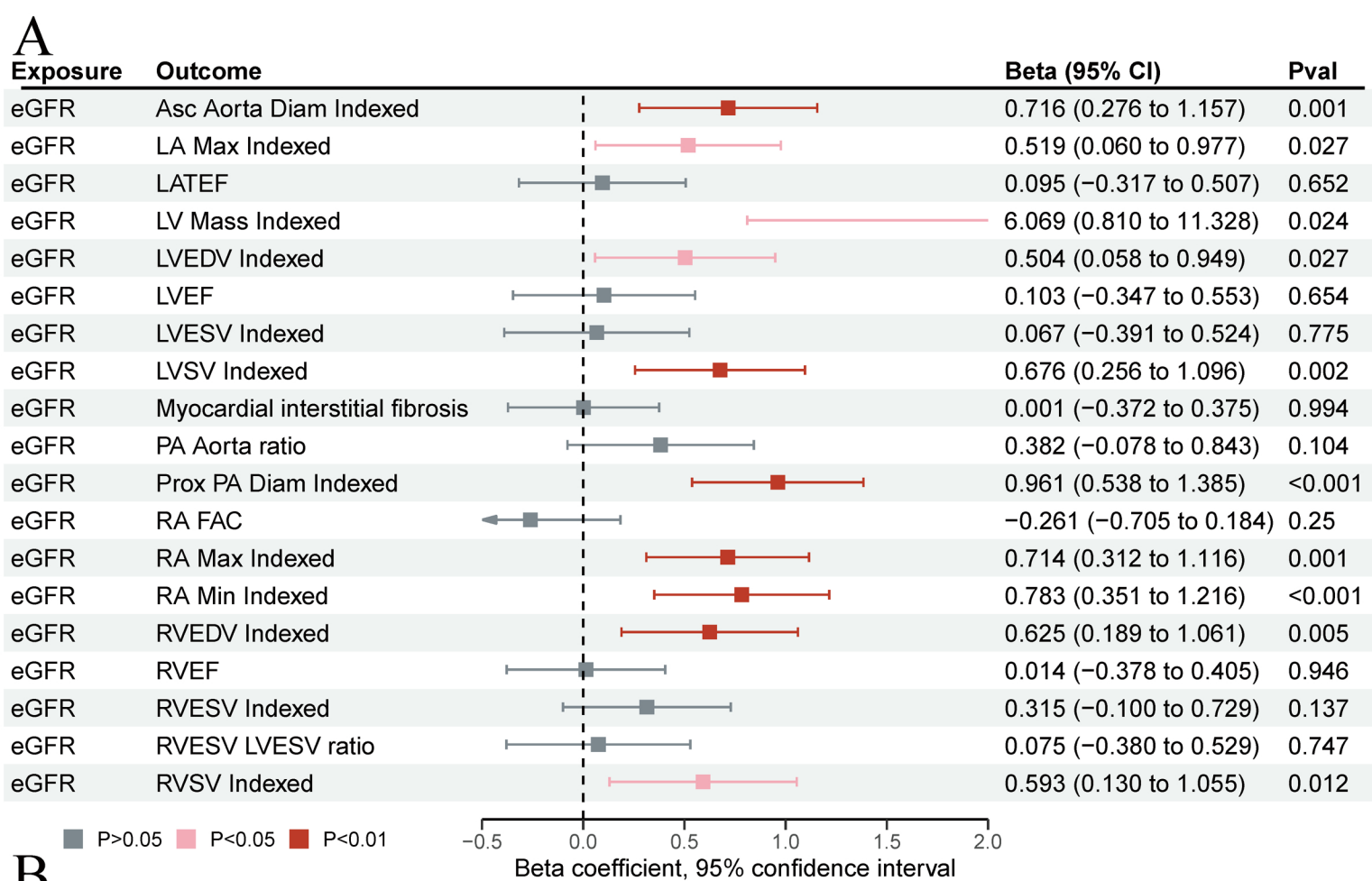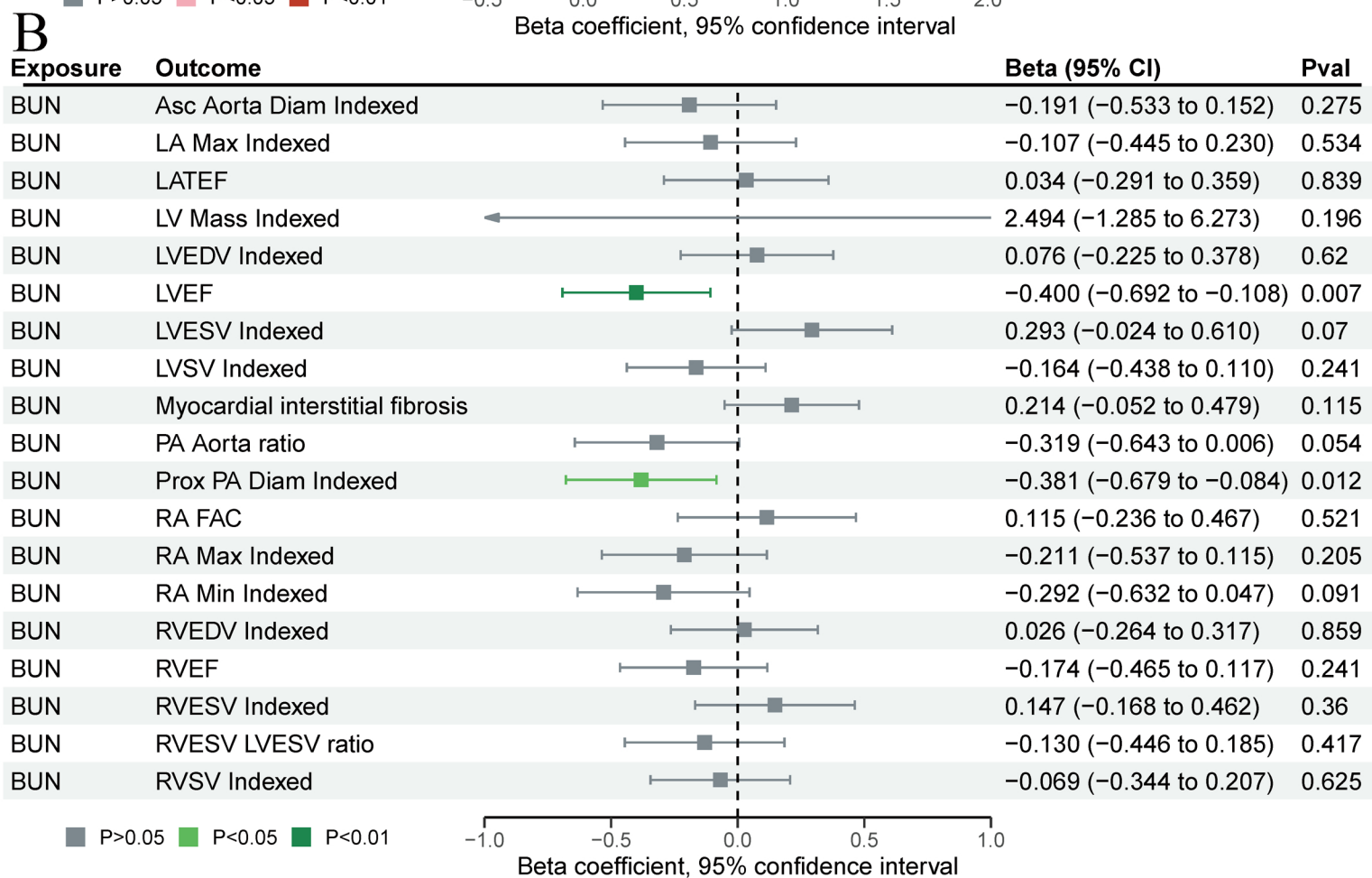

Supplement: Figure S2. — Causal effects of renal function on cardiac structure and function. A. Causal effects of eGFR on cardiac structure/function. B. Causal effects of BUN on cardiac structure/function. [file gh-19-1-1366-s2.pdf]

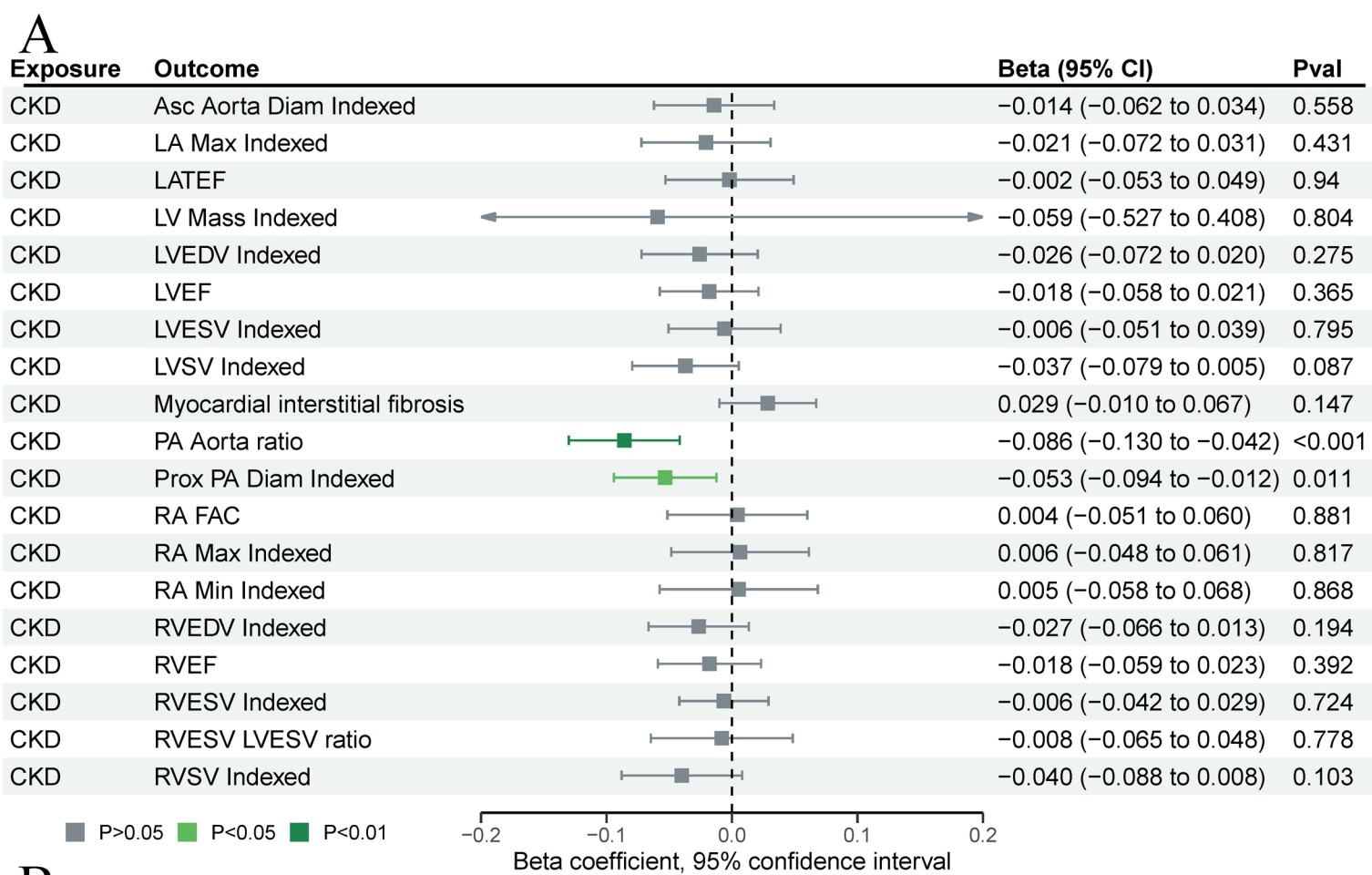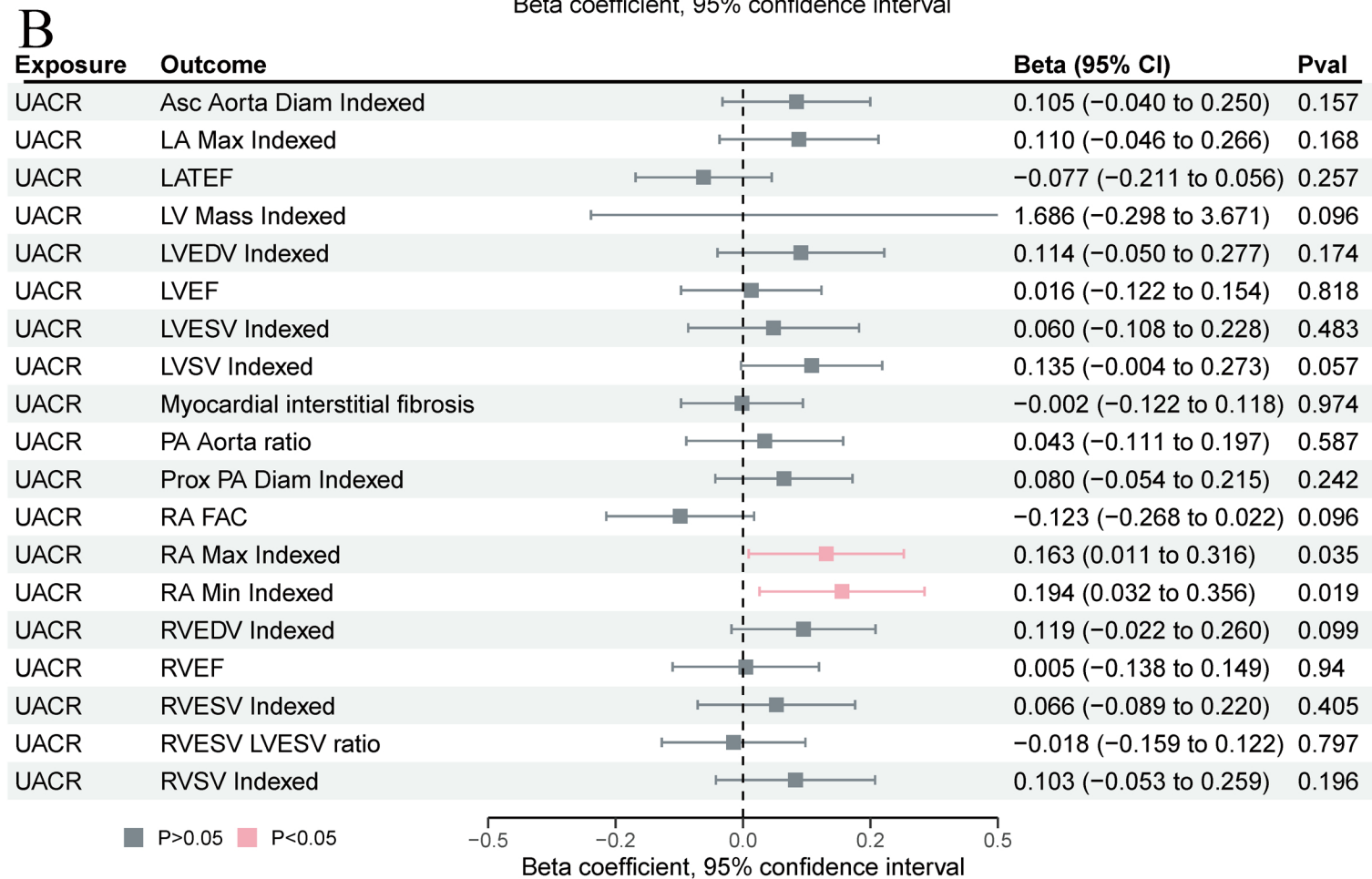

Supplement: Figure S3. — Causal effects of renal function on cardiac structure and function. A. Causal effects of CKD on cardiac structure/function. B. Causal effects of UACR on cardiac structure/function. [file gh-19-1-1366-s3.pdf]

**A**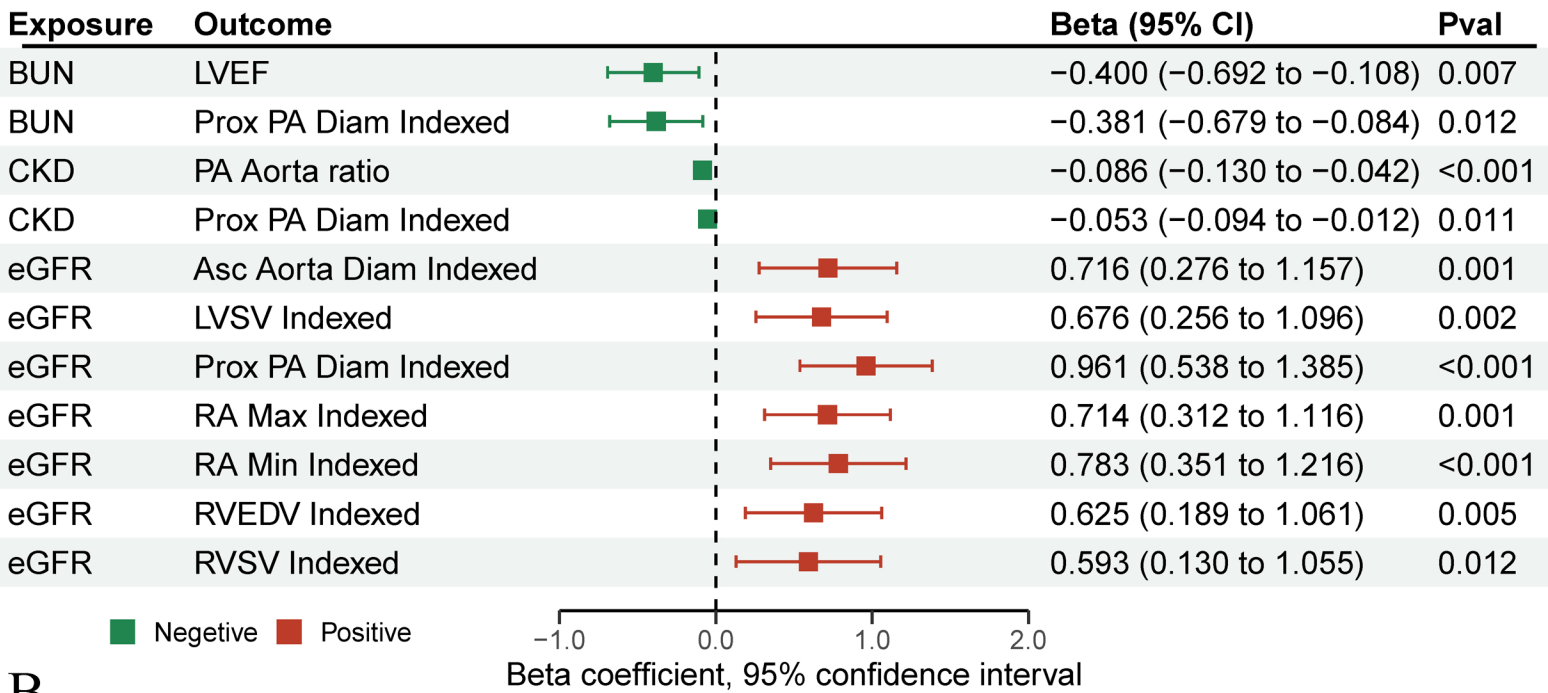**B**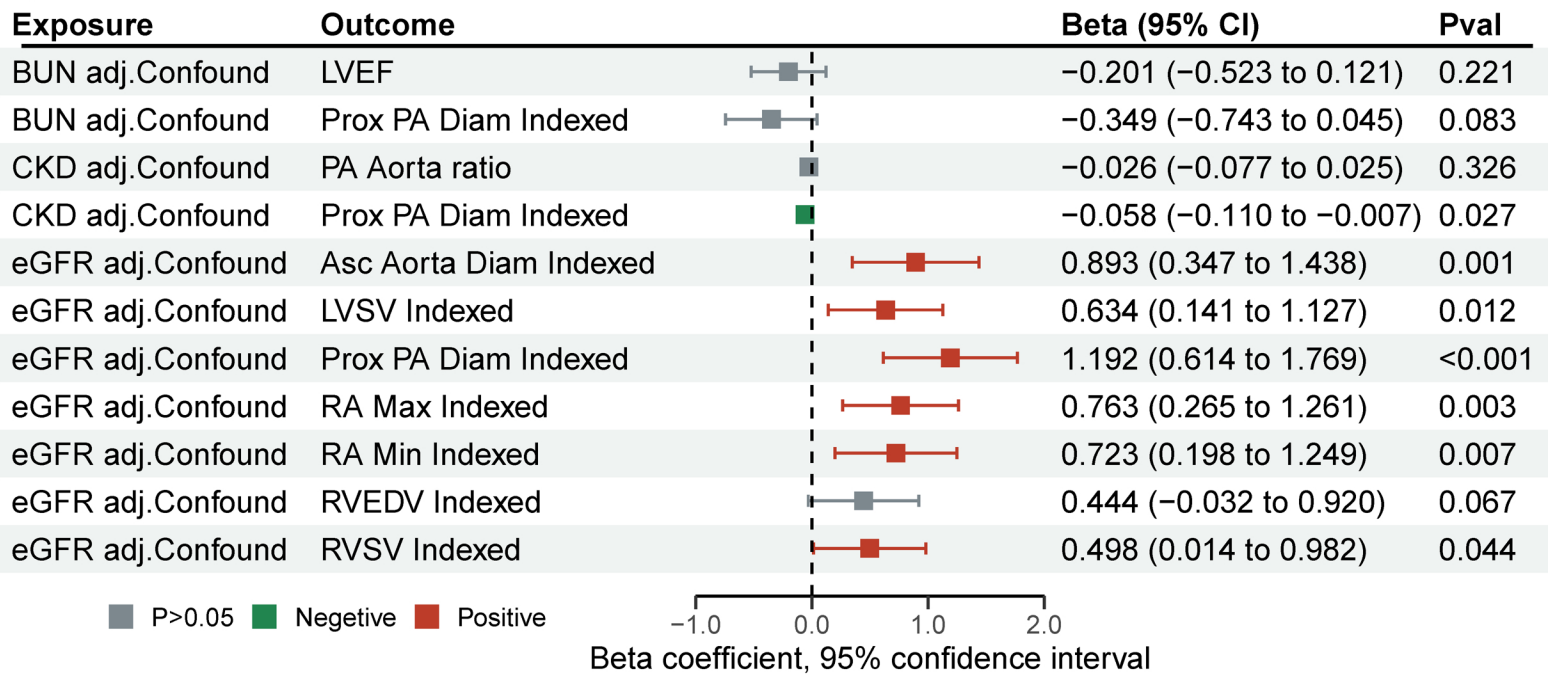

Supplement: Figure S4. — A. Causal effects of renal function on cardiac structure and function. B. Multivariable MR analysis on renal function and cardiac structure/function after adjusting for risk factors. BUN, Blood Urea Nitrogen; CKD, Chronic Kidney Disease; eGFR, Estimated Glomerular Filtration Rate; UACR, Urinary Albumin-to-Creatinine Ratio. [file gh-19-1-1366-s4.pdf]
