# Supplementary material for: Causal Links Between Renal Function and Cardiac Structure, Function, and Disease Risk
Source: Glob Heart. 2024 Nov 6;19(1):83. doi: 10.5334/gh.1366 (PMC11546326; doi:10.5334/gh.1366)
Supplement: Table S1. — Summary of GWAS data sources used in the study. [file gh-19-1-1366-s5.pdf]

Table S1. Summary of GWAS Data Sources Used in the Study.

Phenotype	Study or Biobank	Ancestry	Cases/ Controls	Disease Definition	Units	PMID
**Exposure**						
BUN	Wuttke *et al.*[1]	EUR	243 029	NA	1 SD	31152163
eGFR	Wuttke *et al.*[1]	EUR	567 460	NA	1 SD	31152163
CKD	Wuttke *et al.*[1]	EUR	64 164 / 561 055	Defined as an eGFR below 60 ml/min/1.73 m2.	Log(OR)	31152163
UACR	Teumer *et al.*[2]	EUR	547 361	NA	1 SD	31511532
**Cardiac Outcomes**						
Atrial Fibrillation	Nielsen *et al.*[3]	EUR	60 620 / 970 216	Clinically diagnosed atrial fibrillation or flutter	Log(OR)	30061737
Coronary Artery Disease	Aragam *et al.*[4]	EUR	181 522 / 984 168	documented history of angina pectoris, myocardial infarction or other forms of coronary heart disease, as well as a history of revascularization procedures	Log(OR)	36474045
Myocardial Infarction	Hartiala *et al.*[5]	EUR	61 000 / 578 000	1.Doctor-diagnosed MI; 2.ICD10 I21, I22, I23, I25.2; 3.Self-reported MI	Log(OR)	33532862
Heart Failure	Shah *et al.*[6]	EUR	47 309 / 930 014	All-cause heart failure	Log(OR)	31919418
Stroke	Mishra *et al.*[7]	EUR	73 652 / 1 234 808	any stroke	Log(OR)	36180795
**Validation Cardiac Outcomes**						
Atrial Fibrillation	FinnGen R9 data release[8]	EUR	45 766 / 191 924	ICD-10 I48	Log(OR)	NA
Heart Failure	FinnGen R9 data release[8]	EUR	26 872 / 349 361	All-cause heart failure	Log(OR)	NA
Myocardial Infarction	FinnGen R9 data release[8]	EUR	24 185 / 313 400	ICD-10 I21, I22	Log(OR)	NA
Coronary Artery Disease	FinnGen R9 data release[8]	EUR	47 550 / 313 400	ICD-10 I24, I25, T82.2, Z95.1	Log(OR)	NA

Phenotype	Study or Biobank	Ancestry	Cases/ Controls	Disease Definition	Units	PMID
Ischemic Stroke	FinnGen R9 data release[8]	EUR	25 398 / 339 920	ICD-10 I61, I63, I64	Log(OR)	NA
**Cardiac Structure And Function**						
Left Ventricular Mass	Khurshid *et al.*[9]	EUR	43 230	NA	1 SD	36944631
Left Ventricle<br>Aorta<br>Pulmonary Artery<br>And Right Heart	Pirruccello *et al.*[10]	EUR	45 504	NA	1 SD	35697867
Myocardial Interstitial Fibrosis	Nauffal *et al.*[11]	EUR	41 505	NA	1 SD	37081215
Left Atrial	Ahlberg *et al.*[12]	EUR	35 658	NA	1 SD	34338756
**Risk Factors**						
HDL Cholesterol	UK Biobank[13]	EUR	432 009	NA	1 SD	NA
LDL Cholesterol	UK Biobank[13]	EUR	469 869	NA	1 SD	NA
Triglycerides	UK Biobank[13]	EUR	470 337	NA	1 SD	NA
Apolipoprotein A1	UK Biobank[13]	EUR	429 666	NA	1 SD	NA
Apolipoprotein B	UK Biobank[13]	EUR	468 375	NA	1 SD	NA
Systolic Blood Pressure	UK Biobank[13]	EUR	475 939	NA	1 mmHg	NA
Diastolic Blood Pressure	UK Biobank[13]	EUR	475 944	NA	1 mmHg	NA
Body Mass Index	Pulit *et al.*[14]	EUR	694 649	NA	1 SD	30239722

Reference:

1 Wuttke M, Li Y, Li M, et al. A catalog of genetic loci associated with kidney function from analyses of a million individuals. Nat Genet. 2019;51(6):957-972.

2 Teumer A, Li Y, Ghasemi S, et al. Genome-wide association meta-analyses and fine-mapping elucidate pathways influencing albuminuria. *Nat Commun.* 2019;10(1):4130.

3 Nielsen JB, Thorolfsdottir RB, Fritsche LG, et al. Biobank-driven genomic discovery yields new insight into atrial fibrillation biology. *Nat Genet.* 2018;50(9):1234-1239.

4 Aragam KG, Jiang T, Goel A, et al. Discovery and systematic characterization of risk variants and genes for coronary artery disease in over a million participants. *Nat Genet.* 2022;54(12):1803-1815.

5 Hartiala JA, Han Y, Jia Q, et al. Genome-wide analysis identifies novel susceptibility loci for

myocardial infarction. *Eur Heart J.* 2021;42(9):919-933.

6 Shah S, Henry A, Roselli C, et al. Genome-wide association and Mendelian randomisation analysis provide insights into the pathogenesis of heart failure. *Nat Commun.* 2020;11(1):163.

7 Mishra A, Malik R, Hachiya T, et al. Stroke genetics informs drug discovery and risk prediction across ancestries. *Nature.* 2022;611(7934):115-123.

8 Kurki MI, Karjalainen J, Palta P, et al. FinnGen provides genetic insights from a well-phenotyped isolated population. *Nature.* 2023;613(7944):508-518.

9 Khurshid S, Lazarte J, Pirruccello JP, et al. Clinical and genetic associations of deep learning-derived cardiac magnetic resonance-based left ventricular mass. *Nat Commun.* 2023;14(1):1558.

10 Pirruccello JP, Di Achille P, Nauffal V, et al. Genetic analysis of right heart structure and function in 40,000 people. *Nat Genet.* 2022;54(6):792-803.

11 Nauffal V, Di Achille P, Klarqvist M, et al. Genetics of myocardial interstitial fibrosis in the human heart and association with disease. *Nat Genet.* 2023;55(5):777-786.

12 Ahlberg G, Andreasen L, Ghouse J, et al. Genome-wide association study identifies 18 novel loci associated with left atrial volume and function. *Eur Heart J.* 2021;42(44):4523-4534.

13 Jiang L, Zheng Z, Fang H, Yang J. A generalized linear mixed model association tool for biobank-scale data. *Nat Genet.* 2021;53(11):1616-1621.

14 Pulit SL, Stoneman C, Morris AP, et al. Meta-analysis of genome-wide association studies for body fat distribution in 694 649 individuals of European ancestry. *Hum Mol Genet.* 2019;28(1):166-174.
