# Supplementary material for: Causal Links Between Renal Function and Cardiac Structure, Function, and Disease Risk
Source: Glob Heart. 2024 Nov 6;19(1):83. doi: 10.5334/gh.1366 (PMC11546326; doi:10.5334/gh.1366)
Supplement: Table S2. — Instrumental variables of renal function used for MR analysis. [file gh-19-1-1366-s6.pdf]

Table S2. Instrumental variables of renal function used f

Exposure	SNP	chr	pos	Effect allele	Other allele	eaf	samplesize
BUN	rs77637678	1	51026402	A	G	0.035	242058
BUN	rs2755253	1	67470843	T	C	0.71	242935
BUN	rs10874312	1	82944571	A	G	0.66	238602
BUN	rs34277475	1	205416664	A	C	0.2	160491
BUN	rs3791760	2	10118424	T	C	0.75	242909
BUN	rs780094	2	27741237	T	C	0.39	238602
BUN	rs11123169	2	113967075	T	C	0.67	240985
BUN	rs34773350	2	121985759	T	C	0.14	242742
BUN	rs1047891	2	211540507	A	C	0.31	199912
BUN	rs6709815	2	219675067	T	G	0.51	242080
BUN	rs1609783	3	25095911	A	G	0.53	200863
BUN	rs998394	3	64801187	A	G	0.46	238602
BUN	rs9849724	3	121626904	T	G	0.54	242999
BUN	rs28631273	3	135934711	A	G	0.28	242960
BUN	rs16853703	3	169144963	T	C	0.86	242912
BUN	rs6789987	3	187763398	T	C	0.27	200863
BUN	rs13127170	4	3758382	A	G	0.45	169748
BUN	rs10019833	4	77357592	T	C	0.59	191428
BUN	rs6880621	5	34504382	A	G	0.52	242873
BUN	rs1842076	5	40237018	T	C	0.72	241842
BUN	rs11744018	5	72436224	A	G	0.31	241313
BUN	rs4976646	5	176788570	T	C	0.66	242080
BUN	rs283562	6	51061395	T	G	0.43	242946
BUN	rs10457487	6	127519234	A	C	0.48	239697
BUN	rs300143	6	166421127	A	G	0.57	199912
BUN	rs13230625	7	1286244	A	G	0.7	152092
BUN	rs55914958	7	101237753	T	C	0.33	242663
BUN	rs73728279	7	151411494	T	G	0.28	200863
BUN	rs73555305	8	23721420	T	C	0.073	242887
BUN	rs56411466	8	30280065	A	G	0.48	200863
BUN	rs11989898	8	127489030	A	G	0.85	238700
BUN	rs635634	9	136155000	T	C	0.19	241511
BUN	rs35668759	10	94840631	A	C	0.84	200863
BUN	rs6597862	10	126649723	A	C	0.24	242944
BUN	rs3925584	11	30760335	T	C	0.55	240383
BUN	rs11039216	11	47406592	T	C	0.52	200863
BUN	rs642803	11	65560620	T	C	0.46	242080
BUN	rs4980786	11	69239611	T	C	0.57	242080
BUN	rs4567493	11	86634423	A	G	0.36	242745
BUN	rs1551210	12	42865874	T	C	0.4	242812
BUN	rs73114872	12	56861034	T	C	0.82	243007
BUN	rs7974833	12	57791833	T	C	0.76	229632
BUN	rs2264750	12	121450165	T	C	0.3	242945
BUN	rs9572784	13	72341727	T	C	0.38	242080

BUN	rs3911726	15	53964746	A	G	0.49	240888
BUN	rs4886755	15	76298132	A	G	0.49	241764
BUN	rs12908437	15	99287375	T	C	0.37	242080
BUN	rs77924615	16	20392332	A	G	0.2	199912
BUN	rs9926289	16	53820503	A	G	0.41	241888
BUN	rs2229295	17	36047276	T	G	0.17	226805
BUN	rs4968247	17	44988703	T	C	0.67	240985
BUN	rs12940987	17	59269257	A	G	0.77	241724
BUN	rs28680305	17	65579728	A	G	0.35	175621
BUN	rs6416861	17	80138056	T	C	0.74	242873
BUN	rs60764613	18	1839911	T	G	0.15	242779
BUN	rs269977	18	42782202	A	G	0.52	242925
BUN	rs1484873	18	43206985	A	G	0.06	239697
BUN	rs41301139	18	43262408	A	G	0.018	213033
BUN	rs8106700	19	7200558	A	G	0.27	242974
BUN	rs2238691	19	46179043	A	G	0.21	241435
BUN	rs838144	19	49250239	T	C	0.52	241758
BUN	rs56038390	21	16582710	A	G	0.67	199912
BUN	rs219781	21	37832621	T	G	0.25	239697
eGFR	rs6667182	1	15914545	T	C	0.32	487087
eGFR	rs11260709	1	16557691	T	C	0.68	525153
eGFR	rs11261022	1	18807953	A	C	0.36	562779
eGFR	rs78614739	1	27174180	T	C	0.17	567440
eGFR	rs499600	1	46039077	T	G	0.15	566619
eGFR	rs2792796	1	56715908	T	C	0.61	567327
eGFR	rs407102	1	109846278	T	C	0.7	561828
eGFR	rs267738	1	150940625	T	G	0.79	558035
eGFR	rs3845534	1	163738950	A	G	0.49	561828
eGFR	rs4656220	1	170649277	T	C	0.37	566468
eGFR	rs3795503	1	180905694	T	C	0.33	522703
eGFR	rs78444298	1	184672098	A	G	0.019	539647
eGFR	rs3850625	1	201016296	A	G	0.12	540112
eGFR	rs78986840	1	208051123	T	C	0.94	566126
eGFR	rs7514450	1	220991171	T	C	0.43	567369
eGFR	rs2490391	1	243469669	A	C	0.46	561667
eGFR	rs3791221	2	226933	A	G	0.65	559185
eGFR	rs1595810	2	12115479	A	G	0.2	567140
eGFR	rs807624	2	15782471	T	G	0.34	558234
eGFR	rs4567937	2	18676265	A	G	0.32	567378
eGFR	rs780094	2	27741237	T	C	0.38	557127
eGFR	rs168505	2	54920968	T	C	0.4	567065
eGFR	rs6546869	2	73895765	A	G	0.22	567325
eGFR	rs72995641	2	103166325	A	G	0.2	567338
eGFR	rs140179699	2	120936492	A	G	0.95	496409
eGFR	rs11694902	2	121988884	A	G	0.14	566391
eGFR	rs12989250	2	148776438	A	G	0.31	525153
eGFR	rs7565830	2	159810691	A	G	0.72	561828
eGFR	rs35472707	2	169995581	T	C	0.05	524202

eGFR	rs1819008	2	177697882	T	C	0.55	567242
eGFR	rs1047891	2	211540507	A	C	0.31	524202
eGFR	rs1548945	2	217665788	T	C	0.41	522703
eGFR	rs3731906	2	220346596	T	C	0.67	551622
eGFR	rs7592697	2	230665303	T	C	0.65	525153
eGFR	rs9838792	3	38546726	A	G	0.39	567236
eGFR	rs67216675	3	49493151	T	G	0.32	567437
eGFR	rs35004449	3	52852897	T	G	0.27	567447
eGFR	rs66473811	3	64000464	T	C	0.84	525153
eGFR	rs9868185	3	121657593	A	G	0.54	567460
eGFR	rs3905668	3	135931586	A	G	0.72	567415
eGFR	rs1397764	3	141750810	A	G	0.28	567344
eGFR	rs112545201	3	185803532	T	C	0.13	564927
eGFR	rs363092	4	3196029	A	C	0.42	557127
eGFR	rs7667050	4	23813109	T	C	0.47	567288
eGFR	rs1910738	4	52687939	T	G	0.71	519536
eGFR	rs13146355	4	77412140	A	G	0.44	561828
eGFR	rs1458038	4	81164723	T	C	0.3	558234
eGFR	rs223308	4	103812499	A	G	0.52	567454
eGFR	rs6555317	5	498235	A	G	0.69	479112
eGFR	rs13157326	5	34504277	A	G	0.48	528332
eGFR	rs11951093	5	39421736	A	G	0.42	524202
eGFR	rs7719960	5	52809118	A	G	0.23	525153
eGFR	rs79760705	5	53298716	T	G	0.11	566470
eGFR	rs55938024	5	67742038	A	G	0.12	529355
eGFR	rs78660602	5	68043894	A	G	0.9	567458
eGFR	rs3797537	5	78322650	A	G	0.71	567434
eGFR	rs27879	5	131432689	A	C	0.6	566658
eGFR	rs115926813	5	131790662	A	G	0.04	566822
eGFR	rs12163971	5	132226669	A	C	0.16	567456
eGFR	rs3812036	5	176813404	T	C	0.26	525153
eGFR	rs62394289	6	25848911	A	G	0.12	566867
eGFR	rs3993747	6	31580507	A	G	0.64	559387
eGFR	rs3134605	6	32159956	T	C	0.8	532674
eGFR	rs10498755	6	43330756	T	C	0.083	566438
eGFR	rs881858	6	43806609	A	G	0.7	561519
eGFR	rs6458868	6	52630153	T	C	0.65	567401
eGFR	rs1268176	6	109018046	A	G	0.34	567368
eGFR	rs9375702	6	130384187	T	C	0.69	567096
eGFR	rs2608915	6	131871314	A	G	0.78	524202
eGFR	rs3822939	6	133849789	A	G	0.46	567228
eGFR	rs62432759	6	154858365	A	G	0.78	525153
eGFR	rs11753995	6	160575366	A	G	0.17	561828
eGFR	rs4410790	7	17284577	T	C	0.37	559629
eGFR	rs6948759	7	33095688	T	C	0.21	567243
eGFR	rs73116829	7	50739738	A	G	0.11	567452
eGFR	rs35072105	7	65609817	A	G	0.55	566397
eGFR	rs55759218	7	77453357	A	G	0.27	567446

eGFR	rs325442	7	127457228	A	G	0.4	567256
eGFR	rs3757387	7	128576086	T	C	0.55	525153
eGFR	rs62491533	7	129564134	T	C	0.83	567402
eGFR	rs10224002	7	151415041	A	G	0.72	547335
eGFR	rs6971211	7	155664686	T	C	0.41	558234
eGFR	rs2365286	7	156258179	A	G	0.74	566450
eGFR	rs11784052	8	8671962	T	C	0.46	564609
eGFR	rs1913641	8	76483239	T	G	0.48	567381
eGFR	rs4566	8	86361082	T	G	0.61	561828
eGFR	rs10086569	8	87247209	T	C	0.24	566459
eGFR	rs79346194	8	120886486	A	G	0.69	525153
eGFR	rs2954017	8	126476873	T	C	0.46	487087
eGFR	rs10964603	9	20559727	T	C	0.78	524202
eGFR	rs544169	9	33956791	A	G	0.74	567460
eGFR	rs72714330	9	71102246	T	C	0.12	544847
eGFR	rs2039424	9	71432174	A	G	0.62	553427
eGFR	rs4836732	9	119266695	T	C	0.53	558234
eGFR	rs11794652	9	133496402	A	G	0.16	515827
eGFR	rs10122824	9	139109861	T	G	0.34	536237
eGFR	rs3793805	10	51049027	A	G	0.57	562762
eGFR	rs10994860	10	52645424	T	C	0.19	561828
eGFR	rs7084764	10	69960430	A	G	0.5	567294
eGFR	rs10887903	10	82210641	A	G	0.53	566345
eGFR	rs2068888	10	94839642	A	G	0.45	558234
eGFR	rs9419939	10	104547610	A	G	0.2	525153
eGFR	rs10430743	10	126456997	T	G	0.43	567178
eGFR	rs4072824	11	2187439	A	C	0.67	497458
eGFR	rs233438	11	2794392	A	G	0.81	566347
eGFR	rs396341	11	5571897	T	C	0.26	567440
eGFR	rs3925584	11	30760335	T	C	0.55	560131
eGFR	rs10838702	11	47410888	T	G	0.38	565704
eGFR	rs929934	11	57411252	T	C	0.43	525153
eGFR	rs11227260	11	65461158	T	G	0.35	567453
eGFR	rs3018667	11	68912221	A	G	0.32	567127
eGFR	rs2509851	11	118966780	A	C	0.63	567339
eGFR	rs2156664	11	121645005	T	C	0.27	567384
eGFR	rs11062167	12	364739	A	G	0.53	567083
eGFR	rs34117451	12	3232674	T	C	0.17	567397
eGFR	rs16930370	12	3387697	T	C	0.82	561828
eGFR	rs117113238	12	12209203	A	G	0.095	524202
eGFR	rs10846157	12	15325031	A	C	0.81	567443
eGFR	rs2634675	12	48740855	A	G	0.46	528215
eGFR	rs61927768	12	50898728	A	G	0.29	525153
eGFR	rs7974833	12	57791833	T	C	0.76	554062
eGFR	rs17696736	12	112486818	A	G	0.57	556704
eGFR	rs41284816	13	50655989	T	G	0.026	520862
eGFR	rs7326821	13	96068204	A	G	0.83	525153
eGFR	rs2071047	14	54418411	A	G	0.41	565402

eGFR	rs1569011	14	81853291	A	G	0.44	561828
eGFR	rs3814828	14	93406702	A	G	0.38	519421
eGFR	rs11856829	15	39277781	T	C	0.49	524202
eGFR	rs2412608	15	41496713	T	C	0.49	525153
eGFR	rs1585499	15	54009154	T	C	0.45	524202
eGFR	rs1994887	15	57793765	A	C	0.28	561828
eGFR	rs11071738	15	63580155	T	C	0.53	567124
eGFR	rs8028182	15	75718669	T	G	0.19	514820
eGFR	rs10851885	15	76304503	A	G	0.76	558234
eGFR	rs113956264	16	1997004	T	C	0.036	432341
eGFR	rs8050794	16	3743046	T	C	0.29	518790
eGFR	rs77924615	16	20392332	A	G	0.2	524202
eGFR	rs7188071	16	28917644	T	C	0.36	567233
eGFR	rs12920176	16	51761084	A	C	0.59	525153
eGFR	rs7203398	16	53189672	A	C	0.73	561828
eGFR	rs7185391	16	68323115	T	G	0.29	525153
eGFR	rs4788809	16	71615820	A	G	0.37	567442
eGFR	rs11644400	16	79928186	T	C	0.85	556096
eGFR	rs9894634	17	1967501	T	C	0.6	566261
eGFR	rs3744139	17	17045733	T	G	0.66	563866
eGFR	rs2252281	17	19437187	T	C	0.61	561841
eGFR	rs4794814	17	37696852	A	G	0.75	561828
eGFR	rs67571561	17	37920847	T	C	0.96	567040
eGFR	rs12940987	17	59269257	A	G	0.77	566151
eGFR	rs11657044	17	59450105	T	C	0.17	567121
eGFR	rs1719934	18	5585158	A	G	0.54	567179
eGFR	rs9807656	18	42346956	T	C	0.9	566393
eGFR	rs1377164	18	59328934	T	C	0.21	567386
eGFR	rs3111316	19	13038415	A	G	0.59	561991
eGFR	rs4808154	19	18843752	T	C	0.71	477618
eGFR	rs8101667	19	33402419	T	C	0.33	567448
eGFR	rs1643471	19	38456353	T	C	0.54	525153
eGFR	rs281380	19	49214470	T	C	0.63	516594
eGFR	rs6135224	20	14677650	A	G	0.69	567430
eGFR	rs6088528	20	33156742	A	G	0.5	566659
eGFR	rs6029632	20	39960342	A	G	0.4	566020
eGFR	rs736820	20	43034016	A	G	0.37	523248
eGFR	rs623834	20	60927538	T	C	0.58	521382
eGFR	rs2145166	20	62149840	A	G	0.16	417021
eGFR	rs2823139	21	16576783	A	G	0.34	558271
eGFR	rs13047277	21	16794774	T	C	0.72	522703
eGFR	rs2834321	21	35358734	A	G	0.18	567459
eGFR	rs2244237	21	37818141	T	G	0.22	567404
eGFR	rs2074204	22	30403996	T	C	0.26	561828
eGFR	rs132641	22	36544729	A	G	0.16	561549
eGFR	rs112880707	22	40884662	T	C	0.11	519421
eGFR	rs1883991	22	43112818	A	C	0.69	560763
UACR	rs17035646	1	10796547	A	G	0.34	545747

UACR	rs4641276	1	33760743	T	C	0.246	546427
UACR	rs1337526	1	47965130	A	G	0.198	547293
UACR	rs34257409	1	155131394	T	G	0.404	547303
UACR	rs16864515	1	171435542	A	C	0.096	547278
UACR	rs78444298	1	184672098	A	G	0.019	526702
UACR	rs3850625	1	201016296	A	G	0.119	529461
UACR	rs4665972	2	27598097	T	C	0.395	533701
UACR	rs2880119	2	111809330	A	C	0.858	547288
UACR	rs1047891	2	211540507	A	C	0.315	532767
UACR	rs7597336	2	227942519	A	G	0.873	547352
UACR	rs73065147	3	46894939	T	C	0.931	547351
UACR	rs11709284	3	52559705	A	G	0.559	547345
UACR	rs112607182	3	170027407	T	C	0.075	504999
UACR	rs13132085	4	56460085	A	G	0.289	546427
UACR	rs10023335	4	77358987	T	C	0.593	547356
UACR	rs6535594	4	149132756	A	G	0.498	546427
UACR	rs76027714	5	53275370	A	G	0.924	533701
UACR	rs1309546	5	64290004	T	C	0.551	547310
UACR	rs162890	5	131623658	T	C	0.332	533701
UACR	rs56336142	6	39134099	T	C	0.788	547253
UACR	rs4410790	7	17284577	T	C	0.369	546427
UACR	rs17158386	7	29805361	A	G	0.258	532767
UACR	rs35692677	7	69902654	A	G	0.186	533701
UACR	rs1057868	7	75615006	T	C	0.285	547361
UACR	rs7812843	8	23737080	A	G	0.501	547324
UACR	rs4738817	8	61620613	A	G	0.454	546427
UACR	rs6998967	8	81364205	A	G	0.166	546427
UACR	rs2954021	8	126482077	A	G	0.492	547342
UACR	rs45551835	10	16932384	A	G	0.015	541702
UACR	rs1801234	10	16979661	T	C	0.574	546350
UACR	rs562661763	10	17160005	T	G	0.993	489376
UACR	rs67339103	10	77893686	A	G	0.216	533701
UACR	rs2068888	10	94839642	A	G	0.452	546427
UACR	rs988712	11	27563382	T	G	0.236	545477
UACR	rs7115200	11	71752160	T	G	0.56	529571
UACR	rs12790943	11	120058623	T	C	0.422	547291
UACR	rs2601006	12	69979517	T	C	0.343	547241
UACR	rs4899263	14	69285264	A	G	0.53	533701
UACR	rs2277537	15	41857080	A	G	0.597	546427
UACR	rs2433611	15	45665653	A	C	0.259	547360
UACR	rs146311723	15	63804507	T	C	0.824	533701
UACR	rs2470893	15	75019449	T	C	0.326	532366
UACR	rs11078597	17	1618363	T	C	0.813	536684
UACR	rs677888	17	37461018	T	G	0.76	547360
UACR	rs35572189	17	79419025	A	G	0.364	518675
UACR	rs1688031	19	35556640	T	C	0.142	533701
UACR	rs144135542	19	41346120	T	C	0.197	511452
UACR	rs15052	19	41813375	T	C	0.825	516735

UACR	rs838142	19	49252151	A	G	0.72	517917
UACR	rs11912350	22	30748027	T	C	0.758	539625
CKD	rs2484639	1	243462367	A	G	0.51	438949
CKD	rs13391258	2	73848933	T	C	0.24	444737
CKD	rs2580350	2	121996007	A	G	0.55	402682
CKD	rs62300825	4	77205319	A	G	0.2	444622
CKD	rs1458038	4	81164723	T	C	0.31	440290
CKD	rs700221	5	39357175	A	G	0.59	402682
CKD	rs35716097	5	176806636	T	C	0.32	402682
CKD	rs881858	6	43806609	A	G	0.7	439981
CKD	rs9474801	6	54186999	A	G	0.34	444725
CKD	rs12205178	6	160648923	A	G	0.12	444904
CKD	rs11761603	7	1286912	T	C	0.3	341496
CKD	rs10224002	7	151415041	A	G	0.72	440290
CKD	rs4871907	8	23786784	A	C	0.55	402682
CKD	rs1889937	9	71403106	A	G	0.63	388729
CKD	rs3925584	11	30760335	T	C	0.56	440210
CKD	rs7178881	15	39224897	A	C	0.41	444846
CKD	rs1049518	15	45653367	A	G	0.38	440290
CKD	rs149937746	15	53704507	A	G		356896
CKD	rs17730281	15	53907948	A	G	0.23	440290
CKD	rs77924615	16	20392332	A	G	0.2	402682

for MR analysis.

Beta	SE	*P-value*	*R square*	*F*
0.015	0.002	1.780e-09	0.0002	38
0.008	0.001	1.265e-16	0.0003	75
0.007	0.001	1.725e-14	0.0003	60
-0.008	0.001	1.200e-08	0.0002	34
0.006	0.001	2.421e-08	0.0001	31
0.005	0.001	1.680e-08	0.0001	31
-0.005	0.001	2.874e-09	0.0002	37
-0.008	0.001	2.896e-11	0.0002	48
-0.006	0.001	6.397e-09	0.0002	35
-0.005	0.001	3.255e-09	0.0001	33
0.005	0.001	8.053e-09	0.0002	36
-0.01	0.001	1.746e-31	0.0005	126
-0.005	0.001	4.088e-08	0.0001	27
-0.009	0.001	8.651e-23	0.0004	88
-0.008	0.001	9.194e-11	0.0002	39
0.008	0.001	3.484e-14	0.0003	62
0.006	0.001	2.001e-08	0.0002	35
-0.009	0.001	4.971e-21	0.0005	91
-0.005	0.001	1.097e-10	0.0002	37
-0.007	0.001	1.610e-12	0.0002	55
0.007	0.001	1.538e-13	0.0002	59
-0.007	0.001	2.914e-15	0.0003	66
-0.007	0.001	2.280e-18	0.0003	69
0.007	0.001	1.957e-18	0.0003	69
0.01	0.001	8.997e-25	0.0006	116
0.013	0.001	1.081e-26	0.0007	106
-0.007	0.001	3.152e-15	0.0003	64
0.015	0.001	9.535e-43	0.0009	174
0.013	0.002	1.121e-15	0.0003	61
-0.005	0.001	4.655e-08	0.0002	32
-0.01	0.001	3.387e-15	0.0003	63
0.008	0.001	2.631e-12	0.0002	49
0.008	0.001	5.509e-10	0.0002	41
-0.006	0.001	8.325e-09	0.0001	34
0.01	0.001	9.854e-29	0.0005	114
0.005	0.001	3.515e-08	0.0002	32
0.006	0.001	2.422e-11	0.0002	40
0.006	0.001	1.269e-12	0.0002	46
-0.005	0.001	2.713e-08	0.0001	31
0.006	0.001	8.019e-13	0.0002	49
-0.009	0.001	1.350e-15	0.0003	67
0.009	0.001	3.161e-18	0.0003	70
0.007	0.001	3.690e-13	0.0002	57
-0.005	0.001	6.861e-09	0.0001	32

0.005	0.001	7.329e-09	0.0001	31
-0.01	0.001	1.727e-28	0.0005	111
0.006	0.001	2.866e-11	0.0002	43
-0.012	0.001	4.017e-25	0.0005	107
0.006	0.001	7.380e-12	0.0002	44
0.01	0.001	7.463e-16	0.0003	62
-0.009	0.001	6.106e-21	0.0004	91
0.006	0.001	1.863e-08	0.0001	35
-0.008	0.001	2.190e-12	0.0003	48
-0.006	0.001	2.275e-10	0.0002	38
0.007	0.001	2.869e-08	0.0001	31
0.006	0.001	8.224e-14	0.0002	51
-0.017	0.002	7.753e-20	0.0003	81
0.043	0.004	9.170e-29	0.0006	123
0.006	0.001	5.540e-10	0.0001	35
-0.009	0.001	6.131e-18	0.0003	71
0.009	0.001	2.360e-24	0.0004	100
-0.006	0.001	6.414e-10	0.0002	37
-0.006	0.001	8.104e-11	0.0002	42
-0.004	4.30e-04	2.220e-23	0.0002	99
0.002	3.75e-04	1.874e-10	0.0001	41
-0.003	3.58e-04	3.670e-14	0.0001	57
0.003	4.62e-04	2.055e-08	0.0001	31
-0.004	4.78e-04	8.932e-15	0.0001	60
-0.002	3.52e-04	3.138e-09	0.0001	35
0.003	3.79e-04	3.800e-16	0.0001	66
-0.005	4.21e-04	1.331e-32	0.0003	141
-0.002	3.46e-04	3.910e-08	0.0001	30
0.002	3.55e-04	1.596e-09	0.0001	36
0.002	3.81e-04	1.506e-08	0.0001	32
-0.011	1.40e-03	2.527e-14	0.0001	58
0.005	5.53e-04	3.566e-18	0.0001	76
-0.004	7.39e-04	1.366e-09	0.0001	37
0.002	3.47e-04	1.475e-10	0.0001	41
-0.002	3.46e-04	5.458e-13	0.0001	52
0.002	3.60e-04	3.000e-09	0.0001	35
-0.002	4.33e-04	4.401e-08	0.0001	30
0.003	3.63e-04	1.525e-20	0.0002	86
-0.003	3.69e-04	6.402e-18	0.0001	74
0.005	3.56e-04	4.163e-38	0.0003	167
-0.003	3.50e-04	2.182e-14	0.0001	58
0.006	4.16e-04	1.662e-48	0.0004	214
-0.003	4.28e-04	1.388e-09	0.0001	37
0.007	1.08e-03	1.164e-11	0.0001	46
0.004	5.03e-04	2.135e-16	0.0001	67
-0.003	3.78e-04	1.231e-11	0.0001	46
-0.002	3.87e-04	8.768e-09	0.0001	33
-0.008	8.29e-04	9.533e-20	0.0002	83

-0.002	3.45e-04	2.488e-08	0.0001	31
-0.007	3.85e-04	3.586e-64	0.0005	286
0.004	3.59e-04	1.269e-24	0.0002	105
-0.003	3.73e-04	2.296e-15	0.0001	63
-0.002	3.68e-04	3.183e-08	0.0001	31
0.003	3.52e-04	4.760e-19	0.0001	80
0.002	3.69e-04	1.749e-09	0.0001	36
0.003	3.86e-04	2.562e-12	0.0001	49
0.003	4.83e-04	2.016e-10	0.0001	40
0.003	3.45e-04	1.503e-14	0.0001	59
-0.003	3.83e-04	2.812e-11	0.0001	44
0.005	3.84e-04	7.825e-34	0.0003	147
-0.004	5.07e-04	8.786e-17	0.0001	69
-0.002	3.51e-04	5.553e-10	0.0001	38
0.002	3.42e-04	2.977e-09	0.0001	35
0.002	3.99e-04	1.151e-08	0.0001	33
-0.007	3.46e-04	8.310e-102	0.0008	459
0.003	3.80e-04	3.601e-17	0.0001	71
-0.003	3.42e-04	2.997e-15	0.0001	62
0.002	4.11e-04	6.446e-09	0.0001	34
-0.003	3.88e-04	2.945e-12	0.0001	49
-0.006	3.59e-04	2.045e-54	0.0005	241
0.003	4.17e-04	5.238e-10	0.0001	39
0.006	5.51e-04	2.553e-24	0.0002	104
-0.006	6.05e-04	1.372e-26	0.0002	114
-0.006	5.96e-04	7.394e-22	0.0002	92
0.002	3.77e-04	1.857e-08	0.0001	32
-0.002	3.51e-04	1.858e-08	0.0001	32
-0.005	8.96e-04	1.236e-09	0.0001	37
-0.003	4.66e-04	4.331e-12	0.0001	48
-0.007	4.06e-04	3.193e-64	0.0005	286
0.004	5.24e-04	3.234e-12	0.0001	49
-0.002	3.65e-04	2.734e-09	0.0001	35
0.003	4.50e-04	2.766e-13	0.0001	53
-0.005	6.30e-04	6.043e-15	0.0001	61
-0.006	3.78e-04	1.149e-49	0.0004	220
-0.002	3.60e-04	3.496e-09	0.0001	35
0.003	3.64e-04	7.053e-14	0.0001	56
0.003	3.73e-04	1.342e-11	0.0001	46
0.003	4.23e-04	2.382e-09	0.0001	36
-0.003	3.44e-04	3.076e-16	0.0001	67
-0.002	4.32e-04	7.563e-09	0.0001	33
0.003	4.64e-04	4.575e-13	0.0001	52
-0.002	3.59e-04	1.923e-10	0.0001	41
-0.003	4.22e-04	1.012e-09	0.0001	37
-0.004	5.77e-04	1.640e-13	0.0001	54
-0.002	3.51e-04	1.971e-09	0.0001	36
-0.004	3.87e-04	6.091e-24	0.0002	102

0.002	3.50e-04	3.024e-09	0.0001	35
0.003	3.56e-04	2.478e-16	0.0001	67
-0.003	4.58e-04	2.108e-09	0.0001	36
0.007	3.98e-04	2.737e-66	0.0005	296
-0.003	3.63e-04	3.100e-15	0.0001	62
-0.003	3.94e-04	2.084e-17	0.0001	72
0.003	3.52e-04	9.777e-15	0.0001	60
-0.002	3.42e-04	4.773e-09	0.0001	34
0.002	3.55e-04	1.028e-08	0.0001	33
0.003	4.03e-04	5.734e-12	0.0001	47
-0.002	3.77e-04	1.223e-08	0.0001	32
0.003	3.97e-04	3.176e-11	0.0001	44
-0.002	4.30e-04	7.751e-09	0.0001	33
0.002	3.88e-04	9.975e-10	0.0001	37
0.004	5.50e-04	9.204e-12	0.0001	46
0.005	3.61e-04	9.749e-41	0.0003	179
0.003	3.48e-04	4.687e-13	0.0001	52
-0.003	4.80e-04	9.337e-09	0.0001	33
-0.002	3.79e-04	2.840e-10	0.0001	40
-0.002	3.51e-04	1.026e-08	0.0001	33
0.004	4.46e-04	2.696e-18	0.0001	76
0.003	3.44e-04	2.923e-14	0.0001	58
0.002	3.42e-04	3.518e-08	0.0001	30
-0.003	3.50e-04	6.309e-14	0.0001	56
0.003	4.45e-04	5.305e-09	0.0001	34
0.003	3.46e-04	2.858e-13	0.0001	53
0.002	3.94e-04	8.379e-09	0.0001	33
0.004	4.41e-04	2.842e-22	0.0002	94
0.003	3.88e-04	1.716e-14	0.0001	59
-0.005	3.46e-04	3.009e-56	0.0004	250
-0.002	3.54e-04	6.306e-11	0.0001	43
0.002	3.55e-04	2.159e-08	0.0001	31
-0.003	3.61e-04	4.467e-19	0.0001	80
-0.002	3.69e-04	1.226e-10	0.0001	41
0.002	3.53e-04	1.623e-09	0.0001	36
-0.002	3.90e-04	3.761e-08	0.0001	30
-0.004	3.44e-04	7.078e-34	0.0003	147
-0.003	4.58e-04	1.519e-08	0.0001	32
0.004	4.53e-04	1.874e-19	0.0001	81
0.004	6.10e-04	1.060e-10	0.0001	42
-0.004	4.37e-04	1.340e-16	0.0001	68
0.003	3.89e-04	5.302e-13	0.0001	52
-0.002	3.91e-04	5.362e-09	0.0001	34
-0.003	4.10e-04	3.967e-15	0.0001	62
0.002	3.54e-04	9.717e-09	0.0001	33
-0.008	1.23e-03	1.562e-10	0.0001	41
0.003	4.67e-04	3.823e-08	0.0001	30
0.002	3.50e-04	1.100e-08	0.0001	33

0.002	3.47e-04	1.734e-08	0.0001	32
0.002	3.69e-04	2.131e-09	0.0001	36
0.003	3.67e-04	2.376e-13	0.0001	54
0.003	3.55e-04	4.313e-18	0.0001	75
-0.003	3.54e-04	7.000e-17	0.0001	70
-0.002	3.95e-04	2.343e-09	0.0001	36
-0.002	3.45e-04	5.488e-13	0.0001	52
-0.003	4.47e-04	6.737e-09	0.0001	34
0.005	4.08e-04	3.283e-34	0.0003	149
0.008	1.20e-03	1.651e-11	0.0001	45
0.002	3.93e-04	2.030e-08	0.0001	31
0.01	4.52e-04	1.210e-99	0.0009	449
0.002	3.59e-04	8.620e-12	0.0001	47
-0.003	3.57e-04	2.352e-13	0.0001	54
0.003	3.91e-04	2.855e-12	0.0001	49
-0.003	3.90e-04	1.152e-11	0.0001	46
-0.002	3.55e-04	2.456e-09	0.0001	36
-0.003	4.86e-04	1.110e-09	0.0001	37
-0.002	3.50e-04	1.686e-09	0.0001	36
0.002	3.63e-04	3.389e-10	0.0001	39
0.004	3.59e-04	8.474e-30	0.0002	129
-0.006	3.99e-04	5.025e-49	0.0004	217
0.006	8.92e-04	7.635e-13	0.0001	51
-0.004	4.13e-04	3.836e-25	0.0002	107
-0.008	4.62e-04	5.342e-60	0.0005	267
0.003	3.44e-04	3.734e-16	0.0001	66
-0.003	5.83e-04	3.760e-09	0.0001	35
0.003	4.18e-04	9.844e-16	0.0001	64
-0.002	3.52e-04	4.887e-08	0.0001	30
0.003	4.50e-04	7.774e-09	0.0001	33
0.005	3.62e-04	2.193e-43	0.0003	191
0.002	3.65e-04	4.677e-10	0.0001	39
-0.002	3.69e-04	1.903e-09	0.0001	36
-0.002	3.69e-04	4.044e-08	0.0001	30
-0.003	3.44e-04	9.606e-22	0.0002	92
0.002	3.49e-04	1.431e-08	0.0001	32
-0.002	3.68e-04	5.266e-09	0.0001	34
-0.002	3.62e-04	2.904e-09	0.0001	35
-0.003	5.78e-04	1.379e-08	0.0001	32
-0.003	3.65e-04	1.007e-13	0.0001	55
0.002	3.96e-04	4.855e-08	0.0001	30
-0.003	4.50e-04	2.338e-09	0.0001	36
0.003	4.13e-04	8.434e-11	0.0001	42
-0.002	3.92e-04	2.177e-10	0.0001	40
-0.003	4.68e-04	1.304e-08	0.0001	32
0.006	5.73e-04	6.689e-23	0.0002	97
-0.003	3.76e-04	2.327e-17	0.0001	72
0.012	0.002	1.358e-08	0.0001	32

-0.013	0.002	3.607e-08	0.0001	30
-0.027	0.002	1.338e-27	0.0002	119
0.016	0.002	1.633e-15	0.0001	63
-0.019	0.003	1.981e-08	0.0001	32
-0.047	0.008	2.839e-10	0.0001	40
0.018	0.003	1.364e-08	0.0001	32
0.017	0.002	6.196e-17	0.0001	70
-0.016	0.003	9.215e-09	0.0001	33
-0.019	0.002	2.553e-18	0.0001	76
-0.02	0.003	2.434e-11	0.0001	45
-0.026	0.004	1.693e-11	0.0001	45
0.011	0.002	1.855e-08	0.0001	32
0.03	0.004	2.032e-13	0.0001	54
-0.013	0.002	6.278e-09	0.0001	34
0.014	0.002	9.715e-13	0.0001	51
0.014	0.002	5.740e-13	0.0001	52
0.023	0.004	1.027e-09	0.0001	37
0.012	0.002	5.898e-10	0.0001	38
0.013	0.002	6.269e-10	0.0001	38
-0.017	0.002	1.286e-12	0.0001	50
-0.022	0.002	2.032e-26	0.0002	113
0.02	0.002	2.168e-17	0.0001	72
-0.016	0.003	2.934e-10	0.0001	40
0.012	0.002	3.089e-08	0.0001	31
-0.012	0.002	3.616e-09	0.0001	35
-0.012	0.002	7.385e-09	0.0001	33
-0.015	0.003	1.401e-08	0.0001	32
0.015	0.002	6.890e-14	0.0001	56
0.201	0.008	4.830e-126	0.0011	570
0.011	0.002	1.560e-08	0.0001	32
-0.121	0.013	9.229e-20	0.0002	83
0.017	0.002	2.451e-12	0.0001	49
-0.012	0.002	5.499e-10	0.0001	38
-0.013	0.002	1.788e-08	0.0001	32
-0.012	0.002	2.021e-09	0.0001	36
0.014	0.002	1.168e-11	0.0001	46
-0.015	0.002	1.559e-13	0.0001	54
-0.014	0.002	1.572e-11	0.0001	45
0.015	0.002	1.756e-13	0.0001	54
-0.018	0.002	9.107e-15	0.0001	60
-0.015	0.003	6.640e-09	0.0001	34
0.023	0.002	1.075e-26	0.0002	114
-0.016	0.003	7.133e-10	0.0001	38
-0.014	0.002	7.299e-10	0.0001	38
-0.012	0.002	3.046e-08	0.0001	31
-0.019	0.003	1.636e-11	0.0001	45
0.015	0.003	3.151e-08	0.0001	31
0.017	0.003	2.170e-10	0.0001	40

0.017	0.002	8.783e-14	0.0001	56
-0.013	0.002	2.437e-08	0.0001	31
-0.077	0.009	2.950e-17	0.0002	71
-0.06	0.011	2.738e-08	0.0001	31
0.055	0.01	1.691e-08	0.0001	31
-0.095	0.012	2.629e-16	0.0002	67
-0.059	0.01	4.206e-09	0.0001	35
-0.072	0.01	2.192e-13	0.0001	54
0.078	0.011	8.202e-14	0.0001	56
0.062	0.01	1.189e-09	0.0001	37
0.052	0.01	4.606e-08	0.0001	30
0.093	0.014	3.087e-11	0.0001	44
-0.067	0.012	1.352e-08	0.0001	32
-0.108	0.01	2.651e-26	0.0003	113
-0.063	0.01	9.909e-11	0.0001	42
-0.062	0.01	5.146e-10	0.0001	39
0.08	0.009	4.675e-18	0.0002	76
-0.054	0.009	4.140e-09	0.0001	35
0.079	0.009	5.422e-17	0.0002	70
-0.422	0.076	2.768e-08	0.0001	31
-0.087	0.011	2.677e-15	0.0001	62
-0.224	0.013	6.383e-69	0.0008	305
