# Supplementary material for: Causal Links Between Renal Function and Cardiac Structure, Function, and Disease Risk
Source: Glob Heart. 2024 Nov 6;19(1):83. doi: 10.5334/gh.1366 (PMC11546326; doi:10.5334/gh.1366)
Supplement: Table S3. — Effect estimates of the renal function on cardiovascular diseases. [file gh-19-1-1366-s7.pdf]

Table S3. Effect estimates of the renal function on car

Exposure	Outcome	method	No. of SNP	b
BUN	Coronary artery disease	MR Egger	55	-0.263
BUN	Coronary artery disease	Weighted median	55	0.329
BUN	Coronary artery disease	Inverse variance weighted	55	0.409
BUN	Coronary artery disease	Simple mode	55	0.027
BUN	Coronary artery disease	Weighted mode	55	0.128
BUN	Coronary artery disease finngen	MR Egger	59	0.249
BUN	Coronary artery disease finngen	Weighted median	59	0.244
BUN	Coronary artery disease finngen	Inverse variance weighted	59	0.610
BUN	Coronary artery disease finngen	Simple mode	59	0.179
BUN	Coronary artery disease finngen	Weighted mode	59	0.085
BUN	Myocardial infarction finngen	MR Egger	59	0.203
BUN	Myocardial infarction finngen	Weighted median	59	0.560
BUN	Myocardial infarction finngen	Inverse variance weighted	59	0.661
BUN	Myocardial infarction finngen	Simple mode	59	0.479
BUN	Myocardial infarction finngen	Weighted mode	59	0.349
BUN	Stroke finngen	MR Egger	61	-0.978
BUN	Stroke finngen	Weighted median	61	-0.099
BUN	Stroke finngen	Inverse variance weighted	61	0.354
BUN	Stroke finngen	Simple mode	61	-0.460
BUN	Stroke finngen	Weighted mode	61	-0.378
BUN	Stroke	MR Egger	62	0.093
BUN	Stroke	Weighted median	62	-0.172
BUN	Stroke	Inverse variance weighted	62	-0.117
BUN	Stroke	Simple mode	62	0.014
BUN	Stroke	Weighted mode	62	0.094
BUN	Atrial fibrillation finngen	MR Egger	57	-0.483
BUN	Atrial fibrillation finngen	Weighted median	57	0.140
BUN	Atrial fibrillation finngen	Inverse variance weighted	57	0.491
BUN	Atrial fibrillation finngen	Simple mode	57	-0.019
BUN	Atrial fibrillation finngen	Weighted mode	57	-0.019
BUN	Atrial fibrillation	MR Egger	59	0.703
BUN	Atrial fibrillation	Weighted median	59	0.331
BUN	Atrial fibrillation	Inverse variance weighted	59	0.014
BUN	Atrial fibrillation	Simple mode	59	-0.891
BUN	Atrial fibrillation	Weighted mode	59	0.613
BUN	Heart failure finngen	MR Egger	59	0.212
BUN	Heart failure finngen	Weighted median	59	0.585
BUN	Heart failure finngen	Inverse variance weighted	59	0.298
BUN	Heart failure finngen	Simple mode	59	0.772
BUN	Heart failure finngen	Weighted mode	59	0.906
BUN	Heart failure	MR Egger	61	-0.038
BUN	Heart failure	Weighted median	61	-0.025
BUN	Heart failure	Inverse variance weighted	61	0.170

BUN	Heart failure	Simple mode	61	-0.314
BUN	Heart failure	Weighted mode	61	-0.314
BUN	Myocardial infarction	MR Egger	59	-0.086
BUN	Myocardial infarction	Weighted median	59	0.553
BUN	Myocardial infarction	Inverse variance weighted	59	0.421
BUN	Myocardial infarction	Simple mode	59	1.162
BUN	Myocardial infarction	Weighted mode	59	1.019
eGFR	Stroke finngen	MR Egger	165	1.239
eGFR	Stroke finngen	Weighted median	165	0.285
eGFR	Stroke finngen	Inverse variance weighted	165	0.483
eGFR	Stroke finngen	Simple mode	165	1.685
eGFR	Stroke finngen	Weighted mode	165	0.185
eGFR	Stroke	MR Egger	171	0.348
eGFR	Stroke	Weighted median	171	0.314
eGFR	Stroke	Inverse variance weighted	171	0.109
eGFR	Stroke	Simple mode	171	0.342
eGFR	Stroke	Weighted mode	171	0.396
eGFR	Atrial fibrillation finngen	MR Egger	161	1.034
eGFR	Atrial fibrillation finngen	Weighted median	161	0.256
eGFR	Atrial fibrillation finngen	Inverse variance weighted	161	0.498
eGFR	Atrial fibrillation finngen	Simple mode	161	-0.354
eGFR	Atrial fibrillation finngen	Weighted mode	161	-0.872
eGFR	Atrial fibrillation	MR Egger	164	0.860
eGFR	Atrial fibrillation	Weighted median	164	0.345
eGFR	Atrial fibrillation	Inverse variance weighted	164	0.215
eGFR	Atrial fibrillation	Simple mode	164	0.526
eGFR	Atrial fibrillation	Weighted mode	164	0.472
eGFR	Coronary artery disease	MR Egger	159	0.834
eGFR	Coronary artery disease	Weighted median	159	0.201
eGFR	Coronary artery disease	Inverse variance weighted	159	0.146
eGFR	Coronary artery disease	Simple mode	159	0.835
eGFR	Coronary artery disease	Weighted mode	159	-0.431
eGFR	Coronary artery disease finngen	MR Egger	159	1.029
eGFR	Coronary artery disease finngen	Weighted median	159	0.134
eGFR	Coronary artery disease finngen	Inverse variance weighted	159	0.465
eGFR	Coronary artery disease finngen	Simple mode	159	0.278
eGFR	Coronary artery disease finngen	Weighted mode	159	0.077
eGFR	Heart failure finngen	MR Egger	166	-0.437
eGFR	Heart failure finngen	Weighted median	166	0.396
eGFR	Heart failure finngen	Inverse variance weighted	166	0.215
eGFR	Heart failure finngen	Simple mode	166	1.424
eGFR	Heart failure finngen	Weighted mode	166	1.125
eGFR	Heart failure	MR Egger	170	0.703
eGFR	Heart failure	Weighted median	170	0.614
eGFR	Heart failure	Inverse variance weighted	170	0.473
eGFR	Heart failure	Simple mode	170	0.282
eGFR	Heart failure	Weighted mode	170	0.669
eGFR	Myocardial infarction finngen	MR Egger	163	0.682

eGFR	Myocardial infarction finngen	Weighted median	163	-0.105
eGFR	Myocardial infarction finngen	Inverse variance weighted	163	-0.051
eGFR	Myocardial infarction finngen	Simple mode	163	-0.719
eGFR	Myocardial infarction finngen	Weighted mode	163	-0.539
eGFR	Myocardial infarction	MR Egger	164	1.347
eGFR	Myocardial infarction	Weighted median	164	0.377
eGFR	Myocardial infarction	Inverse variance weighted	164	0.429
eGFR	Myocardial infarction	Simple mode	164	0.270
eGFR	Myocardial infarction	Weighted mode	164	1.708
UACR	Stroke finngen	MR Egger	46	0.027
UACR	Stroke finngen	Weighted median	46	0.227
UACR	Stroke finngen	Inverse variance weighted	46	0.251
UACR	Stroke finngen	Simple mode	46	0.451
UACR	Stroke finngen	Weighted mode	46	0.103
UACR	Stroke	MR Egger	49	0.050
UACR	Stroke	Weighted median	49	0.143
UACR	Stroke	Inverse variance weighted	49	0.167
UACR	Stroke	Simple mode	49	0.158
UACR	Stroke	Weighted mode	49	0.110
UACR	Atrial fibrillation finngen	MR Egger	45	0.271
UACR	Atrial fibrillation finngen	Weighted median	45	0.281
UACR	Atrial fibrillation finngen	Inverse variance weighted	45	0.381
UACR	Atrial fibrillation finngen	Simple mode	45	0.471
UACR	Atrial fibrillation finngen	Weighted mode	45	0.331
UACR	Coronary artery disease	MR Egger	45	-0.197
UACR	Coronary artery disease	Weighted median	45	0.068
UACR	Coronary artery disease	Inverse variance weighted	45	0.231
UACR	Coronary artery disease	Simple mode	45	0.129
UACR	Coronary artery disease	Weighted mode	45	0.013
UACR	Coronary artery disease finngen	MR Egger	44	0.100
UACR	Coronary artery disease finngen	Weighted median	44	0.237
UACR	Coronary artery disease finngen	Inverse variance weighted	44	0.389
UACR	Coronary artery disease finngen	Simple mode	44	0.081
UACR	Coronary artery disease finngen	Weighted mode	44	0.044
UACR	Myocardial infarction finngen	MR Egger	45	0.350
UACR	Myocardial infarction finngen	Weighted median	45	0.217
UACR	Myocardial infarction finngen	Inverse variance weighted	45	0.348
UACR	Myocardial infarction finngen	Simple mode	45	0.077
UACR	Myocardial infarction finngen	Weighted mode	45	0.178
UACR	Myocardial infarction	MR Egger	45	-0.151
UACR	Myocardial infarction	Weighted median	45	0.178
UACR	Myocardial infarction	Inverse variance weighted	45	0.353
UACR	Myocardial infarction	Simple mode	45	0.406
UACR	Myocardial infarction	Weighted mode	45	0.075
UACR	Atrial fibrillation	MR Egger	48	-0.253
UACR	Atrial fibrillation	Weighted median	48	-0.080
UACR	Atrial fibrillation	Inverse variance weighted	48	0.071
UACR	Atrial fibrillation	Simple mode	48	-0.004

UACR	Atrial fibrillation	Weighted mode	48	-0.116
UACR	Heart failure finngen	MR Egger	45	0.217
UACR	Heart failure finngen	Weighted median	45	0.301
UACR	Heart failure finngen	Inverse variance weighted	45	0.096
UACR	Heart failure finngen	Simple mode	45	0.672
UACR	Heart failure finngen	Weighted mode	45	0.531
UACR	Heart failure	MR Egger	50	-0.246
UACR	Heart failure	Weighted median	50	0.007
UACR	Heart failure	Inverse variance weighted	50	0.020
UACR	Heart failure	Simple mode	50	0.407
UACR	Heart failure	Weighted mode	50	-0.083
CKD	Stroke finngen	MR Egger	20	0.055
CKD	Stroke finngen	Weighted median	20	0.012
CKD	Stroke finngen	Inverse variance weighted	20	0.003
CKD	Stroke finngen	Simple mode	20	-0.006
CKD	Stroke finngen	Weighted mode	20	0.010
CKD	Stroke	MR Egger	19	-0.050
CKD	Stroke	Weighted median	19	0.003
CKD	Stroke	Inverse variance weighted	19	0.023
CKD	Stroke	Simple mode	19	0.077
CKD	Stroke	Weighted mode	19	-0.019
CKD	Atrial fibrillation finngen	MR Egger	19	0.108
CKD	Atrial fibrillation finngen	Weighted median	19	0.057
CKD	Atrial fibrillation finngen	Inverse variance weighted	19	0.018
CKD	Atrial fibrillation finngen	Simple mode	19	-0.087
CKD	Atrial fibrillation finngen	Weighted mode	19	0.084
CKD	Atrial fibrillation	MR Egger	19	0.045
CKD	Atrial fibrillation	Weighted median	19	0.042
CKD	Atrial fibrillation	Inverse variance weighted	19	0.011
CKD	Atrial fibrillation	Simple mode	19	0.062
CKD	Atrial fibrillation	Weighted mode	19	0.049
CKD	Coronary artery disease	MR Egger	18	0.032
CKD	Coronary artery disease	Weighted median	18	0.026
CKD	Coronary artery disease	Inverse variance weighted	18	0.009
CKD	Coronary artery disease	Simple mode	18	0.069
CKD	Coronary artery disease	Weighted mode	18	0.031
CKD	Coronary artery disease finngen	MR Egger	18	0.033
CKD	Coronary artery disease finngen	Weighted median	18	0.001
CKD	Coronary artery disease finngen	Inverse variance weighted	18	0.001
CKD	Coronary artery disease finngen	Simple mode	18	0.070
CKD	Coronary artery disease finngen	Weighted mode	18	0.016
CKD	Heart failure finngen	MR Egger	20	0.104
CKD	Heart failure finngen	Weighted median	20	0.058
CKD	Heart failure finngen	Inverse variance weighted	20	0.015
CKD	Heart failure finngen	Simple mode	20	0.044
CKD	Heart failure finngen	Weighted mode	20	0.060
CKD	Heart failure	MR Egger	19	0.106
CKD	Heart failure	Weighted median	19	0.028

CKD	Heart failure	Inverse variance weighted	19	0.004
CKD	Heart failure	Simple mode	19	0.001
CKD	Heart failure	Weighted mode	19	0.050
CKD	Myocardial infarction finngen	MR Egger	19	0.010
CKD	Myocardial infarction finngen	Weighted median	19	0.008
CKD	Myocardial infarction finngen	Inverse variance weighted	19	-0.013
CKD	Myocardial infarction finngen	Simple mode	19	0.076
CKD	Myocardial infarction finngen	Weighted mode	19	0.034
CKD	Myocardial infarction	MR Egger	17	0.068
CKD	Myocardial infarction	Weighted median	17	0.008
CKD	Myocardial infarction	Inverse variance weighted	17	-0.011
CKD	Myocardial infarction	Simple mode	17	-0.101
CKD	Myocardial infarction	Weighted mode	17	0.063

diovascular diseases.

se	pval	or	or_lci95	or_uci95	Source
0.504	0.604	0.769	0.286	2.064	Primary
0.164	0.046	1.389	1.006	1.917	Primary
0.171	0.017	1.505	1.077	2.103	Primary
0.351	0.939	1.027	0.516	2.045	Primary
0.258	0.621	1.137	0.686	1.885	Primary
0.783	0.752	1.283	0.277	5.946	Validation
0.253	0.334	1.276	0.778	2.093	Validation
0.240	0.011	1.840	1.148	2.948	Validation
0.588	0.762	1.196	0.378	3.783	Validation
0.428	0.844	1.088	0.471	2.516	Validation
0.798	0.800	1.225	0.257	5.850	Validation
0.305	0.067	1.750	0.962	3.184	Validation
0.245	0.007	1.938	1.198	3.132	Validation
0.642	0.459	1.614	0.458	5.684	Validation
0.513	0.499	1.418	0.518	3.877	Validation
0.692	0.163	0.376	0.097	1.459	Validation
0.251	0.694	0.906	0.553	1.483	Validation
0.218	0.104	1.425	0.929	2.185	Validation
0.510	0.370	0.631	0.232	1.714	Validation
0.360	0.298	0.685	0.338	1.388	Validation
0.461	0.840	1.098	0.445	2.709	Primary
0.183	0.348	0.842	0.588	1.206	Primary
0.145	0.420	0.890	0.670	1.182	Primary
0.469	0.976	1.014	0.405	2.541	Primary
0.345	0.786	1.099	0.559	2.159	Primary
0.925	0.604	0.617	0.101	3.779	Validation
0.300	0.639	1.151	0.639	2.071	Validation
0.288	0.089	1.635	0.929	2.877	Validation
0.613	0.976	0.982	0.295	3.262	Validation
0.539	0.973	0.982	0.341	2.824	Validation
0.583	0.233	2.019	0.644	6.328	Primary
0.218	0.129	1.393	0.908	2.137	Primary
0.188	0.940	1.014	0.701	1.467	Primary
0.577	0.128	0.410	0.132	1.271	Primary
0.380	0.112	1.846	0.876	3.890	Primary
0.754	0.780	1.236	0.282	5.417	Validation
0.294	0.047	1.794	1.008	3.192	Validation
0.230	0.194	1.348	0.859	2.115	Validation
0.731	0.295	2.165	0.517	9.073	Validation
0.623	0.151	2.474	0.730	8.384	Validation
0.630	0.952	0.963	0.280	3.312	Primary
0.239	0.917	0.976	0.611	1.557	Primary
0.203	0.403	1.185	0.796	1.763	Primary

0.600	0.604	0.731	0.225	2.371	Primary
0.513	0.544	0.731	0.267	1.999	Primary
0.702	0.904	0.918	0.232	3.637	Primary
0.243	0.023	1.739	1.080	2.800	Primary
0.227	0.063	1.523	0.977	2.375	Primary
0.720	0.112	3.195	0.779	13.110	Primary
0.650	0.123	2.770	0.774	9.910	Primary
0.697	0.078	3.451	0.880	13.536	Validation
0.364	0.434	1.330	0.651	2.716	Validation
0.279	0.083	1.621	0.939	2.798	Validation
0.869	0.054	5.392	0.981	29.636	Validation
0.578	0.749	1.203	0.388	3.734	Validation
0.582	0.550	1.417	0.453	4.431	Primary
0.287	0.275	1.369	0.779	2.404	Primary
0.227	0.632	1.115	0.715	1.740	Primary
0.707	0.630	1.407	0.352	5.631	Primary
0.441	0.371	1.485	0.625	3.528	Primary
0.991	0.299	2.812	0.403	19.621	Validation
0.443	0.564	1.291	0.541	3.079	Validation
0.391	0.203	1.645	0.764	3.542	Validation
1.341	0.792	0.702	0.051	9.726	Validation
1.022	0.395	0.418	0.056	3.101	Validation
0.642	0.182	2.364	0.672	8.319	Primary
0.308	0.263	1.412	0.772	2.582	Primary
0.251	0.391	1.240	0.758	2.026	Primary
0.953	0.582	1.692	0.262	10.949	Primary
0.671	0.482	1.604	0.431	5.969	Primary
0.589	0.159	2.302	0.726	7.307	Primary
0.246	0.412	1.223	0.756	1.979	Primary
0.233	0.531	1.157	0.733	1.825	Primary
0.913	0.362	2.305	0.385	13.799	Primary
0.834	0.606	0.650	0.127	3.332	Primary
0.752	0.173	2.798	0.640	12.226	Validation
0.362	0.712	1.143	0.562	2.325	Validation
0.303	0.124	1.593	0.880	2.883	Validation
0.939	0.767	1.321	0.210	8.315	Validation
0.581	0.895	1.080	0.346	3.370	Validation
0.770	0.571	0.646	0.143	2.923	Validation
0.395	0.317	1.486	0.685	3.224	Validation
0.308	0.485	1.239	0.678	2.265	Validation
1.066	0.184	4.154	0.514	33.586	Validation
0.835	0.180	3.080	0.600	15.817	Validation
0.708	0.322	2.020	0.505	8.086	Primary
0.337	0.069	1.847	0.954	3.579	Primary
0.276	0.086	1.605	0.935	2.756	Primary
0.845	0.739	1.325	0.253	6.951	Primary
0.730	0.361	1.953	0.467	8.175	Primary
0.980	0.487	1.978	0.290	13.487	Validation

0.472	0.825	0.901	0.357	2.273	Validation
0.392	0.896	0.950	0.441	2.047	Validation
1.156	0.535	0.487	0.051	4.700	Validation
0.702	0.443	0.583	0.147	2.308	Validation
0.789	0.090	3.847	0.819	18.068	Primary
0.362	0.297	1.458	0.717	2.964	Primary
0.310	0.167	1.535	0.836	2.820	Primary
1.235	0.828	1.309	0.116	14.738	Primary
1.308	0.193	5.516	0.425	71.551	Primary
0.227	0.907	1.027	0.658	1.603	Validation
0.129	0.079	1.254	0.974	1.615	Validation
0.103	0.015	1.285	1.050	1.572	Validation
0.293	0.131	1.570	0.884	2.789	Validation
0.175	0.559	1.109	0.787	1.562	Validation
0.204	0.808	1.051	0.705	1.568	Primary
0.106	0.177	1.154	0.938	1.419	Primary
0.079	0.035	1.182	1.012	1.379	Primary
0.218	0.473	1.171	0.764	1.795	Primary
0.158	0.488	1.117	0.819	1.523	Primary
0.282	0.342	1.311	0.754	2.279	Validation
0.163	0.084	1.325	0.963	1.823	Validation
0.127	0.003	1.463	1.140	1.878	Validation
0.322	0.151	1.602	0.851	3.014	Validation
0.210	0.123	1.392	0.922	2.102	Validation
0.184	0.290	0.821	0.573	1.177	Primary
0.084	0.417	1.071	0.908	1.263	Primary
0.097	0.017	1.260	1.042	1.523	Primary
0.220	0.560	1.138	0.739	1.753	Primary
0.102	0.896	1.014	0.829	1.239	Primary
0.258	0.701	1.105	0.666	1.831	Validation
0.130	0.067	1.268	0.983	1.634	Validation
0.119	0.001	1.475	1.167	1.864	Validation
0.359	0.823	1.084	0.536	2.192	Validation
0.192	0.819	1.045	0.718	1.521	Validation
0.331	0.296	1.419	0.742	2.712	Validation
0.178	0.222	1.243	0.877	1.762	Validation
0.149	0.020	1.416	1.057	1.897	Validation
0.366	0.834	1.080	0.527	2.214	Validation
0.222	0.427	1.195	0.773	1.847	Validation
0.230	0.514	0.860	0.548	1.349	Primary
0.120	0.138	1.195	0.945	1.512	Primary
0.115	0.002	1.424	1.137	1.783	Primary
0.317	0.208	1.501	0.806	2.795	Primary
0.165	0.651	1.078	0.781	1.489	Primary
0.205	0.222	0.776	0.520	1.159	Primary
0.103	0.435	0.923	0.755	1.129	Primary
0.098	0.471	1.073	0.885	1.302	Primary
0.240	0.987	0.996	0.623	1.593	Primary

0.132	0.387	0.891	0.687	1.155	Primary
0.233	0.358	1.242	0.786	1.961	Validation
0.149	0.043	1.352	1.009	1.810	Validation
0.105	0.364	1.100	0.895	1.352	Validation
0.345	0.058	1.958	0.996	3.849	Validation
0.219	0.019	1.701	1.108	2.613	Validation
0.235	0.301	0.782	0.493	1.240	Primary
0.121	0.955	1.007	0.795	1.276	Primary
0.107	0.855	1.020	0.827	1.258	Primary
0.263	0.129	1.502	0.897	2.515	Primary
0.156	0.598	0.920	0.677	1.250	Primary
0.099	0.584	1.057	0.871	1.283	Validation
0.037	0.743	1.012	0.942	1.088	Validation
0.041	0.941	1.003	0.925	1.088	Validation
0.062	0.925	0.994	0.881	1.122	Validation
0.038	0.807	1.010	0.936	1.089	Validation
0.067	0.465	0.951	0.834	1.085	Primary
0.030	0.921	1.003	0.946	1.063	Primary
0.026	0.388	1.023	0.972	1.077	Primary
0.050	0.138	1.080	0.980	1.190	Primary
0.031	0.542	0.981	0.922	1.043	Primary
0.111	0.346	1.114	0.896	1.384	Validation
0.043	0.191	1.058	0.972	1.152	Validation
0.047	0.697	1.019	0.929	1.117	Validation
0.094	0.364	0.916	0.762	1.101	Validation
0.047	0.089	1.088	0.992	1.192	Validation
0.069	0.524	1.046	0.914	1.197	Primary
0.030	0.162	1.043	0.983	1.105	Primary
0.027	0.701	1.011	0.958	1.066	Primary
0.051	0.233	1.064	0.964	1.175	Primary
0.032	0.151	1.050	0.985	1.118	Primary
0.055	0.565	1.033	0.927	1.150	Primary
0.024	0.267	1.027	0.980	1.076	Primary
0.022	0.696	1.009	0.966	1.054	Primary
0.048	0.169	1.071	0.975	1.176	Primary
0.027	0.259	1.032	0.979	1.088	Primary
0.072	0.650	1.034	0.897	1.192	Validation
0.035	0.987	1.001	0.934	1.072	Validation
0.031	0.982	1.001	0.941	1.064	Validation
0.064	0.288	1.073	0.946	1.216	Validation
0.038	0.674	1.016	0.943	1.096	Validation
0.078	0.199	1.110	0.952	1.293	Validation
0.044	0.192	1.059	0.971	1.155	Validation
0.034	0.654	1.015	0.950	1.084	Validation
0.095	0.647	1.045	0.868	1.258	Validation
0.051	0.251	1.062	0.961	1.173	Validation
0.060	0.097	1.112	0.988	1.252	Primary
0.034	0.412	1.028	0.962	1.099	Primary

0.025	0.877	1.004	0.956	1.055	Primary
0.074	0.987	1.001	0.866	1.158	Primary
0.039	0.213	1.051	0.975	1.133	Primary
0.105	0.925	1.010	0.822	1.241	Validation
0.048	0.869	1.008	0.918	1.107	Validation
0.044	0.768	0.987	0.906	1.076	Validation
0.080	0.350	1.079	0.924	1.261	Validation
0.049	0.503	1.034	0.939	1.139	Validation
0.093	0.476	1.070	0.892	1.283	Primary
0.038	0.827	1.008	0.937	1.085	Primary
0.037	0.758	0.989	0.920	1.063	Primary
0.102	0.338	0.904	0.741	1.104	Primary
0.048	0.210	1.065	0.969	1.169	Primary
