# Supplementary material for: Causal Links Between Renal Function and Cardiac Structure, Function, and Disease Risk
Source: Glob Heart. 2024 Nov 6;19(1):83. doi: 10.5334/gh.1366 (PMC11546326; doi:10.5334/gh.1366)
Supplement: Table S4. — Test results for pleiotropy and heterogeneity – causal effects of the renal function on cardiovascular diseases, structure and function. [file gh-19-1-1366-s8.pdf]

Table S4. Test results for pleiotropy and heterogeneity - causal effects of the Renal function o diseases, structure and function.

Exposure	Outcome	Cochran's Q test		MR-Egger		[R-PRESS(
		Q	*P* value	Egger_ intercept	*P* value	*global test*
BUN	Coronary artery disease	168.06	1.238E-13	0.006	0.163	<0.001
BUN	Coronary artery disease finng(	150.32	3.975E-10	0.003	0.630	<0.001
UACR	Stroke finngen	70.31	9.278E-03	0.004	0.275	0.012
UACR	Stroke	68.15	2.942E-02	0.002	0.536	0.03
UACR	Coronary artery disease	172.32	4.294E-17	0.009	0.010	<0.001
UACR	Coronary artery disease finng(	95.98	6.444E-06	0.006	0.213	0.001
UACR	Myocardial infarction finngen	89.71	5.752E-05	0.000	0.995	<0.001
UACR	Myocardial infarction	106.82	3.842E-07	0.010	0.017	<0.001
BUN	LVEF	92.95	6.653E-03	-0.002	0.609	0.008
BUN	Prox PA Diam Indexed	83.00	3.205E-02	-0.002	0.547	0.039
CKD	PA Aorta ratio	14.61	6.886E-01	-0.003	0.514	0.74
CKD	Prox PA Diam Indexed	17.59	5.503E-01	0.000	0.986	0.621
eGFR	Asc Aorta Diam Indexed	257.52	2.088E-05	0.000	0.848	<0.001
eGFR	LVSV Indexed	237.93	3.765E-04	-0.001	0.764	0.001
eGFR	Prox PA Diam Indexed	232.36	1.251E-03	-0.002	0.399	0.001
eGFR	RA Max Indexed	188.53	1.571E-01	0.002	0.209	0.136
eGFR	RA Min Indexed	230.30	1.685E-03	0.001	0.591	0.001
eGFR	RVEDV Indexed	305.75	5.997E-10	0.000	0.831	<0.001
eGFR	RVSV Indexed	289.19	3.195E-08	0.000	0.858	<0.001

on cardiovascular

MR-PRESSO Outlier-corrected	
Beta	***P* value**
0.435	4.95E-03
0.753	1.36E-03
NA	NA
NA	NA
0.345	4.38E-04
0.463	1.11E-04
0.403	1.20E-03
0.440	1.66E-04
NA	NA
NA	NA
NA	NA
NA	NA
0.672	2.69E-03
NA	NA
1.028	8.06E-07
NA	NA
0.725	9.68E-04
NA	NA
0.734	1.46E-03
