# Supplementary material for: Causal Links Between Renal Function and Cardiac Structure, Function, and Disease Risk
Source: Glob Heart. 2024 Nov 6;19(1):83. doi: 10.5334/gh.1366 (PMC11546326; doi:10.5334/gh.1366)
Supplement: Table S5. — Effect estimates of the cardiovascular diseases, structure and function on renal function using reverse MR. [file gh-19-1-1366-s9.pdf]

Table S5. Effect estimates of the cardiovascular diseases, structure

Exposure	Outcome	method	No. of SNP
Asc Aorta Diam Indexed	BUN	MR Egger	12
Asc Aorta Diam Indexed	BUN	Weighted median	12
Asc Aorta Diam Indexed	BUN	Inverse variance weighted	12
Asc Aorta Diam Indexed	BUN	Simple mode	12
Asc Aorta Diam Indexed	BUN	Weighted mode	12
Atrial fibrillation	BUN	MR Egger	93
Atrial fibrillation	BUN	Weighted median	93
Atrial fibrillation	BUN	Inverse variance weighted	93
Atrial fibrillation	BUN	Simple mode	93
Atrial fibrillation	BUN	Weighted mode	93
Atrial fibrillation finngen	BUN	MR Egger	68
Atrial fibrillation finngen	BUN	Weighted median	68
Atrial fibrillation finngen	BUN	Inverse variance weighted	68
Atrial fibrillation finngen	BUN	Simple mode	68
Atrial fibrillation finngen	BUN	Weighted mode	68
Coronary artery disease	BUN	MR Egger	140
Coronary artery disease	BUN	Weighted median	140
Coronary artery disease	BUN	Inverse variance weighted	140
Coronary artery disease	BUN	Simple mode	140
Coronary artery disease	BUN	Weighted mode	140
Coronary artery disease finngen	BUN	MR Egger	46
Coronary artery disease finngen	BUN	Weighted median	46
Coronary artery disease finngen	BUN	Inverse variance weighted	46
Coronary artery disease finngen	BUN	Simple mode	46
Coronary artery disease finngen	BUN	Weighted mode	46
Heart failure	BUN	MR Egger	7
Heart failure	BUN	Weighted median	7
Heart failure	BUN	Inverse variance weighted	7
Heart failure	BUN	Simple mode	7
Heart failure	BUN	Weighted mode	7
Heart failure finngen	BUN	MR Egger	3
Heart failure finngen	BUN	Weighted median	3
Heart failure finngen	BUN	Inverse variance weighted	3
Heart failure finngen	BUN	Simple mode	3
Heart failure finngen	BUN	Weighted mode	3
LATEF	BUN	MR Egger	4
LATEF	BUN	Weighted median	4
LATEF	BUN	Inverse variance weighted	4
LATEF	BUN	Simple mode	4
LATEF	BUN	Weighted mode	4
LV Mass Indexed	BUN	MR Egger	7
LV Mass Indexed	BUN	Weighted median	7
LV Mass Indexed	BUN	Inverse variance weighted	7

LV Mass Indexed	BUN	Simple mode	7
LV Mass Indexed	BUN	Weighted mode	7
LVEDV Indexed	BUN	MR Egger	12
LVEDV Indexed	BUN	Weighted median	12
LVEDV Indexed	BUN	Inverse variance weighted	12
LVEDV Indexed	BUN	Simple mode	12
LVEDV Indexed	BUN	Weighted mode	12
LVEF	BUN	MR Egger	14
LVEF	BUN	Weighted median	14
LVEF	BUN	Inverse variance weighted	14
LVEF	BUN	Simple mode	14
LVEF	BUN	Weighted mode	14
LVESV Indexed	BUN	MR Egger	18
LVESV Indexed	BUN	Weighted median	18
LVESV Indexed	BUN	Inverse variance weighted	18
LVESV Indexed	BUN	Simple mode	18
LVESV Indexed	BUN	Weighted mode	18
LVSV Indexed	BUN	MR Egger	9
LVSV Indexed	BUN	Weighted median	9
LVSV Indexed	BUN	Inverse variance weighted	9
LVSV Indexed	BUN	Simple mode	9
LVSV Indexed	BUN	Weighted mode	9
Myocardial infarction	BUN	MR Egger	61
Myocardial infarction	BUN	Weighted median	61
Myocardial infarction	BUN	Inverse variance weighted	61
Myocardial infarction	BUN	Simple mode	61
Myocardial infarction	BUN	Weighted mode	61
Myocardial infarction finngen	BUN	MR Egger	30
Myocardial infarction finngen	BUN	Weighted median	30
Myocardial infarction finngen	BUN	Inverse variance weighted	30
Myocardial infarction finngen	BUN	Simple mode	30
Myocardial infarction finngen	BUN	Weighted mode	30
Myocardial interstitial fibrosis	BUN	MR Egger	7
Myocardial interstitial fibrosis	BUN	Weighted median	7
Myocardial interstitial fibrosis	BUN	Inverse variance weighted	7
Myocardial interstitial fibrosis	BUN	Simple mode	7
Myocardial interstitial fibrosis	BUN	Weighted mode	7
PA Aorta ratio	BUN	MR Egger	17
PA Aorta ratio	BUN	Weighted median	17
PA Aorta ratio	BUN	Inverse variance weighted	17
PA Aorta ratio	BUN	Simple mode	17
PA Aorta ratio	BUN	Weighted mode	17
Prox PA Diam Indexed	BUN	MR Egger	12
Prox PA Diam Indexed	BUN	Weighted median	12
Prox PA Diam Indexed	BUN	Inverse variance weighted	12
Prox PA Diam Indexed	BUN	Simple mode	12
Prox PA Diam Indexed	BUN	Weighted mode	12

RA FAC	BUN	MR Egger	4
RA FAC	BUN	Weighted median	4
RA FAC	BUN	Inverse variance weighted	4
RA FAC	BUN	Simple mode	4
RA FAC	BUN	Weighted mode	4
RA Max Indexed	BUN	MR Egger	4
RA Max Indexed	BUN	Weighted median	4
RA Max Indexed	BUN	Inverse variance weighted	4
RA Max Indexed	BUN	Simple mode	4
RA Max Indexed	BUN	Weighted mode	4
RA Min Indexed	BUN	MR Egger	5
RA Min Indexed	BUN	Weighted median	5
RA Min Indexed	BUN	Inverse variance weighted	5
RA Min Indexed	BUN	Simple mode	5
RA Min Indexed	BUN	Weighted mode	5
RVEDV Indexed	BUN	MR Egger	5
RVEDV Indexed	BUN	Weighted median	5
RVEDV Indexed	BUN	Inverse variance weighted	5
RVEDV Indexed	BUN	Simple mode	5
RVEDV Indexed	BUN	Weighted mode	5
RVEF	BUN	MR Egger	9
RVEF	BUN	Weighted median	9
RVEF	BUN	Inverse variance weighted	9
RVEF	BUN	Simple mode	9
RVEF	BUN	Weighted mode	9
RVESV Indexed	BUN	MR Egger	14
RVESV Indexed	BUN	Weighted median	14
RVESV Indexed	BUN	Inverse variance weighted	14
RVESV Indexed	BUN	Simple mode	14
RVESV Indexed	BUN	Weighted mode	14
RVESV LVESV ratio	BUN	MR Egger	5
RVESV LVESV ratio	BUN	Weighted median	5
RVESV LVESV ratio	BUN	Inverse variance weighted	5
RVESV LVESV ratio	BUN	Simple mode	5
RVESV LVESV ratio	BUN	Weighted mode	5
Stroke	BUN	MR Egger	21
Stroke	BUN	Weighted median	21
Stroke	BUN	Inverse variance weighted	21
Stroke	BUN	Simple mode	21
Stroke	BUN	Weighted mode	21
Stroke finngen	BUN	MR Egger	10
Stroke finngen	BUN	Weighted median	10
Stroke finngen	BUN	Inverse variance weighted	10
Stroke finngen	BUN	Simple mode	10
Stroke finngen	BUN	Weighted mode	10
Asc Aorta Diam Indexed	CKD	MR Egger	15
Asc Aorta Diam Indexed	CKD	Weighted median	15

Asc Aorta Diam Indexed	CKD	Inverse variance weighted	15
Asc Aorta Diam Indexed	CKD	Simple mode	15
Asc Aorta Diam Indexed	CKD	Weighted mode	15
Atrial fibrillation	CKD	MR Egger	99
Atrial fibrillation	CKD	Weighted median	99
Atrial fibrillation	CKD	Inverse variance weighted	99
Atrial fibrillation	CKD	Simple mode	99
Atrial fibrillation	CKD	Weighted mode	99
Atrial fibrillation finngen	CKD	MR Egger	73
Atrial fibrillation finngen	CKD	Weighted median	73
Atrial fibrillation finngen	CKD	Inverse variance weighted	73
Atrial fibrillation finngen	CKD	Simple mode	73
Atrial fibrillation finngen	CKD	Weighted mode	73
Coronary artery disease	CKD	MR Egger	145
Coronary artery disease	CKD	Weighted median	145
Coronary artery disease	CKD	Inverse variance weighted	145
Coronary artery disease	CKD	Simple mode	145
Coronary artery disease	CKD	Weighted mode	145
Coronary artery disease finngen	CKD	MR Egger	45
Coronary artery disease finngen	CKD	Weighted median	45
Coronary artery disease finngen	CKD	Inverse variance weighted	45
Coronary artery disease finngen	CKD	Simple mode	45
Coronary artery disease finngen	CKD	Weighted mode	45
Heart failure	CKD	MR Egger	9
Heart failure	CKD	Weighted median	9
Heart failure	CKD	Inverse variance weighted	9
Heart failure	CKD	Simple mode	9
Heart failure	CKD	Weighted mode	9
Heart failure finngen	CKD	MR Egger	4
Heart failure finngen	CKD	Weighted median	4
Heart failure finngen	CKD	Inverse variance weighted	4
Heart failure finngen	CKD	Simple mode	4
Heart failure finngen	CKD	Weighted mode	4
LATEF	CKD	MR Egger	4
LATEF	CKD	Weighted median	4
LATEF	CKD	Inverse variance weighted	4
LATEF	CKD	Simple mode	4
LATEF	CKD	Weighted mode	4
LV Mass Indexed	CKD	MR Egger	8
LV Mass Indexed	CKD	Weighted median	8
LV Mass Indexed	CKD	Inverse variance weighted	8
LV Mass Indexed	CKD	Simple mode	8
LV Mass Indexed	CKD	Weighted mode	8
LVEDV Indexed	CKD	MR Egger	13
LVEDV Indexed	CKD	Weighted median	13
LVEDV Indexed	CKD	Inverse variance weighted	13
LVEDV Indexed	CKD	Simple mode	13

LVEDV Indexed	CKD	Weighted mode	13
LVEF	CKD	MR Egger	14
LVEF	CKD	Weighted median	14
LVEF	CKD	Inverse variance weighted	14
LVEF	CKD	Simple mode	14
LVEF	CKD	Weighted mode	14
LVESV Indexed	CKD	MR Egger	19
LVESV Indexed	CKD	Weighted median	19
LVESV Indexed	CKD	Inverse variance weighted	19
LVESV Indexed	CKD	Simple mode	19
LVESV Indexed	CKD	Weighted mode	19
LVSV Indexed	CKD	MR Egger	10
LVSV Indexed	CKD	Weighted median	10
LVSV Indexed	CKD	Inverse variance weighted	10
LVSV Indexed	CKD	Simple mode	10
LVSV Indexed	CKD	Weighted mode	10
Myocardial infarction	CKD	MR Egger	64
Myocardial infarction	CKD	Weighted median	64
Myocardial infarction	CKD	Inverse variance weighted	64
Myocardial infarction	CKD	Simple mode	64
Myocardial infarction	CKD	Weighted mode	64
Myocardial infarction finngen	CKD	MR Egger	28
Myocardial infarction finngen	CKD	Weighted median	28
Myocardial infarction finngen	CKD	Inverse variance weighted	28
Myocardial infarction finngen	CKD	Simple mode	28
Myocardial infarction finngen	CKD	Weighted mode	28
Myocardial interstitial fibrosis	CKD	MR Egger	7
Myocardial interstitial fibrosis	CKD	Weighted median	7
Myocardial interstitial fibrosis	CKD	Inverse variance weighted	7
Myocardial interstitial fibrosis	CKD	Simple mode	7
Myocardial interstitial fibrosis	CKD	Weighted mode	7
PA Aorta ratio	CKD	MR Egger	17
PA Aorta ratio	CKD	Weighted median	17
PA Aorta ratio	CKD	Inverse variance weighted	17
PA Aorta ratio	CKD	Simple mode	17
PA Aorta ratio	CKD	Weighted mode	17
Prox PA Diam Indexed	CKD	MR Egger	14
Prox PA Diam Indexed	CKD	Weighted median	14
Prox PA Diam Indexed	CKD	Inverse variance weighted	14
Prox PA Diam Indexed	CKD	Simple mode	14
Prox PA Diam Indexed	CKD	Weighted mode	14
RA FAC	CKD	MR Egger	4
RA FAC	CKD	Weighted median	4
RA FAC	CKD	Inverse variance weighted	4
RA FAC	CKD	Simple mode	4
RA FAC	CKD	Weighted mode	4
RA Max Indexed	CKD	MR Egger	6

RA Max Indexed	CKD	Weighted median	6
RA Max Indexed	CKD	Inverse variance weighted	6
RA Max Indexed	CKD	Simple mode	6
RA Max Indexed	CKD	Weighted mode	6
RA Min Indexed	CKD	MR Egger	7
RA Min Indexed	CKD	Weighted median	7
RA Min Indexed	CKD	Inverse variance weighted	7
RA Min Indexed	CKD	Simple mode	7
RA Min Indexed	CKD	Weighted mode	7
RVEDV Indexed	CKD	MR Egger	6
RVEDV Indexed	CKD	Weighted median	6
RVEDV Indexed	CKD	Inverse variance weighted	6
RVEDV Indexed	CKD	Simple mode	6
RVEDV Indexed	CKD	Weighted mode	6
RVEF	CKD	MR Egger	9
RVEF	CKD	Weighted median	9
RVEF	CKD	Inverse variance weighted	9
RVEF	CKD	Simple mode	9
RVEF	CKD	Weighted mode	9
RVESV Indexed	CKD	MR Egger	15
RVESV Indexed	CKD	Weighted median	15
RVESV Indexed	CKD	Inverse variance weighted	15
RVESV Indexed	CKD	Simple mode	15
RVESV Indexed	CKD	Weighted mode	15
RVESV LVESV ratio	CKD	MR Egger	5
RVESV LVESV ratio	CKD	Weighted median	5
RVESV LVESV ratio	CKD	Inverse variance weighted	5
RVESV LVESV ratio	CKD	Simple mode	5
RVESV LVESV ratio	CKD	Weighted mode	5
Stroke	CKD	MR Egger	22
Stroke	CKD	Weighted median	22
Stroke	CKD	Inverse variance weighted	22
Stroke	CKD	Simple mode	22
Stroke	CKD	Weighted mode	22
Stroke finngen	CKD	MR Egger	10
Stroke finngen	CKD	Weighted median	10
Stroke finngen	CKD	Inverse variance weighted	10
Stroke finngen	CKD	Simple mode	10
Stroke finngen	CKD	Weighted mode	10
Asc Aorta Diam Indexed	eGFR	MR Egger	15
Asc Aorta Diam Indexed	eGFR	Weighted median	15
Asc Aorta Diam Indexed	eGFR	Inverse variance weighted	15
Asc Aorta Diam Indexed	eGFR	Simple mode	15
Asc Aorta Diam Indexed	eGFR	Weighted mode	15
Atrial fibrillation	eGFR	MR Egger	95
Atrial fibrillation	eGFR	Weighted median	95
Atrial fibrillation	eGFR	Inverse variance weighted	95

Atrial fibrillation	eGFR	Simple mode	95
Atrial fibrillation	eGFR	Weighted mode	95
Atrial fibrillation finngen	eGFR	MR Egger	70
Atrial fibrillation finngen	eGFR	Weighted median	70
Atrial fibrillation finngen	eGFR	Inverse variance weighted	70
Atrial fibrillation finngen	eGFR	Simple mode	70
Atrial fibrillation finngen	eGFR	Weighted mode	70
Coronary artery disease	eGFR	MR Egger	134
Coronary artery disease	eGFR	Weighted median	134
Coronary artery disease	eGFR	Inverse variance weighted	134
Coronary artery disease	eGFR	Simple mode	134
Coronary artery disease	eGFR	Weighted mode	134
Coronary artery disease finngen	eGFR	MR Egger	43
Coronary artery disease finngen	eGFR	Weighted median	43
Coronary artery disease finngen	eGFR	Inverse variance weighted	43
Coronary artery disease finngen	eGFR	Simple mode	43
Coronary artery disease finngen	eGFR	Weighted mode	43
Heart failure	eGFR	MR Egger	6
Heart failure	eGFR	Weighted median	6
Heart failure	eGFR	Inverse variance weighted	6
Heart failure	eGFR	Simple mode	6
Heart failure	eGFR	Weighted mode	6
Heart failure finngen	eGFR	MR Egger	3
Heart failure finngen	eGFR	Weighted median	3
Heart failure finngen	eGFR	Inverse variance weighted	3
Heart failure finngen	eGFR	Simple mode	3
Heart failure finngen	eGFR	Weighted mode	3
LATEF	eGFR	MR Egger	4
LATEF	eGFR	Weighted median	4
LATEF	eGFR	Inverse variance weighted	4
LATEF	eGFR	Simple mode	4
LATEF	eGFR	Weighted mode	4
LV Mass Indexed	eGFR	MR Egger	7
LV Mass Indexed	eGFR	Weighted median	7
LV Mass Indexed	eGFR	Inverse variance weighted	7
LV Mass Indexed	eGFR	Simple mode	7
LV Mass Indexed	eGFR	Weighted mode	7
LVEDV Indexed	eGFR	MR Egger	11
LVEDV Indexed	eGFR	Weighted median	11
LVEDV Indexed	eGFR	Inverse variance weighted	11
LVEDV Indexed	eGFR	Simple mode	11
LVEDV Indexed	eGFR	Weighted mode	11
LVEF	eGFR	MR Egger	13
LVEF	eGFR	Weighted median	13
LVEF	eGFR	Inverse variance weighted	13
LVEF	eGFR	Simple mode	13
LVEF	eGFR	Weighted mode	13

LVESV Indexed	eGFR	MR Egger	18
LVESV Indexed	eGFR	Weighted median	18
LVESV Indexed	eGFR	Inverse variance weighted	18
LVESV Indexed	eGFR	Simple mode	18
LVESV Indexed	eGFR	Weighted mode	18
LVSV Indexed	eGFR	MR Egger	8
LVSV Indexed	eGFR	Weighted median	8
LVSV Indexed	eGFR	Inverse variance weighted	8
LVSV Indexed	eGFR	Simple mode	8
LVSV Indexed	eGFR	Weighted mode	8
Myocardial infarction	eGFR	MR Egger	55
Myocardial infarction	eGFR	Weighted median	55
Myocardial infarction	eGFR	Inverse variance weighted	55
Myocardial infarction	eGFR	Simple mode	55
Myocardial infarction	eGFR	Weighted mode	55
Myocardial infarction finngen	eGFR	MR Egger	26
Myocardial infarction finngen	eGFR	Weighted median	26
Myocardial infarction finngen	eGFR	Inverse variance weighted	26
Myocardial infarction finngen	eGFR	Simple mode	26
Myocardial infarction finngen	eGFR	Weighted mode	26
Myocardial interstitial fibrosis	eGFR	MR Egger	6
Myocardial interstitial fibrosis	eGFR	Weighted median	6
Myocardial interstitial fibrosis	eGFR	Inverse variance weighted	6
Myocardial interstitial fibrosis	eGFR	Simple mode	6
Myocardial interstitial fibrosis	eGFR	Weighted mode	6
PA Aorta ratio	eGFR	MR Egger	15
PA Aorta ratio	eGFR	Weighted median	15
PA Aorta ratio	eGFR	Inverse variance weighted	15
PA Aorta ratio	eGFR	Simple mode	15
PA Aorta ratio	eGFR	Weighted mode	15
Prox PA Diam Indexed	eGFR	MR Egger	15
Prox PA Diam Indexed	eGFR	Weighted median	15
Prox PA Diam Indexed	eGFR	Inverse variance weighted	15
Prox PA Diam Indexed	eGFR	Simple mode	15
Prox PA Diam Indexed	eGFR	Weighted mode	15
RA FAC	eGFR	MR Egger	4
RA FAC	eGFR	Weighted median	4
RA FAC	eGFR	Inverse variance weighted	4
RA FAC	eGFR	Simple mode	4
RA FAC	eGFR	Weighted mode	4
RA Max Indexed	eGFR	MR Egger	6
RA Max Indexed	eGFR	Weighted median	6
RA Max Indexed	eGFR	Inverse variance weighted	6
RA Max Indexed	eGFR	Simple mode	6
RA Max Indexed	eGFR	Weighted mode	6
RA Min Indexed	eGFR	MR Egger	7
RA Min Indexed	eGFR	Weighted median	7

RA Min Indexed	eGFR	Inverse variance weighted	7
RA Min Indexed	eGFR	Simple mode	7
RA Min Indexed	eGFR	Weighted mode	7
RVEDV Indexed	eGFR	MR Egger	5
RVEDV Indexed	eGFR	Weighted median	5
RVEDV Indexed	eGFR	Inverse variance weighted	5
RVEDV Indexed	eGFR	Simple mode	5
RVEDV Indexed	eGFR	Weighted mode	5
RVEF	eGFR	MR Egger	8
RVEF	eGFR	Weighted median	8
RVEF	eGFR	Inverse variance weighted	8
RVEF	eGFR	Simple mode	8
RVEF	eGFR	Weighted mode	8
RVESV Indexed	eGFR	MR Egger	13
RVESV Indexed	eGFR	Weighted median	13
RVESV Indexed	eGFR	Inverse variance weighted	13
RVESV Indexed	eGFR	Simple mode	13
RVESV Indexed	eGFR	Weighted mode	13
RVESV LVESV ratio	eGFR	MR Egger	5
RVESV LVESV ratio	eGFR	Weighted median	5
RVESV LVESV ratio	eGFR	Inverse variance weighted	5
RVESV LVESV ratio	eGFR	Simple mode	5
RVESV LVESV ratio	eGFR	Weighted mode	5
Stroke	eGFR	MR Egger	19
Stroke	eGFR	Weighted median	19
Stroke	eGFR	Inverse variance weighted	19
Stroke	eGFR	Simple mode	19
Stroke	eGFR	Weighted mode	19
Stroke finngen	eGFR	MR Egger	9
Stroke finngen	eGFR	Weighted median	9
Stroke finngen	eGFR	Inverse variance weighted	9
Stroke finngen	eGFR	Simple mode	9
Stroke finngen	eGFR	Weighted mode	9
Asc Aorta Diam Indexed	UACR	MR Egger	14
Asc Aorta Diam Indexed	UACR	Weighted median	14
Asc Aorta Diam Indexed	UACR	Inverse variance weighted	14
Asc Aorta Diam Indexed	UACR	Simple mode	14
Asc Aorta Diam Indexed	UACR	Weighted mode	14
Atrial fibrillation	UACR	MR Egger	96
Atrial fibrillation	UACR	Weighted median	96
Atrial fibrillation	UACR	Inverse variance weighted	96
Atrial fibrillation	UACR	Simple mode	96
Atrial fibrillation	UACR	Weighted mode	96
Atrial fibrillation finngen	UACR	MR Egger	70
Atrial fibrillation finngen	UACR	Weighted median	70
Atrial fibrillation finngen	UACR	Inverse variance weighted	70
Atrial fibrillation finngen	UACR	Simple mode	70

Atrial fibrillation finngen	UACR	Weighted mode	70
Coronary artery disease	UACR	MR Egger	140
Coronary artery disease	UACR	Weighted median	140
Coronary artery disease	UACR	Inverse variance weighted	140
Coronary artery disease	UACR	Simple mode	140
Coronary artery disease	UACR	Weighted mode	140
Coronary artery disease finngen	UACR	MR Egger	44
Coronary artery disease finngen	UACR	Weighted median	44
Coronary artery disease finngen	UACR	Inverse variance weighted	44
Coronary artery disease finngen	UACR	Simple mode	44
Coronary artery disease finngen	UACR	Weighted mode	44
Heart failure	UACR	MR Egger	9
Heart failure	UACR	Weighted median	9
Heart failure	UACR	Inverse variance weighted	9
Heart failure	UACR	Simple mode	9
Heart failure	UACR	Weighted mode	9
Heart failure finngen	UACR	MR Egger	4
Heart failure finngen	UACR	Weighted median	4
Heart failure finngen	UACR	Inverse variance weighted	4
Heart failure finngen	UACR	Simple mode	4
Heart failure finngen	UACR	Weighted mode	4
LATEF	UACR	MR Egger	4
LATEF	UACR	Weighted median	4
LATEF	UACR	Inverse variance weighted	4
LATEF	UACR	Simple mode	4
LATEF	UACR	Weighted mode	4
LV Mass Indexed	UACR	MR Egger	8
LV Mass Indexed	UACR	Weighted median	8
LV Mass Indexed	UACR	Inverse variance weighted	8
LV Mass Indexed	UACR	Simple mode	8
LV Mass Indexed	UACR	Weighted mode	8
LVEDV Indexed	UACR	MR Egger	11
LVEDV Indexed	UACR	Weighted median	11
LVEDV Indexed	UACR	Inverse variance weighted	11
LVEDV Indexed	UACR	Simple mode	11
LVEDV Indexed	UACR	Weighted mode	11
LVEF	UACR	MR Egger	14
LVEF	UACR	Weighted median	14
LVEF	UACR	Inverse variance weighted	14
LVEF	UACR	Simple mode	14
LVEF	UACR	Weighted mode	14
LVESV Indexed	UACR	MR Egger	19
LVESV Indexed	UACR	Weighted median	19
LVESV Indexed	UACR	Inverse variance weighted	19
LVESV Indexed	UACR	Simple mode	19
LVESV Indexed	UACR	Weighted mode	19
LVSV Indexed	UACR	MR Egger	9

LVSV Indexed	UACR	Weighted median	9
LVSV Indexed	UACR	Inverse variance weighted	9
LVSV Indexed	UACR	Simple mode	9
LVSV Indexed	UACR	Weighted mode	9
Myocardial infarction	UACR	MR Egger	62
Myocardial infarction	UACR	Weighted median	62
Myocardial infarction	UACR	Inverse variance weighted	62
Myocardial infarction	UACR	Simple mode	62
Myocardial infarction	UACR	Weighted mode	62
Myocardial infarction finngen	UACR	MR Egger	26
Myocardial infarction finngen	UACR	Weighted median	26
Myocardial infarction finngen	UACR	Inverse variance weighted	26
Myocardial infarction finngen	UACR	Simple mode	26
Myocardial infarction finngen	UACR	Weighted mode	26
Myocardial interstitial fibrosis	UACR	MR Egger	7
Myocardial interstitial fibrosis	UACR	Weighted median	7
Myocardial interstitial fibrosis	UACR	Inverse variance weighted	7
Myocardial interstitial fibrosis	UACR	Simple mode	7
Myocardial interstitial fibrosis	UACR	Weighted mode	7
PA Aorta ratio	UACR	MR Egger	16
PA Aorta ratio	UACR	Weighted median	16
PA Aorta ratio	UACR	Inverse variance weighted	16
PA Aorta ratio	UACR	Simple mode	16
PA Aorta ratio	UACR	Weighted mode	16
Prox PA Diam Indexed	UACR	MR Egger	14
Prox PA Diam Indexed	UACR	Weighted median	14
Prox PA Diam Indexed	UACR	Inverse variance weighted	14
Prox PA Diam Indexed	UACR	Simple mode	14
Prox PA Diam Indexed	UACR	Weighted mode	14
RA FAC	UACR	MR Egger	4
RA FAC	UACR	Weighted median	4
RA FAC	UACR	Inverse variance weighted	4
RA FAC	UACR	Simple mode	4
RA FAC	UACR	Weighted mode	4
RA Max Indexed	UACR	MR Egger	5
RA Max Indexed	UACR	Weighted median	5
RA Max Indexed	UACR	Inverse variance weighted	5
RA Max Indexed	UACR	Simple mode	5
RA Max Indexed	UACR	Weighted mode	5
RA Min Indexed	UACR	MR Egger	6
RA Min Indexed	UACR	Weighted median	6
RA Min Indexed	UACR	Inverse variance weighted	6
RA Min Indexed	UACR	Simple mode	6
RA Min Indexed	UACR	Weighted mode	6
RVEDV Indexed	UACR	MR Egger	5
RVEDV Indexed	UACR	Weighted median	5
RVEDV Indexed	UACR	Inverse variance weighted	5

RVEDV Indexed	UACR	Simple mode	5
RVEDV Indexed	UACR	Weighted mode	5
RVEF	UACR	MR Egger	9
RVEF	UACR	Weighted median	9
RVEF	UACR	Inverse variance weighted	9
RVEF	UACR	Simple mode	9
RVEF	UACR	Weighted mode	9
RVESV Indexed	UACR	MR Egger	14
RVESV Indexed	UACR	Weighted median	14
RVESV Indexed	UACR	Inverse variance weighted	14
RVESV Indexed	UACR	Simple mode	14
RVESV Indexed	UACR	Weighted mode	14
RVESV LVESV ratio	UACR	MR Egger	5
RVESV LVESV ratio	UACR	Weighted median	5
RVESV LVESV ratio	UACR	Inverse variance weighted	5
RVESV LVESV ratio	UACR	Simple mode	5
RVESV LVESV ratio	UACR	Weighted mode	5
Stroke	UACR	MR Egger	19
Stroke	UACR	Weighted median	19
Stroke	UACR	Inverse variance weighted	19
Stroke	UACR	Simple mode	19
Stroke	UACR	Weighted mode	19
Stroke finngen	UACR	MR Egger	10
Stroke finngen	UACR	Weighted median	10
Stroke finngen	UACR	Inverse variance weighted	10
Stroke finngen	UACR	Simple mode	10
Stroke finngen	UACR	Weighted mode	10

: and function on Renal function using reverse MR.

b	se	pval	lo_ci	up_ci	or	or_lci95	or_uci95
0.037	0.028	0.215	-0.018	0.091	1.037	0.983	1.095
-0.008	0.008	0.295	-0.023	0.007	0.992	0.977	1.007
-0.003	0.006	0.581	-0.015	0.008	0.997	0.985	1.008
-0.013	0.012	0.327	-0.037	0.011	0.988	0.964	1.012
-0.013	0.012	0.311	-0.037	0.011	0.987	0.964	1.011
0.001	0.003	0.800	-0.006	0.007	1.001	0.994	1.007
0.001	0.003	0.712	-0.004	0.006	1.001	0.996	1.006
0.002	0.002	0.316	-0.002	0.005	1.002	0.998	1.005
0.005	0.005	0.347	-0.005	0.016	1.005	0.995	1.016
0.001	0.003	0.572	-0.004	0.006	1.001	0.996	1.006
0.002	0.003	0.523	-0.004	0.008	1.002	0.996	1.008
0.001	0.002	0.660	-0.004	0.006	1.001	0.996	1.006
0.002	0.001	0.280	-0.001	0.005	1.002	0.999	1.005
0.003	0.005	0.499	-0.006	0.012	1.003	0.994	1.012
0.002	0.002	0.347	-0.002	0.007	1.002	0.998	1.007
0.000	0.004	0.946	-0.008	0.008	1.000	0.992	1.008
0.004	0.003	0.260	-0.003	0.010	1.004	0.997	1.010
0.002	0.002	0.283	-0.002	0.007	1.002	0.998	1.007
-0.003	0.007	0.687	-0.017	0.011	0.997	0.984	1.011
0.004	0.004	0.334	-0.004	0.013	1.004	0.996	1.013
-0.006	0.006	0.306	-0.018	0.006	0.994	0.982	1.006
0.002	0.003	0.571	-0.005	0.009	1.002	0.995	1.009
0.001	0.003	0.816	-0.005	0.006	1.001	0.995	1.006
0.005	0.008	0.531	-0.011	0.021	1.005	0.989	1.021
0.006	0.007	0.397	-0.007	0.018	1.006	0.993	1.019
0.003	0.033	0.926	-0.061	0.067	1.003	0.941	1.070
-0.002	0.010	0.811	-0.021	0.017	0.998	0.979	1.017
-0.007	0.012	0.545	-0.030	0.016	0.993	0.970	1.016
-0.010	0.018	0.611	-0.046	0.026	0.990	0.955	1.026
-0.001	0.012	0.963	-0.024	0.023	0.999	0.976	1.023
0.025	0.044	0.672	-0.061	0.110	1.025	0.941	1.116
0.006	0.009	0.507	-0.011	0.022	1.006	0.989	1.023
0.005	0.007	0.461	-0.009	0.020	1.005	0.991	1.020
0.004	0.011	0.746	-0.018	0.027	1.004	0.982	1.027
0.006	0.011	0.616	-0.015	0.028	1.006	0.985	1.028
-0.106	0.145	0.539	-0.390	0.178	0.899	0.677	1.194
-0.013	0.012	0.292	-0.036	0.011	0.987	0.965	1.011
-0.013	0.010	0.216	-0.033	0.008	0.987	0.967	1.008
0.000	0.021	1.000	-0.040	0.040	1.000	0.961	1.041
-0.001	0.019	0.980	-0.038	0.037	0.999	0.963	1.038
-0.002	0.005	0.719	-0.011	0.008	0.998	0.989	1.008
0.001	0.001	0.512	-0.001	0.003	1.001	0.999	1.003
0.000	0.001	0.919	-0.002	0.002	1.000	0.998	1.002

0.001	0.002	0.762	-0.003	0.004	1.001	0.997	1.004
0.001	0.001	0.610	-0.002	0.003	1.001	0.998	1.003
0.010	0.027	0.727	-0.043	0.062	1.010	0.958	1.064
-0.015	0.010	0.141	-0.035	0.005	0.985	0.966	1.005
-0.013	0.009	0.150	-0.030	0.005	0.987	0.970	1.005
-0.018	0.018	0.331	-0.053	0.017	0.982	0.948	1.017
-0.015	0.020	0.456	-0.054	0.023	0.985	0.948	1.024
-0.002	0.024	0.926	-0.050	0.045	0.998	0.951	1.046
-0.002	0.007	0.777	-0.015	0.011	0.998	0.985	1.011
-0.003	0.005	0.503	-0.013	0.007	0.997	0.987	1.007
-0.006	0.012	0.645	-0.028	0.017	0.995	0.972	1.018
-0.007	0.011	0.515	-0.029	0.014	0.993	0.972	1.014
0.009	0.019	0.633	-0.028	0.046	1.009	0.973	1.047
-0.001	0.007	0.880	-0.014	0.012	0.999	0.986	1.012
-0.002	0.005	0.657	-0.012	0.007	0.998	0.988	1.007
-0.004	0.010	0.683	-0.025	0.016	0.996	0.975	1.016
-0.002	0.009	0.832	-0.019	0.016	0.998	0.981	1.016
0.056	0.037	0.176	-0.017	0.130	1.058	0.983	1.139
0.019	0.010	0.057	-0.001	0.038	1.019	0.999	1.039
0.014	0.011	0.205	-0.007	0.035	1.014	0.993	1.035
0.016	0.015	0.325	-0.013	0.045	1.016	0.987	1.046
0.020	0.013	0.177	-0.006	0.046	1.020	0.994	1.047
0.001	0.005	0.822	-0.009	0.011	1.001	0.991	1.011
0.004	0.003	0.237	-0.003	0.010	1.004	0.997	1.010
-0.004	0.003	0.160	-0.009	0.001	0.996	0.992	1.001
0.003	0.009	0.779	-0.015	0.020	1.003	0.985	1.021
0.005	0.004	0.265	-0.004	0.014	1.005	0.996	1.014
0.001	0.007	0.883	-0.013	0.015	1.001	0.987	1.015
0.000	0.004	0.951	-0.007	0.007	1.000	0.993	1.007
0.000	0.003	0.993	-0.006	0.006	1.000	0.994	1.006
0.001	0.009	0.915	-0.016	0.018	1.001	0.984	1.018
-0.005	0.007	0.458	-0.019	0.008	0.995	0.982	1.008
-0.013	0.059	0.837	-0.128	0.102	0.987	0.880	1.108
0.011	0.011	0.343	-0.011	0.032	1.011	0.989	1.033
-0.004	0.010	0.694	-0.024	0.016	0.996	0.977	1.016
0.013	0.016	0.442	-0.018	0.045	1.013	0.982	1.046
0.013	0.014	0.384	-0.014	0.041	1.013	0.986	1.042
-0.028	0.039	0.478	-0.105	0.048	0.972	0.901	1.049
-0.005	0.007	0.466	-0.019	0.009	0.995	0.981	1.009
-0.005	0.006	0.441	-0.017	0.007	0.995	0.983	1.007
-0.006	0.016	0.709	-0.036	0.025	0.994	0.964	1.025
-0.007	0.013	0.598	-0.032	0.018	0.993	0.968	1.018
0.028	0.045	0.552	-0.061	0.116	1.028	0.941	1.124
-0.004	0.009	0.624	-0.022	0.013	0.996	0.978	1.013
0.000	0.009	0.998	-0.017	0.017	1.000	0.983	1.017
-0.006	0.015	0.685	-0.036	0.024	0.994	0.964	1.024
-0.006	0.013	0.662	-0.032	0.020	0.994	0.969	1.020

-0.105	0.097	0.394	-0.296	0.086	0.900	0.744	1.090
0.007	0.013	0.621	-0.019	0.032	1.007	0.981	1.033
-0.005	0.018	0.790	-0.041	0.031	0.995	0.960	1.031
0.013	0.017	0.517	-0.021	0.047	1.013	0.979	1.048
0.012	0.016	0.503	-0.019	0.043	1.012	0.981	1.044
0.025	0.067	0.743	-0.107	0.158	1.026	0.899	1.171
0.002	0.013	0.881	-0.024	0.027	1.002	0.977	1.028
-0.004	0.011	0.726	-0.025	0.017	0.996	0.975	1.018
0.007	0.018	0.740	-0.029	0.042	1.007	0.972	1.043
0.006	0.018	0.756	-0.028	0.040	1.006	0.972	1.041
-0.013	0.031	0.711	-0.074	0.049	0.987	0.928	1.050
-0.007	0.010	0.525	-0.027	0.014	0.993	0.974	1.014
-0.005	0.008	0.544	-0.021	0.011	0.995	0.979	1.011
-0.006	0.014	0.701	-0.033	0.021	0.994	0.968	1.022
-0.006	0.014	0.701	-0.032	0.021	0.994	0.968	1.021
0.023	0.149	0.887	-0.269	0.316	1.023	0.764	1.371
-0.044	0.014	0.002	-0.071	-0.016	0.957	0.931	0.984
-0.030	0.014	0.039	-0.058	-0.001	0.971	0.944	0.999
-0.046	0.021	0.090	-0.087	-0.006	0.955	0.917	0.995
-0.045	0.023	0.124	-0.090	0.000	0.956	0.914	1.000
-0.010	0.027	0.708	-0.063	0.042	0.990	0.939	1.043
0.005	0.009	0.550	-0.012	0.023	1.005	0.988	1.023
0.001	0.007	0.939	-0.013	0.014	1.001	0.987	1.014
0.003	0.013	0.810	-0.022	0.029	1.003	0.978	1.029
0.006	0.012	0.642	-0.017	0.028	1.006	0.983	1.029
0.000	0.036	0.992	-0.072	0.071	1.000	0.931	1.074
0.001	0.009	0.862	-0.015	0.018	1.001	0.985	1.018
-0.005	0.008	0.579	-0.020	0.011	0.996	0.980	1.011
0.015	0.014	0.313	-0.013	0.042	1.015	0.987	1.043
0.002	0.012	0.872	-0.022	0.026	1.002	0.978	1.026
0.027	0.036	0.508	-0.044	0.098	1.028	0.957	1.103
-0.009	0.013	0.498	-0.035	0.017	0.991	0.965	1.017
-0.001	0.010	0.941	-0.020	0.019	0.999	0.980	1.019
-0.012	0.019	0.573	-0.049	0.025	0.988	0.952	1.026
-0.011	0.018	0.573	-0.048	0.025	0.989	0.954	1.025
0.015	0.030	0.619	-0.044	0.075	1.015	0.957	1.078
0.015	0.007	0.019	0.003	0.028	1.016	1.003	1.029
0.014	0.006	0.032	0.001	0.026	1.014	1.001	1.027
0.020	0.012	0.112	-0.004	0.044	1.020	0.996	1.045
0.018	0.011	0.123	-0.004	0.040	1.018	0.996	1.040
0.008	0.037	0.830	-0.064	0.081	1.008	0.938	1.084
0.005	0.009	0.557	-0.013	0.024	1.005	0.987	1.024
0.004	0.009	0.633	-0.013	0.022	1.004	0.987	1.022
0.016	0.019	0.410	-0.021	0.054	1.017	0.979	1.055
0.018	0.021	0.414	-0.023	0.060	1.018	0.977	1.061
0.447	0.149	0.010	0.154	0.740	1.564	1.167	2.096
0.134	0.072	0.064	-0.008	0.275	1.143	0.992	1.317

0.154	0.055	0.005	0.046	0.262	1.167	1.047	1.300
0.057	0.139	0.688	-0.216	0.329	1.059	0.806	1.390
0.307	0.107	0.012	0.098	0.517	1.360	1.103	1.676
0.043	0.031	0.161	-0.017	0.103	1.044	0.983	1.108
0.023	0.025	0.361	-0.026	0.072	1.023	0.974	1.074
0.048	0.015	0.002	0.018	0.078	1.049	1.018	1.082
0.034	0.057	0.556	-0.078	0.146	1.034	0.925	1.157
0.028	0.030	0.355	-0.031	0.086	1.028	0.970	1.090
0.047	0.036	0.196	-0.024	0.118	1.048	0.977	1.126
0.019	0.023	0.416	-0.027	0.064	1.019	0.974	1.066
0.043	0.017	0.010	0.010	0.076	1.044	1.010	1.079
0.087	0.053	0.104	-0.017	0.191	1.091	0.984	1.211
0.026	0.027	0.336	-0.027	0.079	1.027	0.974	1.083
0.040	0.045	0.375	-0.048	0.129	1.041	0.953	1.138
0.050	0.034	0.141	-0.016	0.115	1.051	0.984	1.122
0.031	0.023	0.180	-0.014	0.076	1.031	0.986	1.079
0.073	0.081	0.370	-0.086	0.231	1.075	0.918	1.260
0.078	0.045	0.089	-0.011	0.167	1.081	0.989	1.181
0.008	0.075	0.913	-0.138	0.155	1.008	0.871	1.167
0.000	0.039	0.990	-0.078	0.077	1.000	0.925	1.080
0.033	0.034	0.319	-0.032	0.099	1.034	0.968	1.104
-0.054	0.087	0.538	-0.224	0.116	0.947	0.799	1.123
-0.061	0.084	0.471	-0.225	0.103	0.941	0.799	1.109
0.143	0.344	0.689	-0.530	0.817	1.154	0.588	2.264
0.155	0.099	0.117	-0.039	0.348	1.167	0.962	1.416
0.101	0.118	0.390	-0.129	0.332	1.106	0.879	1.393
0.355	0.166	0.065	0.029	0.681	1.426	1.029	1.976
0.248	0.152	0.141	-0.049	0.545	1.281	0.952	1.725
0.641	0.274	0.145	0.103	1.179	1.898	1.109	3.250
0.176	0.089	0.049	0.001	0.351	1.192	1.001	1.420
0.191	0.103	0.062	-0.010	0.393	1.211	0.990	1.481
0.168	0.140	0.318	-0.107	0.442	1.183	0.898	1.556
0.165	0.120	0.265	-0.071	0.401	1.179	0.931	1.493
0.450	2.075	0.848	-3.616	4.516	1.568	0.027	91.476
-0.229	0.137	0.095	-0.498	0.040	0.795	0.608	1.040
-0.232	0.140	0.098	-0.506	0.043	0.793	0.603	1.044
-0.307	0.234	0.281	-0.766	0.152	0.735	0.465	1.164
-0.250	0.231	0.358	-0.703	0.203	0.779	0.495	1.225
-0.007	0.046	0.883	-0.097	0.083	0.993	0.907	1.087
0.004	0.012	0.742	-0.019	0.027	1.004	0.981	1.027
0.011	0.011	0.313	-0.010	0.032	1.011	0.990	1.033
0.013	0.019	0.496	-0.023	0.050	1.014	0.977	1.051
-0.002	0.015	0.904	-0.032	0.028	0.998	0.968	1.029
-0.068	0.307	0.828	-0.670	0.534	0.934	0.512	1.705
0.041	0.096	0.670	-0.147	0.229	1.042	0.863	1.258
-0.052	0.096	0.587	-0.240	0.136	0.949	0.787	1.145
0.047	0.157	0.770	-0.260	0.354	1.048	0.771	1.425

0.050	0.146	0.736	-0.235	0.336	1.052	0.790	1.399
0.550	0.256	0.053	0.048	1.052	1.733	1.049	2.864
0.048	0.078	0.540	-0.105	0.201	1.049	0.900	1.222
0.015	0.059	0.804	-0.100	0.129	1.015	0.904	1.138
0.053	0.136	0.705	-0.215	0.320	1.054	0.807	1.377
0.053	0.125	0.681	-0.193	0.299	1.054	0.824	1.348
-0.171	0.204	0.412	-0.571	0.228	0.843	0.565	1.257
-0.031	0.071	0.667	-0.171	0.109	0.970	0.843	1.115
0.002	0.053	0.963	-0.102	0.107	1.002	0.903	1.113
-0.032	0.127	0.803	-0.282	0.217	0.968	0.754	1.243
-0.035	0.111	0.756	-0.252	0.182	0.966	0.777	1.200
0.590	0.558	0.321	-0.502	1.683	1.805	0.605	5.383
0.033	0.113	0.769	-0.189	0.256	1.034	0.828	1.291
-0.077	0.155	0.619	-0.381	0.227	0.926	0.683	1.254
0.058	0.166	0.733	-0.266	0.383	1.060	0.766	1.467
0.047	0.140	0.743	-0.228	0.322	1.049	0.796	1.381
0.049	0.060	0.419	-0.069	0.167	1.050	0.933	1.182
0.038	0.036	0.288	-0.032	0.109	1.039	0.968	1.115
0.008	0.029	0.773	-0.048	0.065	1.008	0.953	1.067
0.036	0.090	0.690	-0.141	0.213	1.037	0.869	1.237
0.056	0.046	0.232	-0.035	0.147	1.057	0.966	1.158
-0.026	0.083	0.760	-0.189	0.138	0.975	0.827	1.148
0.050	0.037	0.186	-0.024	0.123	1.051	0.976	1.131
0.051	0.036	0.152	-0.019	0.121	1.053	0.981	1.129
0.088	0.084	0.303	-0.077	0.253	1.092	0.926	1.288
0.075	0.087	0.401	-0.097	0.246	1.077	0.908	1.279
0.128	0.832	0.883	-1.502	1.759	1.137	0.223	5.806
-0.025	0.124	0.841	-0.268	0.219	0.975	0.765	1.244
-0.038	0.139	0.783	-0.310	0.234	0.963	0.733	1.263
0.041	0.203	0.846	-0.357	0.439	1.042	0.700	1.552
0.019	0.183	0.923	-0.341	0.378	1.019	0.711	1.459
0.113	0.473	0.814	-0.814	1.041	1.120	0.443	2.831
-0.147	0.077	0.055	-0.298	0.003	0.863	0.742	1.003
-0.148	0.076	0.052	-0.297	0.001	0.863	0.743	1.001
-0.219	0.148	0.158	-0.509	0.071	0.803	0.601	1.074
-0.192	0.144	0.202	-0.474	0.091	0.826	0.623	1.095
-0.460	0.517	0.391	-1.473	0.553	0.631	0.229	1.738
-0.093	0.088	0.292	-0.265	0.080	0.912	0.767	1.083
-0.034	0.095	0.721	-0.220	0.152	0.967	0.802	1.165
-0.152	0.152	0.334	-0.449	0.145	0.859	0.638	1.156
-0.138	0.156	0.394	-0.444	0.169	0.871	0.641	1.184
0.123	0.596	0.855	-1.046	1.292	1.131	0.351	3.640
-0.131	0.123	0.289	-0.373	0.111	0.877	0.689	1.118
-0.176	0.104	0.091	-0.380	0.028	0.839	0.684	1.028
-0.061	0.203	0.782	-0.458	0.336	0.941	0.632	1.399
-0.056	0.164	0.754	-0.378	0.265	0.945	0.685	1.304
0.105	0.385	0.798	-0.648	0.859	1.111	0.523	2.361

0.287	0.115	0.013	0.061	0.513	1.333	1.063	1.671
0.292	0.095	0.002	0.107	0.478	1.340	1.113	1.613
0.317	0.181	0.141	-0.038	0.673	1.373	0.963	1.959
0.327	0.183	0.134	-0.032	0.686	1.387	0.969	1.986
0.205	0.422	0.647	-0.622	1.032	1.228	0.537	2.807
0.128	0.109	0.240	-0.086	0.343	1.137	0.918	1.409
0.119	0.112	0.286	-0.100	0.339	1.127	0.905	1.404
0.112	0.164	0.520	-0.209	0.433	1.118	0.812	1.542
0.122	0.153	0.457	-0.179	0.423	1.130	0.836	1.526
1.047	1.463	0.514	-1.821	3.914	2.848	0.162	50.119
0.128	0.164	0.437	-0.194	0.449	1.136	0.824	1.567
0.112	0.243	0.646	-0.365	0.588	1.118	0.694	1.801
0.195	0.274	0.508	-0.341	0.731	1.215	0.711	2.078
0.287	0.262	0.323	-0.226	0.801	1.333	0.798	2.228
-0.008	0.275	0.977	-0.547	0.530	0.992	0.579	1.699
0.063	0.091	0.490	-0.116	0.242	1.065	0.891	1.274
0.063	0.069	0.361	-0.073	0.200	1.065	0.930	1.221
0.038	0.131	0.780	-0.220	0.295	1.039	0.803	1.344
0.057	0.122	0.654	-0.182	0.295	1.058	0.834	1.343
-0.216	0.441	0.633	-1.080	0.649	0.806	0.339	1.914
-0.022	0.095	0.819	-0.209	0.165	0.978	0.812	1.180
0.036	0.099	0.721	-0.159	0.230	1.036	0.853	1.259
-0.025	0.124	0.841	-0.269	0.219	0.975	0.764	1.244
-0.030	0.116	0.801	-0.257	0.197	0.971	0.774	1.218
0.228	0.447	0.645	-0.648	1.104	1.256	0.523	3.016
0.080	0.141	0.570	-0.197	0.357	1.083	0.822	1.429
0.130	0.104	0.213	-0.074	0.334	1.139	0.928	1.396
0.110	0.210	0.630	-0.302	0.522	1.116	0.739	1.685
0.070	0.207	0.753	-0.337	0.477	1.073	0.714	1.611
0.426	0.377	0.272	-0.313	1.165	1.532	0.731	3.207
0.199	0.074	0.007	0.055	0.343	1.220	1.056	1.410
0.167	0.078	0.032	0.014	0.319	1.181	1.014	1.376
0.281	0.143	0.062	0.001	0.561	1.325	1.001	1.752
0.261	0.106	0.023	0.053	0.470	1.299	1.054	1.599
0.216	0.255	0.421	-0.283	0.716	1.241	0.753	2.046
0.299	0.091	0.001	0.121	0.478	1.349	1.128	1.612
0.286	0.064	0.000	0.161	0.412	1.332	1.175	1.509
0.312	0.147	0.063	0.024	0.600	1.366	1.024	1.823
0.304	0.158	0.087	-0.006	0.614	1.355	0.994	1.847
-0.015	0.009	0.119	-0.033	0.003	0.985	0.968	1.003
-0.009	0.003	0.001	-0.015	-0.004	0.991	0.985	0.996
-0.006	0.003	0.052	-0.012	0.000	0.994	0.988	1.000
0.003	0.008	0.737	-0.013	0.019	1.003	0.987	1.019
-0.011	0.004	0.012	-0.019	-0.004	0.989	0.981	0.996
0.000	0.002	0.909	-0.003	0.003	1.000	0.997	1.003
0.000	0.001	0.733	-0.002	0.002	1.000	0.998	1.002
-0.001	0.001	0.096	-0.003	0.000	0.999	0.997	1.000

-0.003	0.002	0.118	-0.007	0.001	0.997	0.993	1.001
-0.001	0.001	0.414	-0.003	0.001	0.999	0.997	1.001
0.000	0.001	0.874	-0.003	0.003	1.000	0.997	1.003
0.000	0.001	0.726	-0.002	0.001	1.000	0.998	1.001
-0.001	0.001	0.177	-0.002	0.000	0.999	0.998	1.000
0.000	0.002	0.919	-0.003	0.003	1.000	0.997	1.003
0.000	0.001	0.592	-0.002	0.001	1.000	0.998	1.001
0.000	0.002	0.989	-0.005	0.005	1.000	0.995	1.005
-0.003	0.001	0.023	-0.005	0.000	0.997	0.995	1.000
-0.002	0.001	0.068	-0.004	0.000	0.998	0.996	1.000
0.000	0.003	0.888	-0.006	0.007	1.000	0.994	1.007
-0.004	0.002	0.081	-0.008	0.000	0.996	0.992	1.000
-0.001	0.003	0.848	-0.007	0.006	0.999	0.993	1.006
0.001	0.001	0.359	-0.002	0.004	1.001	0.998	1.004
-0.001	0.001	0.537	-0.003	0.002	0.999	0.997	1.002
0.003	0.003	0.381	-0.003	0.009	1.003	0.997	1.009
0.003	0.002	0.200	-0.002	0.008	1.003	0.998	1.008
-0.004	0.008	0.655	-0.020	0.012	0.996	0.980	1.012
-0.002	0.004	0.560	-0.009	0.005	0.998	0.991	1.005
-0.004	0.003	0.191	-0.009	0.002	0.996	0.991	1.002
-0.001	0.006	0.918	-0.012	0.010	0.999	0.988	1.011
-0.001	0.005	0.891	-0.010	0.009	0.999	0.990	1.009
-0.009	0.016	0.685	-0.041	0.023	0.991	0.960	1.024
-0.004	0.003	0.207	-0.011	0.002	0.996	0.989	1.002
-0.005	0.003	0.098	-0.010	0.001	0.995	0.990	1.001
-0.002	0.004	0.641	-0.011	0.006	0.998	0.989	1.006
-0.003	0.004	0.523	-0.012	0.005	0.997	0.989	1.005
0.087	0.083	0.402	-0.075	0.249	1.091	0.928	1.283
0.015	0.005	0.003	0.005	0.024	1.015	1.005	1.025
0.014	0.006	0.026	0.002	0.027	1.014	1.002	1.027
0.021	0.010	0.136	0.001	0.041	1.021	1.001	1.042
0.021	0.010	0.125	0.002	0.040	1.021	1.002	1.041
-0.001	0.003	0.840	-0.007	0.005	0.999	0.993	1.005
0.000	0.000	0.937	-0.001	0.001	1.000	0.999	1.001
0.000	0.001	0.903	-0.001	0.001	1.000	0.999	1.001
0.000	0.001	0.482	-0.001	0.002	1.000	0.999	1.002
0.000	0.001	0.492	-0.001	0.001	1.000	0.999	1.001
-0.009	0.014	0.546	-0.036	0.018	0.991	0.965	1.019
-0.005	0.004	0.161	-0.013	0.002	0.995	0.987	1.002
-0.004	0.005	0.373	-0.013	0.005	0.996	0.987	1.005
-0.004	0.005	0.487	-0.014	0.007	0.996	0.986	1.007
-0.005	0.005	0.360	-0.014	0.005	0.995	0.986	1.005
-0.006	0.014	0.672	-0.034	0.022	0.994	0.966	1.022
-0.003	0.003	0.439	-0.009	0.004	0.997	0.991	1.004
-0.003	0.003	0.322	-0.009	0.003	0.997	0.991	1.003
0.003	0.006	0.686	-0.010	0.015	1.003	0.990	1.016
-0.006	0.008	0.440	-0.021	0.009	0.994	0.979	1.009

0.003	0.010	0.759	-0.016	0.022	1.003	0.984	1.022
0.000	0.003	0.890	-0.006	0.005	1.000	0.994	1.005
0.002	0.003	0.355	-0.003	0.007	1.002	0.997	1.007
0.001	0.005	0.871	-0.009	0.010	1.001	0.991	1.010
0.001	0.004	0.779	-0.006	0.009	1.001	0.994	1.009
-0.024	0.013	0.117	-0.049	0.002	0.977	0.952	1.002
-0.004	0.004	0.354	-0.012	0.004	0.996	0.988	1.004
0.000	0.004	0.944	-0.008	0.009	1.000	0.992	1.009
-0.005	0.007	0.462	-0.018	0.008	0.995	0.982	1.008
-0.005	0.006	0.390	-0.017	0.006	0.995	0.983	1.006
-0.003	0.003	0.343	-0.009	0.003	0.997	0.991	1.003
-0.003	0.001	0.018	-0.006	-0.001	0.997	0.994	0.999
0.000	0.001	0.835	-0.003	0.002	1.000	0.997	1.002
-0.005	0.003	0.172	-0.012	0.002	0.995	0.988	1.002
-0.004	0.002	0.034	-0.007	0.000	0.996	0.993	1.000
0.004	0.004	0.327	-0.004	0.011	1.004	0.996	1.011
0.001	0.001	0.602	-0.002	0.004	1.001	0.998	1.004
-0.002	0.001	0.225	-0.005	0.001	0.998	0.995	1.001
0.001	0.003	0.839	-0.006	0.007	1.001	0.994	1.007
0.002	0.002	0.288	-0.002	0.006	1.002	0.998	1.006
-0.063	0.026	0.069	-0.113	-0.013	0.939	0.893	0.987
0.005	0.005	0.283	-0.004	0.014	1.005	0.996	1.014
-0.002	0.007	0.733	-0.015	0.011	0.998	0.985	1.011
0.007	0.005	0.272	-0.004	0.017	1.007	0.996	1.017
0.007	0.006	0.291	-0.004	0.017	1.007	0.996	1.018
-0.011	0.016	0.492	-0.042	0.020	0.989	0.959	1.020
0.000	0.003	0.872	-0.006	0.005	1.000	0.994	1.005
0.002	0.003	0.563	-0.004	0.007	1.002	0.996	1.007
0.000	0.005	0.997	-0.010	0.010	1.000	0.990	1.010
-0.001	0.004	0.897	-0.009	0.008	0.999	0.991	1.008
-0.007	0.025	0.785	-0.056	0.042	0.993	0.946	1.043
0.005	0.004	0.151	-0.002	0.013	1.005	0.998	1.013
0.001	0.005	0.803	-0.008	0.010	1.001	0.992	1.010
0.014	0.007	0.049	0.001	0.027	1.014	1.001	1.027
0.012	0.007	0.119	-0.002	0.027	1.012	0.998	1.027
0.037	0.030	0.341	-0.022	0.096	1.038	0.979	1.101
0.011	0.005	0.043	0.000	0.021	1.011	1.000	1.021
0.010	0.005	0.062	-0.001	0.021	1.010	0.999	1.021
0.011	0.008	0.294	-0.006	0.027	1.011	0.994	1.028
0.010	0.008	0.336	-0.007	0.026	1.010	0.993	1.026
-0.025	0.021	0.304	-0.067	0.017	0.975	0.935	1.017
-0.012	0.004	0.007	-0.021	-0.003	0.988	0.980	0.997
-0.008	0.005	0.110	-0.018	0.002	0.992	0.982	1.002
-0.013	0.006	0.080	-0.024	-0.001	0.987	0.976	0.999
-0.013	0.005	0.064	-0.024	-0.002	0.987	0.977	0.998
-0.020	0.011	0.118	-0.041	0.001	0.980	0.960	1.001
-0.010	0.004	0.010	-0.018	-0.002	0.990	0.983	0.998

-0.008	0.003	0.015	-0.014	-0.002	0.992	0.986	0.998
-0.011	0.006	0.094	-0.023	0.000	0.989	0.978	1.000
-0.012	0.005	0.074	-0.023	-0.001	0.988	0.978	0.999
-0.068	0.028	0.094	-0.124	-0.013	0.934	0.884	0.987
-0.007	0.006	0.286	-0.019	0.006	0.993	0.981	1.006
-0.009	0.008	0.224	-0.024	0.006	0.991	0.976	1.006
0.005	0.011	0.687	-0.018	0.028	1.005	0.983	1.028
0.005	0.016	0.782	-0.027	0.037	1.005	0.973	1.037
0.012	0.021	0.589	-0.029	0.053	1.012	0.971	1.054
-0.003	0.004	0.465	-0.010	0.005	0.997	0.990	1.005
-0.004	0.005	0.353	-0.014	0.005	0.996	0.987	1.005
-0.002	0.006	0.733	-0.013	0.009	0.998	0.987	1.009
-0.002	0.004	0.691	-0.010	0.007	0.998	0.990	1.007
-0.001	0.018	0.957	-0.037	0.035	0.999	0.964	1.036
-0.001	0.004	0.703	-0.008	0.006	0.999	0.992	1.006
-0.004	0.004	0.295	-0.012	0.004	0.996	0.988	1.004
-0.002	0.005	0.717	-0.013	0.009	0.998	0.987	1.009
-0.001	0.005	0.824	-0.010	0.008	0.999	0.990	1.008
0.014	0.018	0.474	-0.020	0.049	1.014	0.980	1.050
0.004	0.005	0.457	-0.006	0.014	1.004	0.994	1.014
0.003	0.005	0.589	-0.007	0.012	1.003	0.993	1.012
0.001	0.008	0.884	-0.014	0.016	1.001	0.986	1.016
0.001	0.007	0.853	-0.013	0.016	1.001	0.987	1.016
0.000	0.014	0.983	-0.028	0.027	1.000	0.973	1.027
-0.004	0.003	0.164	-0.010	0.002	0.996	0.990	1.002
-0.002	0.003	0.558	-0.008	0.004	0.998	0.992	1.004
-0.005	0.006	0.384	-0.017	0.006	0.995	0.983	1.006
-0.003	0.005	0.525	-0.013	0.006	0.997	0.987	1.006
0.001	0.011	0.897	-0.020	0.023	1.001	0.980	1.024
-0.004	0.003	0.264	-0.010	0.003	0.996	0.990	1.003
-0.004	0.003	0.133	-0.009	0.001	0.996	0.991	1.001
-0.004	0.005	0.419	-0.015	0.006	0.996	0.985	1.006
-0.004	0.005	0.404	-0.014	0.005	0.996	0.987	1.005
-0.011	0.043	0.809	-0.095	0.074	0.989	0.909	1.077
-0.006	0.017	0.731	-0.040	0.028	0.994	0.961	1.028
-0.004	0.014	0.798	-0.032	0.025	0.996	0.969	1.025
-0.003	0.027	0.921	-0.056	0.051	0.997	0.945	1.052
-0.004	0.019	0.845	-0.042	0.034	0.996	0.959	1.034
-0.008	0.010	0.393	-0.028	0.011	0.992	0.973	1.011
-0.003	0.006	0.680	-0.014	0.009	0.997	0.986	1.009
0.008	0.005	0.102	-0.002	0.018	1.008	0.998	1.018
0.016	0.012	0.202	-0.008	0.039	1.016	0.992	1.040
0.003	0.007	0.722	-0.012	0.017	1.003	0.988	1.017
-0.008	0.009	0.337	-0.025	0.009	0.992	0.975	1.009
-0.002	0.006	0.697	-0.014	0.010	0.998	0.986	1.010
0.003	0.004	0.535	-0.005	0.011	1.003	0.995	1.011
0.002	0.013	0.891	-0.023	0.027	1.002	0.977	1.027

-0.001	0.006	0.848	-0.014	0.011	0.999	0.986	1.012
-0.002	0.012	0.892	-0.025	0.022	0.998	0.975	1.022
-0.003	0.007	0.690	-0.017	0.011	0.997	0.984	1.011
-0.001	0.006	0.832	-0.014	0.011	0.999	0.986	1.011
-0.022	0.016	0.169	-0.053	0.009	0.978	0.948	1.009
-0.007	0.007	0.318	-0.022	0.007	0.993	0.979	1.007
-0.005	0.015	0.744	-0.034	0.024	0.995	0.966	1.025
-0.008	0.008	0.309	-0.023	0.007	0.992	0.978	1.007
-0.001	0.007	0.897	-0.014	0.013	0.999	0.986	1.013
-0.008	0.015	0.599	-0.036	0.021	0.992	0.964	1.021
-0.008	0.010	0.434	-0.027	0.011	0.992	0.973	1.012
0.001	0.070	0.992	-0.137	0.138	1.001	0.872	1.148
-0.009	0.018	0.590	-0.044	0.025	0.991	0.957	1.025
-0.015	0.025	0.537	-0.063	0.033	0.985	0.939	1.034
-0.011	0.024	0.644	-0.057	0.035	0.989	0.944	1.035
-0.010	0.020	0.641	-0.049	0.030	0.990	0.952	1.030
-0.112	0.055	0.177	-0.219	-0.005	0.894	0.803	0.995
0.005	0.019	0.771	-0.031	0.042	1.005	0.969	1.043
-0.005	0.021	0.793	-0.046	0.035	0.995	0.955	1.036
0.010	0.021	0.657	-0.031	0.051	1.010	0.970	1.052
0.008	0.020	0.707	-0.031	0.047	1.008	0.970	1.048
-0.169	0.495	0.765	-1.140	0.801	0.844	0.320	2.228
-0.022	0.027	0.401	-0.075	0.030	0.978	0.928	1.030
-0.018	0.033	0.589	-0.083	0.047	0.982	0.920	1.048
0.035	0.064	0.621	-0.090	0.161	1.036	0.914	1.174
0.035	0.058	0.590	-0.079	0.150	1.036	0.924	1.162
-0.009	0.007	0.211	-0.022	0.004	0.991	0.978	1.004
0.000	0.002	0.860	-0.005	0.004	1.000	0.995	1.004
-0.001	0.002	0.580	-0.004	0.003	0.999	0.996	1.003
0.001	0.004	0.811	-0.007	0.009	1.001	0.993	1.009
0.001	0.004	0.789	-0.006	0.008	1.001	0.994	1.008
0.010	0.058	0.873	-0.105	0.124	1.010	0.900	1.132
0.003	0.020	0.886	-0.036	0.041	1.003	0.965	1.042
0.008	0.018	0.667	-0.027	0.043	1.008	0.973	1.043
0.006	0.034	0.871	-0.060	0.071	1.006	0.942	1.074
0.007	0.029	0.811	-0.050	0.064	1.007	0.952	1.066
0.017	0.060	0.786	-0.101	0.135	1.017	0.904	1.144
-0.008	0.016	0.643	-0.039	0.024	0.993	0.961	1.025
-0.004	0.013	0.741	-0.030	0.021	0.996	0.971	1.021
0.016	0.030	0.611	-0.044	0.075	1.016	0.957	1.078
0.005	0.026	0.863	-0.047	0.056	1.005	0.954	1.058
-0.101	0.056	0.087	-0.210	0.008	0.904	0.810	1.008
-0.006	0.018	0.714	-0.041	0.028	0.994	0.960	1.028
-0.023	0.016	0.150	-0.054	0.008	0.978	0.948	1.008
0.008	0.037	0.841	-0.065	0.080	1.008	0.937	1.083
0.010	0.041	0.811	-0.070	0.090	1.010	0.933	1.094
0.042	0.059	0.501	-0.074	0.157	1.043	0.929	1.170

0.050	0.021	0.017	0.009	0.092	1.052	1.009	1.096
0.043	0.016	0.008	0.011	0.075	1.044	1.012	1.078
0.070	0.035	0.083	0.001	0.140	1.073	1.001	1.150
0.063	0.034	0.102	-0.004	0.130	1.065	0.996	1.139
0.004	0.012	0.707	-0.018	0.027	1.004	0.982	1.027
-0.002	0.007	0.750	-0.015	0.011	0.998	0.985	1.011
0.005	0.006	0.416	-0.007	0.016	1.005	0.993	1.016
-0.016	0.014	0.267	-0.044	0.012	0.984	0.957	1.012
-0.005	0.008	0.502	-0.020	0.010	0.995	0.980	1.010
0.005	0.016	0.758	-0.026	0.036	1.005	0.974	1.037
-0.009	0.008	0.284	-0.024	0.007	0.991	0.976	1.007
-0.004	0.007	0.568	-0.017	0.010	0.996	0.983	1.010
-0.005	0.015	0.716	-0.034	0.023	0.995	0.967	1.023
-0.007	0.010	0.475	-0.026	0.012	0.993	0.975	1.012
-0.166	0.110	0.191	-0.381	0.049	0.847	0.683	1.051
-0.026	0.026	0.318	-0.076	0.025	0.975	0.927	1.025
-0.030	0.022	0.170	-0.074	0.013	0.970	0.929	1.013
-0.018	0.047	0.713	-0.109	0.073	0.982	0.896	1.076
0.005	0.042	0.903	-0.076	0.087	1.005	0.927	1.091
-0.081	0.072	0.279	-0.222	0.060	0.922	0.801	1.062
-0.006	0.016	0.676	-0.037	0.024	0.994	0.964	1.024
0.002	0.013	0.859	-0.024	0.028	1.002	0.977	1.029
-0.017	0.030	0.586	-0.075	0.042	0.983	0.927	1.043
-0.017	0.026	0.511	-0.068	0.033	0.983	0.935	1.034
-0.143	0.065	0.050	-0.271	-0.015	0.867	0.763	0.986
-0.002	0.018	0.900	-0.037	0.032	0.998	0.964	1.033
0.012	0.015	0.420	-0.017	0.042	1.012	0.983	1.042
-0.030	0.034	0.396	-0.096	0.037	0.971	0.909	1.037
-0.031	0.034	0.383	-0.098	0.036	0.969	0.906	1.037
-0.010	0.247	0.970	-0.495	0.474	0.990	0.610	1.607
-0.008	0.029	0.770	-0.064	0.048	0.992	0.938	1.049
-0.024	0.038	0.528	-0.098	0.050	0.976	0.907	1.051
-0.002	0.042	0.956	-0.084	0.079	0.998	0.919	1.082
-0.003	0.036	0.933	-0.073	0.066	0.997	0.930	1.069
-0.108	0.111	0.402	-0.324	0.109	0.898	0.723	1.115
-0.026	0.030	0.393	-0.085	0.034	0.974	0.918	1.034
0.000	0.029	0.997	-0.057	0.057	1.000	0.945	1.059
-0.036	0.034	0.344	-0.103	0.030	0.964	0.902	1.031
-0.036	0.038	0.398	-0.110	0.038	0.965	0.896	1.039
-0.092	0.062	0.212	-0.213	0.029	0.912	0.808	1.030
-0.016	0.023	0.483	-0.060	0.029	0.984	0.941	1.029
-0.004	0.020	0.839	-0.044	0.035	0.996	0.957	1.036
-0.030	0.037	0.452	-0.102	0.042	0.970	0.903	1.043
-0.031	0.034	0.395	-0.097	0.035	0.969	0.908	1.035
0.053	0.161	0.764	-0.263	0.369	1.054	0.769	1.446
-0.014	0.030	0.642	-0.074	0.045	0.986	0.929	1.046
-0.024	0.028	0.394	-0.080	0.032	0.976	0.923	1.032

-0.013	0.042	0.780	-0.095	0.070	0.987	0.909	1.073
-0.015	0.042	0.739	-0.097	0.067	0.985	0.908	1.069
0.154	0.097	0.157	-0.036	0.343	1.166	0.964	1.410
0.038	0.024	0.111	-0.009	0.085	1.039	0.991	1.089
0.035	0.026	0.188	-0.017	0.086	1.035	0.983	1.090
0.056	0.052	0.312	-0.045	0.157	1.057	0.956	1.170
0.073	0.040	0.108	-0.006	0.151	1.075	0.994	1.163
-0.188	0.081	0.038	-0.346	-0.030	0.829	0.708	0.971
-0.061	0.021	0.003	-0.102	-0.021	0.940	0.903	0.979
-0.033	0.022	0.135	-0.077	0.010	0.967	0.926	1.010
-0.073	0.040	0.092	-0.151	0.006	0.930	0.860	1.006
-0.075	0.032	0.036	-0.137	-0.012	0.928	0.872	0.988
0.033	0.065	0.645	-0.095	0.161	1.034	0.910	1.175
0.004	0.027	0.880	-0.048	0.056	1.004	0.953	1.058
0.008	0.022	0.699	-0.034	0.051	1.008	0.966	1.052
0.008	0.036	0.827	-0.062	0.078	1.008	0.940	1.081
0.004	0.036	0.920	-0.067	0.075	1.004	0.935	1.078
0.002	0.059	0.972	-0.113	0.117	1.002	0.893	1.124
0.002	0.015	0.890	-0.028	0.032	1.002	0.972	1.033
-0.009	0.012	0.459	-0.033	0.015	0.991	0.967	1.015
0.015	0.025	0.557	-0.034	0.065	1.015	0.966	1.067
0.009	0.024	0.721	-0.038	0.056	1.009	0.962	1.057
-0.069	0.058	0.272	-0.183	0.046	0.933	0.833	1.047
0.000	0.018	0.988	-0.035	0.034	1.000	0.966	1.035
0.004	0.016	0.798	-0.028	0.036	1.004	0.972	1.037
-0.003	0.028	0.906	-0.059	0.052	0.997	0.943	1.053
-0.001	0.025	0.972	-0.050	0.049	0.999	0.951	1.050
