# Supplementary material for: Causal Links Between Renal Function and Cardiac Structure, Function, and Disease Risk
Source: Glob Heart. 2024 Nov 6;19(1):83. doi: 10.5334/gh.1366 (PMC11546326; doi:10.5334/gh.1366)
Supplement: Table S6. — Test results for pleiotropy and heterogeneity – causal effects of the cardiovascular diseases on renal function. [file gh-19-1-1366-s10.pdf]

Table S6. Test results for pleiotropy and heterogeneity - causal effects of the cardiova

Exposures	Outcome	Cochran's Q test		MR-Egger	
		Q	*P* value	Egger_ intercept	*P* value
Stroke	CKD	62.11	0.00	-0.0141	0.49
Stroke finngen	CKD	9.16	0.42	0.0046	0.78
Atrial fibrillation	CKD	102.97	0.35	0.0004	0.85
Atrial fibrillation finnge	CKD	103.78	0.01	-0.0005	0.90

ascular diseases on Renal function.

MR-PRESSO	MR-PRESSO Outlier-corrected	
global test	*Beta*	*P* value
<0.001	0.15	0.045
0.46	NA	NA
0.34	NA	NA
0.01	NA	NA
