# Supplementary material for: Causal Links Between Renal Function and Cardiac Structure, Function, and Disease Risk
Source: Glob Heart. 2024 Nov 6;19(1):83. doi: 10.5334/gh.1366 (PMC11546326; doi:10.5334/gh.1366)
Supplement: Table S7. — Multivariable MR analysis of the causal effects after adjusting for risk factors. [file gh-19-1-1366-s11.pdf]

Table S7. Multivariable mendelian randomization analysis of the causal eff

Exposure	Outcome	method	b	lo_ci
BUN adj.Confound	Coronary artery disease	Inverse variance weighte	0.225	0.181
UACR adj.Confound	Coronary artery disease	Inverse variance weighte	0.000	0.093
UACR adj.Confound	Myocardial infarction	Inverse variance weighte	-0.091	0.130
UACR adj.Confound	Stroke	Inverse variance weighte	0.243	0.103
Stroke adj.Confound	Chronic Kidney Disease	Inverse variance weighte	0.079	0.058
AF adj.Confound	Chronic Kidney Disease	Inverse variance weighte	0.028	0.021
BUN adj.Confound	LVEF	Inverse variance weighte	-0.201	0.164
BUN adj.Confound	Prox PA Diam Indexed	Inverse variance weighte	-0.349	0.201
CKD adj.Confound	PA Aorta ratio	Inverse variance weighte	-0.026	0.026
CKD adj.Confound	Prox PA Diam Indexed	Inverse variance weighte	-0.058	0.026
eGFR adj.Confound	Asc Aorta Diam Indexed	Inverse variance weighte	0.893	0.278
eGFR adj.Confound	LVSV Indexed	Inverse variance weighte	0.634	0.251
eGFR adj.Confound	Prox PA Diam Indexed	Inverse variance weighte	1.192	0.295
eGFR adj.Confound	RA Max Indexed	Inverse variance weighte	0.763	0.254
eGFR adj.Confound	RA Min Indexed	Inverse variance weighte	0.723	0.268
eGFR adj.Confound	RVEDV Indexed	Inverse variance weighte	0.444	0.243
eGFR adj.Confound	RVSV Indexed	Inverse variance weighte	0.498	0.247

fects after adjusting for risk factors.

up_ci	se	pval	or	or_lci95	or_uci95
0.214	-0.129	0.579	1.252	0.879	1.784
0.996	-0.182	0.183	1.000	0.833	1.201
0.482	-0.345	0.163	0.913	0.708	1.177
0.018	0.041	0.444	1.275	1.042	1.559
0.168	-0.033	0.192	1.083	0.967	1.212
0.189	-0.014	0.069	1.028	0.986	1.072
0.221	-0.523	0.121	0.818	0.593	1.129
0.083	-0.743	0.045	0.705	0.476	1.046
0.326	-0.077	0.025	0.975	0.926	1.026
0.027	-0.110	-0.007	0.943	0.896	0.993
0.001	0.347	1.438	2.441	1.415	4.213
0.012	0.141	1.127	1.886	1.152	3.087
0.000	0.614	1.769	3.292	1.848	5.864
0.003	0.265	1.261	2.145	1.304	3.530
0.007	0.198	1.249	2.061	1.219	3.486
0.067	-0.032	0.920	1.559	0.969	2.509
0.044	0.014	0.982	1.645	1.014	2.669
