# Supplementary material for: Causal Links Between Renal Function and Cardiac Structure, Function, and Disease Risk
Source: Glob Heart. 2024 Nov 6;19(1):83. doi: 10.5334/gh.1366 (PMC11546326; doi:10.5334/gh.1366)
Supplement: Table S8. — Effect estimates of the renal function on cardiovascular magnetic resonance imaging parameters of cardiac structure and function. [file gh-19-1-1366-s12.pdf]

Table S8. Effect estimates of the Renal function on cardiovascular magnetic resonance imagi structure and function

Exposure	Outcome	method	No. of SNP	b	se
BUN	LVEF	MR Egger	63	-0.177	0.458
BUN	LVEF	Weighted median	63	-0.403	0.185
BUN	LVEF	Inverse variance weighted	63	-0.400	0.149
BUN	LVEF	Simple mode	63	-0.514	0.390
BUN	LVEF	Weighted mode	63	-0.364	0.302
BUN	Prox PA Diam Indexed	MR Egger	62	-0.117	0.462
BUN	Prox PA Diam Indexed	Weighted median	62	-0.482	0.192
BUN	Prox PA Diam Indexed	Inverse variance weighted	62	-0.381	0.152
BUN	Prox PA Diam Indexed	Simple mode	62	-0.443	0.475
BUN	Prox PA Diam Indexed	Weighted mode	62	-0.580	0.341
BUN	Asc Aorta Diam Indexed	MR Egger	62	-0.182	0.534
BUN	Asc Aorta Diam Indexed	Weighted median	62	-0.129	0.203
BUN	Asc Aorta Diam Indexed	Inverse variance weighted	62	-0.191	0.175
BUN	Asc Aorta Diam Indexed	Simple mode	62	-0.003	0.440
BUN	Asc Aorta Diam Indexed	Weighted mode	62	-0.066	0.379
BUN	LA Max Indexed	MR Egger	63	-0.023	0.528
BUN	LA Max Indexed	Weighted median	63	-0.238	0.221
BUN	LA Max Indexed	Inverse variance weighted	63	-0.107	0.172
BUN	LA Max Indexed	Simple mode	63	-0.758	0.560
BUN	LA Max Indexed	Weighted mode	63	-0.558	0.448
BUN	LATEF	MR Egger	63	0.474	0.505
BUN	LATEF	Weighted median	63	0.268	0.213
BUN	LATEF	Inverse variance weighted	63	0.034	0.166
BUN	LATEF	Simple mode	63	0.161	0.407
BUN	LATEF	Weighted mode	63	0.199	0.339
BUN	LV Mass Indexed	MR Egger	61	5.526	5.931
BUN	LV Mass Indexed	Weighted median	61	2.769	2.123
BUN	LV Mass Indexed	Inverse variance weighted	61	2.494	1.928
BUN	LV Mass Indexed	Simple mode	61	-1.708	4.239
BUN	LV Mass Indexed	Weighted mode	61	1.838	3.171
BUN	LVEDV Indexed	MR Egger	62	0.799	0.461
BUN	LVEDV Indexed	Weighted median	62	0.196	0.172
BUN	LVEDV Indexed	Inverse variance weighted	62	0.076	0.154
BUN	LVEDV Indexed	Simple mode	62	-0.113	0.355
BUN	LVEDV Indexed	Weighted mode	62	0.216	0.279
BUN	LVESV Indexed	MR Egger	62	0.723	0.492
BUN	LVESV Indexed	Weighted median	62	0.441	0.177
BUN	LVESV Indexed	Inverse variance weighted	62	0.293	0.162
BUN	LVESV Indexed	Simple mode	62	0.502	0.362
BUN	LVESV Indexed	Weighted mode	62	0.386	0.289
BUN	LVSV Indexed	MR Egger	62	0.716	0.411
BUN	LVSV Indexed	Weighted median	62	-0.013	0.185

BUN	LVSV Indexed	Inverse variance weighted	62	-0.164	0.140
BUN	LVSV Indexed	Simple mode	62	0.073	0.381
BUN	LVSV Indexed	Weighted mode	62	0.093	0.277
BUN	Myocardial interstitial fibros	MR Egger	63	0.049	0.416
BUN	Myocardial interstitial fibros	Weighted median	63	0.209	0.182
BUN	Myocardial interstitial fibros	Inverse variance weighted	63	0.214	0.135
BUN	Myocardial interstitial fibros	Simple mode	63	0.240	0.400
BUN	Myocardial interstitial fibros	Weighted mode	63	0.161	0.301
BUN	PA Aorta ratio	MR Egger	62	-0.148	0.507
BUN	PA Aorta ratio	Weighted median	62	-0.412	0.210
BUN	PA Aorta ratio	Inverse variance weighted	62	-0.319	0.166
BUN	PA Aorta ratio	Simple mode	62	-0.536	0.462
BUN	PA Aorta ratio	Weighted mode	62	-0.557	0.371
BUN	RA FAC	MR Egger	63	-0.080	0.552
BUN	RA FAC	Weighted median	63	0.323	0.209
BUN	RA FAC	Inverse variance weighted	63	0.115	0.179
BUN	RA FAC	Simple mode	63	0.557	0.461
BUN	RA FAC	Weighted mode	63	0.314	0.350
BUN	RA Max Indexed	MR Egger	63	0.080	0.511
BUN	RA Max Indexed	Weighted median	63	-0.081	0.204
BUN	RA Max Indexed	Inverse variance weighted	63	-0.211	0.166
BUN	RA Max Indexed	Simple mode	63	0.343	0.478
BUN	RA Max Indexed	Weighted mode	63	0.210	0.412
BUN	RA Min Indexed	MR Egger	62	0.086	0.529
BUN	RA Min Indexed	Weighted median	62	-0.255	0.196
BUN	RA Min Indexed	Inverse variance weighted	62	-0.292	0.173
BUN	RA Min Indexed	Simple mode	62	-0.172	0.398
BUN	RA Min Indexed	Weighted mode	62	-0.194	0.309
BUN	RVEDV Indexed	MR Egger	62	0.820	0.441
BUN	RVEDV Indexed	Weighted median	62	-0.145	0.174
BUN	RVEDV Indexed	Inverse variance weighted	62	0.026	0.148
BUN	RVEDV Indexed	Simple mode	62	-0.351	0.382
BUN	RVEDV Indexed	Weighted mode	62	-0.280	0.315
BUN	RVEF	MR Egger	63	-0.075	0.457
BUN	RVEF	Weighted median	63	-0.420	0.190
BUN	RVEF	Inverse variance weighted	63	-0.174	0.148
BUN	RVEF	Simple mode	63	-0.720	0.416
BUN	RVEF	Weighted mode	63	-0.649	0.367
BUN	RVESV Indexed	MR Egger	63	0.706	0.490
BUN	RVESV Indexed	Weighted median	63	0.255	0.180
BUN	RVESV Indexed	Inverse variance weighted	63	0.147	0.161
BUN	RVESV Indexed	Simple mode	63	0.386	0.422
BUN	RVESV Indexed	Weighted mode	63	0.343	0.381
BUN	RVESV LVESV ratio	MR Egger	63	0.433	0.489
BUN	RVESV LVESV ratio	Weighted median	63	-0.201	0.199
BUN	RVESV LVESV ratio	Inverse variance weighted	63	-0.130	0.161
BUN	RVESV LVESV ratio	Simple mode	63	-0.208	0.462
BUN	RVESV LVESV ratio	Weighted mode	63	-0.477	0.408

BUN	RVSV Indexed	MR Egger	62	0.684	0.419
BUN	RVSV Indexed	Weighted median	62	-0.106	0.189
BUN	RVSV Indexed	Inverse variance weighted	62	-0.069	0.141
BUN	RVSV Indexed	Simple mode	62	-0.205	0.461
BUN	RVSV Indexed	Weighted mode	62	-0.124	0.356
CKD	PA Aorta ratio	MR Egger	19	-0.053	0.054
CKD	PA Aorta ratio	Weighted median	19	-0.095	0.032
CKD	PA Aorta ratio	Inverse variance weighted	19	-0.086	0.023
CKD	PA Aorta ratio	Simple mode	19	-0.088	0.049
CKD	PA Aorta ratio	Weighted mode	19	-0.097	0.036
CKD	Prox PA Diam Indexed	MR Egger	20	-0.054	0.050
CKD	Prox PA Diam Indexed	Weighted median	20	-0.062	0.030
CKD	Prox PA Diam Indexed	Inverse variance weighted	20	-0.053	0.021
CKD	Prox PA Diam Indexed	Simple mode	20	-0.081	0.051
CKD	Prox PA Diam Indexed	Weighted mode	20	-0.070	0.037
CKD	Asc Aorta Diam Indexed	MR Egger	20	0.017	0.060
CKD	Asc Aorta Diam Indexed	Weighted median	20	0.006	0.030
CKD	Asc Aorta Diam Indexed	Inverse variance weighted	20	-0.014	0.024
CKD	Asc Aorta Diam Indexed	Simple mode	20	0.008	0.055
CKD	Asc Aorta Diam Indexed	Weighted mode	20	0.006	0.034
CKD	LA Max Indexed	MR Egger	20	-0.109	0.061
CKD	LA Max Indexed	Weighted median	20	-0.016	0.033
CKD	LA Max Indexed	Inverse variance weighted	20	-0.021	0.026
CKD	LA Max Indexed	Simple mode	20	0.072	0.073
CKD	LA Max Indexed	Weighted mode	20	-0.094	0.037
CKD	LATEF	MR Egger	20	0.063	0.062
CKD	LATEF	Weighted median	20	0.019	0.033
CKD	LATEF	Inverse variance weighted	20	-0.002	0.026
CKD	LATEF	Simple mode	20	-0.088	0.063
CKD	LATEF	Weighted mode	20	0.019	0.037
CKD	LV Mass Indexed	MR Egger	18	0.408	0.610
CKD	LV Mass Indexed	Weighted median	18	0.191	0.333
CKD	LV Mass Indexed	Inverse variance weighted	18	-0.059	0.238
CKD	LV Mass Indexed	Simple mode	18	-0.871	0.642
CKD	LV Mass Indexed	Weighted mode	18	0.367	0.394
CKD	LVEDV Indexed	MR Egger	20	0.029	0.057
CKD	LVEDV Indexed	Weighted median	20	-0.009	0.030
CKD	LVEDV Indexed	Inverse variance weighted	20	-0.026	0.024
CKD	LVEDV Indexed	Simple mode	20	-0.006	0.051
CKD	LVEDV Indexed	Weighted mode	20	-0.001	0.033
CKD	LVEF	MR Egger	20	-0.035	0.049
CKD	LVEF	Weighted median	20	-0.045	0.029
CKD	LVEF	Inverse variance weighted	20	-0.018	0.020
CKD	LVEF	Simple mode	20	-0.030	0.055
CKD	LVEF	Weighted mode	20	-0.044	0.032
CKD	LVESV Indexed	MR Egger	20	0.048	0.055
CKD	LVESV Indexed	Weighted median	20	0.018	0.028
CKD	LVESV Indexed	Inverse variance weighted	20	-0.006	0.023

CKD	LVESV Indexed	Simple mode	20	0.010	0.047
CKD	LVESV Indexed	Weighted mode	20	0.027	0.030
CKD	LVSV Indexed	MR Egger	20	0.004	0.052
CKD	LVSV Indexed	Weighted median	20	-0.040	0.030
CKD	LVSV Indexed	Inverse variance weighted	20	-0.037	0.022
CKD	LVSV Indexed	Simple mode	20	-0.040	0.052
CKD	LVSV Indexed	Weighted mode	20	-0.040	0.036
CKD	Myocardial interstitial fibros	MR Egger	19	0.055	0.050
CKD	Myocardial interstitial fibros	Weighted median	19	0.052	0.027
CKD	Myocardial interstitial fibros	Inverse variance weighted	19	0.029	0.020
CKD	Myocardial interstitial fibros	Simple mode	19	0.072	0.050
CKD	Myocardial interstitial fibros	Weighted mode	19	0.064	0.033
CKD	RA FAC	MR Egger	20	0.100	0.066
CKD	RA FAC	Weighted median	20	0.038	0.032
CKD	RA FAC	Inverse variance weighted	20	0.004	0.028
CKD	RA FAC	Simple mode	20	0.029	0.060
CKD	RA FAC	Weighted mode	20	0.043	0.040
CKD	RA Max Indexed	MR Egger	20	-0.051	0.067
CKD	RA Max Indexed	Weighted median	20	-0.022	0.033
CKD	RA Max Indexed	Inverse variance weighted	20	0.006	0.028
CKD	RA Max Indexed	Simple mode	20	0.082	0.069
CKD	RA Max Indexed	Weighted mode	20	-0.028	0.037
CKD	RA Min Indexed	MR Egger	20	-0.087	0.076
CKD	RA Min Indexed	Weighted median	20	-0.027	0.032
CKD	RA Min Indexed	Inverse variance weighted	20	0.005	0.032
CKD	RA Min Indexed	Simple mode	20	-0.012	0.071
CKD	RA Min Indexed	Weighted mode	20	-0.029	0.036
CKD	RVEDV Indexed	MR Egger	20	0.017	0.049
CKD	RVEDV Indexed	Weighted median	20	-0.036	0.027
CKD	RVEDV Indexed	Inverse variance weighted	20	-0.027	0.020
CKD	RVEDV Indexed	Simple mode	20	-0.028	0.048
CKD	RVEDV Indexed	Weighted mode	20	-0.031	0.030
CKD	RVEF	MR Egger	20	0.011	0.051
CKD	RVEF	Weighted median	20	-0.018	0.030
CKD	RVEF	Inverse variance weighted	20	-0.018	0.021
CKD	RVEF	Simple mode	20	0.035	0.056
CKD	RVEF	Weighted mode	20	0.000	0.036
CKD	RVESV Indexed	MR Egger	20	0.009	0.044
CKD	RVESV Indexed	Weighted median	20	-0.013	0.026
CKD	RVESV Indexed	Inverse variance weighted	20	-0.006	0.018
CKD	RVESV Indexed	Simple mode	20	-0.032	0.041
CKD	RVESV Indexed	Weighted mode	20	-0.022	0.032
CKD	RVESV LVESV ratio	MR Egger	20	0.022	0.071
CKD	RVESV LVESV ratio	Weighted median	20	-0.025	0.033
CKD	RVESV LVESV ratio	Inverse variance weighted	20	-0.008	0.029
CKD	RVESV LVESV ratio	Simple mode	20	-0.077	0.066
CKD	RVESV LVESV ratio	Weighted mode	20	-0.038	0.037
CKD	RVSV Indexed	MR Egger	20	0.012	0.059

CKD	RVSV Indexed	Weighted median	20	-0.036	0.030
CKD	RVSV Indexed	Inverse variance weighted	20	-0.040	0.024
CKD	RVSV Indexed	Simple mode	20	-0.011	0.053
CKD	RVSV Indexed	Weighted mode	20	-0.031	0.035
eGFR	Asc Aorta Diam Indexed	MR Egger	172	0.818	0.573
eGFR	Asc Aorta Diam Indexed	Weighted median	172	0.370	0.297
eGFR	Asc Aorta Diam Indexed	Inverse variance weighted	172	0.716	0.225
eGFR	Asc Aorta Diam Indexed	Simple mode	172	0.521	0.769
eGFR	Asc Aorta Diam Indexed	Weighted mode	172	0.327	0.596
eGFR	LA Max Indexed	MR Egger	170	-0.023	0.594
eGFR	LA Max Indexed	Weighted median	170	0.033	0.318
eGFR	LA Max Indexed	Inverse variance weighted	170	0.519	0.234
eGFR	LA Max Indexed	Simple mode	170	-0.239	0.957
eGFR	LA Max Indexed	Weighted mode	170	-0.183	0.743
eGFR	LV Mass Indexed	MR Egger	171	0.495	6.815
eGFR	LV Mass Indexed	Weighted median	171	6.997	3.214
eGFR	LV Mass Indexed	Inverse variance weighted	171	6.069	2.683
eGFR	LV Mass Indexed	Simple mode	171	14.932	9.540
eGFR	LV Mass Indexed	Weighted mode	171	2.868	7.467
eGFR	LVEDV Indexed	MR Egger	171	0.318	0.581
eGFR	LVEDV Indexed	Weighted median	171	0.317	0.292
eGFR	LVEDV Indexed	Inverse variance weighted	171	0.504	0.227
eGFR	LVEDV Indexed	Simple mode	171	0.549	0.892
eGFR	LVEDV Indexed	Weighted mode	171	0.163	0.675
eGFR	LVSV Indexed	MR Egger	170	0.827	0.547
eGFR	LVSV Indexed	Weighted median	170	0.859	0.305
eGFR	LVSV Indexed	Inverse variance weighted	170	0.676	0.214
eGFR	LVSV Indexed	Simple mode	170	1.190	0.834
eGFR	LVSV Indexed	Weighted mode	170	1.035	0.603
eGFR	Prox PA Diam Indexed	MR Egger	172	1.390	0.551
eGFR	Prox PA Diam Indexed	Weighted median	172	1.200	0.295
eGFR	Prox PA Diam Indexed	Inverse variance weighted	172	0.961	0.216
eGFR	Prox PA Diam Indexed	Simple mode	172	0.907	0.703
eGFR	Prox PA Diam Indexed	Weighted mode	172	1.196	0.452
eGFR	RA Max Indexed	MR Egger	171	0.092	0.534
eGFR	RA Max Indexed	Weighted median	171	0.703	0.308
eGFR	RA Max Indexed	Inverse variance weighted	171	0.714	0.205
eGFR	RA Max Indexed	Simple mode	171	1.775	0.811
eGFR	RA Max Indexed	Weighted mode	171	0.683	0.578
eGFR	RA Min Indexed	MR Egger	172	0.505	0.563
eGFR	RA Min Indexed	Weighted median	172	0.764	0.309
eGFR	RA Min Indexed	Inverse variance weighted	172	0.783	0.221
eGFR	RA Min Indexed	Simple mode	172	0.558	0.815
eGFR	RA Min Indexed	Weighted mode	172	0.461	0.620
eGFR	RVEDV Indexed	MR Egger	170	0.513	0.571
eGFR	RVEDV Indexed	Weighted median	170	0.408	0.287
eGFR	RVEDV Indexed	Inverse variance weighted	170	0.625	0.222
eGFR	RVEDV Indexed	Simple mode	170	-0.285	0.729

eGFR	RVEDV Indexed	Weighted mode	170	0.261	0.566
eGFR	RVSV Indexed	MR Egger	171	0.692	0.604
eGFR	RVSV Indexed	Weighted median	171	0.396	0.306
eGFR	RVSV Indexed	Inverse variance weighted	171	0.593	0.236
eGFR	RVSV Indexed	Simple mode	171	-0.299	0.841
eGFR	RVSV Indexed	Weighted mode	171	0.102	0.624
eGFR	LATEF	MR Egger	172	0.522	0.535
eGFR	LATEF	Weighted median	172	-0.070	0.316
eGFR	LATEF	Inverse variance weighted	172	0.095	0.210
eGFR	LATEF	Simple mode	172	0.501	0.948
eGFR	LATEF	Weighted mode	172	-0.234	0.612
eGFR	LVEF	MR Egger	171	1.135	0.578
eGFR	LVEF	Weighted median	171	0.176	0.296
eGFR	LVEF	Inverse variance weighted	171	0.103	0.229
eGFR	LVEF	Simple mode	171	-0.271	0.713
eGFR	LVEF	Weighted mode	171	0.324	0.513
eGFR	LVESV Indexed	MR Egger	170	-1.044	0.604
eGFR	LVESV Indexed	Weighted median	170	-0.438	0.287
eGFR	LVESV Indexed	Inverse variance weighted	170	0.067	0.233
eGFR	LVESV Indexed	Simple mode	170	1.722	0.936
eGFR	LVESV Indexed	Weighted mode	170	-1.032	0.630
eGFR	Myocardial interstitial fibros	MR Egger	172	-0.265	0.486
eGFR	Myocardial interstitial fibros	Weighted median	172	-0.266	0.272
eGFR	Myocardial interstitial fibros	Inverse variance weighted	172	0.001	0.190
eGFR	Myocardial interstitial fibros	Simple mode	172	-0.265	0.672
eGFR	Myocardial interstitial fibros	Weighted mode	172	-0.524	0.451
eGFR	PA Aorta ratio	MR Egger	170	0.794	0.598
eGFR	PA Aorta ratio	Weighted median	170	0.433	0.329
eGFR	PA Aorta ratio	Inverse variance weighted	170	0.382	0.235
eGFR	PA Aorta ratio	Simple mode	170	0.226	0.887
eGFR	PA Aorta ratio	Weighted mode	170	0.427	0.628
eGFR	RA FAC	MR Egger	171	0.085	0.579
eGFR	RA FAC	Weighted median	171	-0.371	0.314
eGFR	RA FAC	Inverse variance weighted	171	-0.261	0.227
eGFR	RA FAC	Simple mode	171	-0.630	0.759
eGFR	RA FAC	Weighted mode	171	-0.683	0.528
eGFR	RVEF	MR Egger	171	0.915	0.504
eGFR	RVEF	Weighted median	171	0.350	0.290
eGFR	RVEF	Inverse variance weighted	171	0.014	0.200
eGFR	RVEF	Simple mode	171	0.532	0.757
eGFR	RVEF	Weighted mode	171	0.621	0.491
eGFR	RVESV Indexed	MR Egger	171	-0.317	0.538
eGFR	RVESV Indexed	Weighted median	171	0.286	0.278
eGFR	RVESV Indexed	Inverse variance weighted	171	0.315	0.211
eGFR	RVESV Indexed	Simple mode	171	0.261	0.695
eGFR	RVESV Indexed	Weighted mode	171	0.474	0.493
eGFR	RVESV LVESV ratio	MR Egger	172	-0.404	0.591
eGFR	RVESV LVESV ratio	Weighted median	172	0.400	0.323

eGFR	RVESV LVESV ratio	Inverse variance weighted	172	0.075	0.232
eGFR	RVESV LVESV ratio	Simple mode	172	0.952	0.814
eGFR	RVESV LVESV ratio	Weighted mode	172	0.617	0.589
UACR	RA Max Indexed	MR Egger	51	-0.030	0.160
UACR	RA Max Indexed	Weighted median	51	-0.019	0.110
UACR	RA Max Indexed	Inverse variance weighted	51	0.163	0.078
UACR	RA Max Indexed	Simple mode	51	-0.020	0.233
UACR	RA Max Indexed	Weighted mode	51	-0.020	0.128
UACR	RA Min Indexed	MR Egger	51	0.026	0.171
UACR	RA Min Indexed	Weighted median	51	0.015	0.114
UACR	RA Min Indexed	Inverse variance weighted	51	0.194	0.083
UACR	RA Min Indexed	Simple mode	51	0.327	0.251
UACR	RA Min Indexed	Weighted mode	51	0.049	0.134
UACR	Asc Aorta Diam Indexed	MR Egger	51	0.021	0.155
UACR	Asc Aorta Diam Indexed	Weighted median	51	-0.026	0.101
UACR	Asc Aorta Diam Indexed	Inverse variance weighted	51	0.105	0.074
UACR	Asc Aorta Diam Indexed	Simple mode	51	0.129	0.201
UACR	Asc Aorta Diam Indexed	Weighted mode	51	-0.021	0.121
UACR	LA Max Indexed	MR Egger	51	-0.194	0.158
UACR	LA Max Indexed	Weighted median	51	-0.066	0.106
UACR	LA Max Indexed	Inverse variance weighted	51	0.110	0.080
UACR	LA Max Indexed	Simple mode	51	0.459	0.282
UACR	LA Max Indexed	Weighted mode	51	-0.145	0.145
UACR	LATEF	MR Egger	51	0.008	0.140
UACR	LATEF	Weighted median	51	-0.050	0.118
UACR	LATEF	Inverse variance weighted	51	-0.077	0.068
UACR	LATEF	Simple mode	51	-0.319	0.214
UACR	LATEF	Weighted mode	51	-0.108	0.135
UACR	LV Mass Indexed	MR Egger	50	0.958	2.196
UACR	LV Mass Indexed	Weighted median	50	1.735	1.219
UACR	LV Mass Indexed	Inverse variance weighted	50	1.686	1.012
UACR	LV Mass Indexed	Simple mode	50	4.487	2.260
UACR	LV Mass Indexed	Weighted mode	50	2.131	1.352
UACR	LVEDV Indexed	MR Egger	51	0.062	0.175
UACR	LVEDV Indexed	Weighted median	51	0.055	0.098
UACR	LVEDV Indexed	Inverse variance weighted	51	0.114	0.084
UACR	LVEDV Indexed	Simple mode	51	0.205	0.174
UACR	LVEDV Indexed	Weighted mode	51	0.084	0.112
UACR	LVEF	MR Egger	51	-0.128	0.145
UACR	LVEF	Weighted median	51	-0.069	0.107
UACR	LVEF	Inverse variance weighted	51	0.016	0.070
UACR	LVEF	Simple mode	51	-0.032	0.219
UACR	LVEF	Weighted mode	51	-0.083	0.121
UACR	LVESV Indexed	MR Egger	51	0.120	0.179
UACR	LVESV Indexed	Weighted median	51	0.081	0.105
UACR	LVESV Indexed	Inverse variance weighted	51	0.060	0.086
UACR	LVESV Indexed	Simple mode	51	-0.047	0.181
UACR	LVESV Indexed	Weighted mode	51	0.086	0.111

UACR	LVSV Indexed	MR Egger	51	0.018	0.147
UACR	LVSV Indexed	Weighted median	51	0.128	0.096
UACR	LVSV Indexed	Inverse variance weighted	51	0.135	0.071
UACR	LVSV Indexed	Simple mode	51	0.330	0.217
UACR	LVSV Indexed	Weighted mode	51	0.083	0.124
UACR	Myocardial interstitial fibros	MR Egger	50	-0.083	0.132
UACR	Myocardial interstitial fibros	Weighted median	50	-0.097	0.092
UACR	Myocardial interstitial fibros	Inverse variance weighted	50	-0.002	0.061
UACR	Myocardial interstitial fibros	Simple mode	50	-0.032	0.213
UACR	Myocardial interstitial fibros	Weighted mode	50	-0.122	0.134
UACR	PA Aorta ratio	MR Egger	51	0.268	0.162
UACR	PA Aorta ratio	Weighted median	51	0.130	0.113
UACR	PA Aorta ratio	Inverse variance weighted	51	0.043	0.079
UACR	PA Aorta ratio	Simple mode	51	-0.090	0.256
UACR	PA Aorta ratio	Weighted mode	51	0.202	0.144
UACR	Prox PA Diam Indexed	MR Egger	51	0.169	0.144
UACR	Prox PA Diam Indexed	Weighted median	51	0.073	0.111
UACR	Prox PA Diam Indexed	Inverse variance weighted	51	0.080	0.069
UACR	Prox PA Diam Indexed	Simple mode	51	0.181	0.190
UACR	Prox PA Diam Indexed	Weighted mode	51	0.128	0.122
UACR	RA FAC	MR Egger	51	-0.097	0.155
UACR	RA FAC	Weighted median	51	-0.091	0.115
UACR	RA FAC	Inverse variance weighted	51	-0.123	0.074
UACR	RA FAC	Simple mode	51	-0.347	0.228
UACR	RA FAC	Weighted mode	51	-0.107	0.127
UACR	RVEDV Indexed	MR Egger	50	0.058	0.149
UACR	RVEDV Indexed	Weighted median	50	0.004	0.098
UACR	RVEDV Indexed	Inverse variance weighted	50	0.119	0.072
UACR	RVEDV Indexed	Simple mode	50	0.057	0.200
UACR	RVEDV Indexed	Weighted mode	50	-0.004	0.124
UACR	RVEF	MR Egger	51	-0.140	0.151
UACR	RVEF	Weighted median	51	-0.049	0.103
UACR	RVEF	Inverse variance weighted	51	0.005	0.073
UACR	RVEF	Simple mode	51	0.214	0.210
UACR	RVEF	Weighted mode	51	-0.032	0.124
UACR	RVESV Indexed	MR Egger	51	0.134	0.164
UACR	RVESV Indexed	Weighted median	51	0.042	0.103
UACR	RVESV Indexed	Inverse variance weighted	51	0.066	0.079
UACR	RVESV Indexed	Simple mode	51	-0.050	0.193
UACR	RVESV Indexed	Weighted mode	51	0.016	0.111
UACR	RVESV LVESV ratio	MR Egger	51	-0.008	0.150
UACR	RVESV LVESV ratio	Weighted median	51	-0.099	0.109
UACR	RVESV LVESV ratio	Inverse variance weighted	51	-0.018	0.072
UACR	RVESV LVESV ratio	Simple mode	51	0.014	0.236
UACR	RVESV LVESV ratio	Weighted mode	51	-0.067	0.126
UACR	RVSV Indexed	MR Egger	51	0.006	0.165
UACR	RVSV Indexed	Weighted median	51	0.003	0.099
UACR	RVSV Indexed	Inverse variance weighted	51	0.103	0.080

UACR	RVSV Indexed	Simple mode	51	0.209	0.228
UACR	RVSV Indexed	Weighted mode	51	0.006	0.132

ng parameters of cardiac

pval	low_ci	up_ci
0.700	-1.076	0.721
0.029	-0.765	-0.042
0.007	-0.692	-0.108
0.192	-1.277	0.250
0.232	-0.955	0.228
0.800	-1.022	0.788
0.012	-0.858	-0.106
0.012	-0.679	-0.084
0.354	-1.373	0.487
0.095	-1.249	0.090
0.734	-1.228	0.864
0.526	-0.528	0.270
0.275	-0.533	0.152
0.994	-0.866	0.859
0.862	-0.809	0.677
0.965	-1.058	1.012
0.280	-0.671	0.194
0.534	-0.445	0.230
0.181	-1.856	0.340
0.217	-1.435	0.319
0.351	-0.516	1.464
0.208	-0.149	0.686
0.839	-0.291	0.359
0.693	-0.636	0.958
0.559	-0.466	0.864
0.355	-6.097	17.150
0.192	-1.392	6.930
0.196	-1.285	6.273
0.688	-10.017	6.601
0.564	-4.376	8.053
0.088	-0.104	1.703
0.255	-0.142	0.534
0.620	-0.225	0.378
0.752	-0.809	0.583
0.441	-0.331	0.764
0.147	-0.241	1.688
0.013	0.094	0.788
0.070	-0.024	0.610
0.170	-0.206	1.211
0.187	-0.181	0.953
0.087	-0.091	1.522
0.944	-0.375	0.349

0.241	-0.438	0.110
0.848	-0.674	0.820
0.737	-0.449	0.636
0.907	-0.767	0.864
0.250	-0.148	0.566
0.115	-0.052	0.479
0.550	-0.544	1.025
0.594	-0.429	0.752
0.771	-1.141	0.845
0.049	-0.823	-0.001
0.054	-0.643	0.006
0.250	-1.441	0.369
0.138	-1.284	0.170
0.885	-1.162	1.002
0.122	-0.086	0.732
0.521	-0.236	0.467
0.231	-0.347	1.461
0.373	-0.372	1.001
0.877	-0.922	1.081
0.691	-0.481	0.318
0.205	-0.537	0.115
0.476	-0.594	1.280
0.612	-0.597	1.018
0.872	-0.951	1.122
0.193	-0.640	0.129
0.091	-0.632	0.047
0.667	-0.952	0.608
0.532	-0.800	0.412
0.068	-0.044	1.684
0.405	-0.487	0.197
0.859	-0.264	0.317
0.362	-1.101	0.399
0.378	-0.897	0.338
0.870	-0.970	0.820
0.027	-0.792	-0.049
0.241	-0.465	0.117
0.088	-1.534	0.095
0.082	-1.368	0.070
0.155	-0.255	1.667
0.157	-0.098	0.609
0.360	-0.168	0.462
0.364	-0.441	1.213
0.371	-0.403	1.089
0.379	-0.525	1.392
0.313	-0.590	0.189
0.417	-0.446	0.185
0.654	-1.113	0.697
0.246	-1.276	0.322

0.107	-0.136	1.505
0.575	-0.477	0.265
0.625	-0.344	0.207
0.658	-1.108	0.698
0.729	-0.822	0.575
0.340	-0.159	0.053
0.003	-0.158	-0.032
0.000	-0.130	-0.042
0.092	-0.185	0.009
0.014	-0.166	-0.027
0.296	-0.153	0.044
0.043	-0.121	-0.002
0.011	-0.094	-0.012
0.131	-0.181	0.019
0.079	-0.143	0.004
0.774	-0.100	0.135
0.842	-0.053	0.065
0.558	-0.062	0.034
0.886	-0.099	0.115
0.854	-0.060	0.073
0.090	-0.227	0.010
0.619	-0.080	0.048
0.431	-0.072	0.031
0.338	-0.071	0.215
0.020	-0.166	-0.021
0.323	-0.058	0.184
0.571	-0.046	0.083
0.940	-0.053	0.049
0.177	-0.210	0.035
0.623	-0.054	0.091
0.513	-0.788	1.605
0.567	-0.462	0.844
0.804	-0.527	0.408
0.193	-2.130	0.388
0.365	-0.405	1.139
0.611	-0.082	0.140
0.767	-0.067	0.049
0.275	-0.072	0.020
0.909	-0.105	0.093
0.971	-0.067	0.064
0.482	-0.132	0.061
0.116	-0.101	0.011
0.365	-0.058	0.021
0.596	-0.137	0.078
0.185	-0.107	0.019
0.391	-0.059	0.155
0.514	-0.036	0.072
0.795	-0.051	0.039

0.838	-0.083	0.102
0.376	-0.031	0.085
0.943	-0.099	0.107
0.186	-0.099	0.019
0.087	-0.079	0.005
0.452	-0.143	0.062
0.273	-0.110	0.030
0.289	-0.043	0.153
0.055	-0.001	0.105
0.147	-0.010	0.067
0.173	-0.027	0.170
0.065	0.000	0.128
0.148	-0.029	0.228
0.247	-0.026	0.101
0.881	-0.051	0.060
0.631	-0.089	0.148
0.288	-0.034	0.121
0.456	-0.184	0.081
0.507	-0.086	0.043
0.817	-0.048	0.061
0.248	-0.053	0.217
0.464	-0.101	0.045
0.266	-0.236	0.062
0.388	-0.090	0.035
0.868	-0.058	0.068
0.869	-0.152	0.128
0.431	-0.099	0.041
0.727	-0.079	0.114
0.187	-0.090	0.018
0.194	-0.066	0.013
0.559	-0.122	0.065
0.304	-0.090	0.027
0.838	-0.090	0.111
0.549	-0.076	0.041
0.392	-0.059	0.023
0.539	-0.074	0.144
0.997	-0.070	0.070
0.841	-0.077	0.095
0.635	-0.064	0.039
0.724	-0.042	0.029
0.439	-0.112	0.047
0.503	-0.084	0.040
0.760	-0.117	0.161
0.441	-0.089	0.039
0.778	-0.065	0.048
0.257	-0.205	0.052
0.326	-0.111	0.036
0.841	-0.104	0.128

0.241	-0.095	0.024
0.103	-0.088	0.008
0.843	-0.114	0.093
0.394	-0.100	0.038
0.156	-0.306	1.942
0.212	-0.211	0.952
0.001	0.276	1.157
0.499	-0.987	2.029
0.584	-0.841	1.494
0.969	-1.188	1.141
0.918	-0.591	0.656
0.027	0.060	0.977
0.803	-2.115	1.637
0.805	-1.639	1.272
0.942	-12.862	13.852
0.029	0.697	13.297
0.024	0.810	11.328
0.119	-3.766	33.630
0.701	-11.768	17.503
0.585	-0.820	1.456
0.278	-0.256	0.889
0.027	0.058	0.949
0.539	-1.199	2.297
0.809	-1.159	1.486
0.133	-0.245	1.900
0.005	0.262	1.457
0.002	0.256	1.096
0.155	-0.444	2.824
0.088	-0.147	2.216
0.013	0.310	2.469
0.000	0.622	1.777
0.000	0.538	1.385
0.199	-0.471	2.285
0.009	0.311	2.081
0.864	-0.956	1.139
0.023	0.098	1.307
0.001	0.312	1.116
0.030	0.184	3.365
0.239	-0.450	1.815
0.371	-0.599	1.608
0.013	0.159	1.369
0.000	0.351	1.216
0.494	-1.039	2.156
0.458	-0.754	1.676
0.371	-0.606	1.631
0.155	-0.155	0.970
0.005	0.189	1.061
0.696	-1.715	1.144

0.646	-0.849	1.370
0.253	-0.491	1.875
0.196	-0.203	0.995
0.012	0.130	1.055
0.722	-1.947	1.349
0.870	-1.121	1.324
0.331	-0.528	1.572
0.824	-0.690	0.549
0.652	-0.317	0.507
0.598	-1.358	2.360
0.702	-1.434	0.965
0.051	0.002	2.269
0.553	-0.405	0.756
0.654	-0.347	0.553
0.704	-1.669	1.127
0.528	-0.682	1.330
0.086	-2.229	0.141
0.126	-1.000	0.123
0.775	-0.391	0.524
0.068	-0.114	3.557
0.103	-2.268	0.203
0.586	-1.218	0.687
0.327	-0.798	0.266
0.994	-0.372	0.375
0.694	-1.582	1.052
0.246	-1.408	0.359
0.186	-0.378	1.967
0.187	-0.210	1.077
0.104	-0.078	0.843
0.799	-1.513	1.965
0.497	-0.803	1.657
0.883	-1.049	1.219
0.237	-0.985	0.244
0.250	-0.705	0.184
0.408	-2.118	0.857
0.198	-1.719	0.352
0.071	-0.072	1.903
0.228	-0.219	0.919
0.946	-0.378	0.405
0.484	-0.953	2.016
0.207	-0.341	1.583
0.556	-1.371	0.737
0.305	-0.260	0.831
0.137	-0.100	0.729
0.707	-1.100	1.623
0.337	-0.492	1.440
0.495	-1.562	0.754
0.215	-0.232	1.033

0.747	-0.380	0.529
0.244	-0.644	2.547
0.297	-0.538	1.772
0.853	-0.343	0.284
0.861	-0.236	0.197
0.035	0.011	0.316
0.932	-0.477	0.437
0.876	-0.271	0.230
0.878	-0.309	0.362
0.893	-0.208	0.239
0.019	0.032	0.356
0.198	-0.164	0.819
0.717	-0.214	0.312
0.893	-0.283	0.325
0.794	-0.225	0.172
0.157	-0.040	0.250
0.522	-0.264	0.523
0.865	-0.258	0.216
0.225	-0.504	0.115
0.536	-0.274	0.143
0.168	-0.046	0.266
0.109	-0.093	1.012
0.322	-0.429	0.139
0.956	-0.267	0.283
0.670	-0.281	0.181
0.257	-0.211	0.056
0.143	-0.740	0.101
0.427	-0.372	0.156
0.665	-3.347	5.263
0.154	-0.653	4.124
0.096	-0.298	3.671
0.053	0.058	8.917
0.121	-0.519	4.781
0.722	-0.280	0.405
0.573	-0.137	0.248
0.174	-0.050	0.277
0.243	-0.135	0.546
0.457	-0.135	0.302
0.381	-0.413	0.157
0.521	-0.278	0.141
0.818	-0.122	0.154
0.884	-0.461	0.397
0.496	-0.321	0.155
0.504	-0.230	0.471
0.443	-0.126	0.288
0.483	-0.108	0.228
0.795	-0.402	0.307
0.444	-0.132	0.304

0.902	-0.270	0.307
0.180	-0.059	0.316
0.057	-0.004	0.273
0.135	-0.095	0.755
0.507	-0.160	0.326
0.531	-0.341	0.175
0.293	-0.277	0.084
0.974	-0.122	0.118
0.882	-0.450	0.386
0.367	-0.386	0.141
0.105	-0.050	0.586
0.248	-0.091	0.351
0.587	-0.111	0.197
0.725	-0.591	0.411
0.167	-0.081	0.484
0.246	-0.113	0.450
0.508	-0.144	0.291
0.242	-0.054	0.215
0.345	-0.191	0.553
0.300	-0.111	0.367
0.535	-0.401	0.207
0.429	-0.317	0.135
0.096	-0.268	0.022
0.134	-0.793	0.099
0.401	-0.356	0.141
0.702	-0.235	0.350
0.964	-0.188	0.197
0.099	-0.022	0.260
0.777	-0.335	0.449
0.974	-0.247	0.238
0.358	-0.436	0.156
0.636	-0.251	0.154
0.940	-0.138	0.149
0.313	-0.197	0.625
0.798	-0.275	0.211
0.417	-0.187	0.456
0.684	-0.161	0.245
0.405	-0.089	0.220
0.795	-0.428	0.327
0.889	-0.202	0.234
0.956	-0.303	0.286
0.362	-0.312	0.114
0.797	-0.159	0.122
0.952	-0.448	0.477
0.596	-0.314	0.180
0.973	-0.319	0.330
0.973	-0.191	0.197
0.196	-0.053	0.259

0.364	-0.238	0.655
0.963	-0.252	0.265
